# Evolution of folate biosynthesis and metabolism across algae and land plant lineages

**DOI:** 10.1038/s41598-019-42146-5

**Published:** 2019-04-05

**Authors:** V. Gorelova, O. Bastien, O. De Clerck, S. Lespinats, F. Rébeillé, D. Van Der Straeten

**Affiliations:** 1 Department of Biology, Laboratory of Functional Plant Biology, Ghent University, K.L Ledeganckstraat 35, 9000 Ghent, Belgium; 2 Department of Biology, Phycology Research Group, Ghent University, Krijgslaan 281, 9000 Gent, Belgium; 3 Laboratoire de Physiologie Cellulaire Vegetale, UMR168 CNRS-CEA-INRA-Universite Joseph Fourier Grenoble I, Bioscience and Biotechnologies Institute of Grenoble, CEA-Grenoble, 17 rue des Martyrs, 38054 Grenoble, Cedex 9 France; 4 Present Address: Department of Botany and Plant Biology, Laboratory of Plant Biochemistry and Physiology, University of Geneva, Quai E. Ansermet 30, 1211 Geneva, Switzerland

## Abstract

Tetrahydrofolate and its derivatives, commonly known as folates, are essential for almost all living organisms. Besides acting as one-carbon donors and acceptors in reactions producing various important biomolecules such as nucleic and amino acids, as well as pantothenate, they also supply one-carbon units for methylation reactions. Plants along with bacteria, yeast and fungi synthesize folates *de novo* and therefore constitute a very important dietary source of folates for animals. All the major steps of folate biosynthesis and metabolism have been identified but only few have been genetically characterized in a handful of model plant species. The possible differences in the folate pathway between various plant and algal species have never been explored. In this study we present a comprehensive comparative study of folate biosynthesis and metabolism of all major land plant lineages as well as green and red algae. The study identifies new features of plant folate metabolism that might open new directions to folate research in plants.

## Introduction

**Figure 1:**
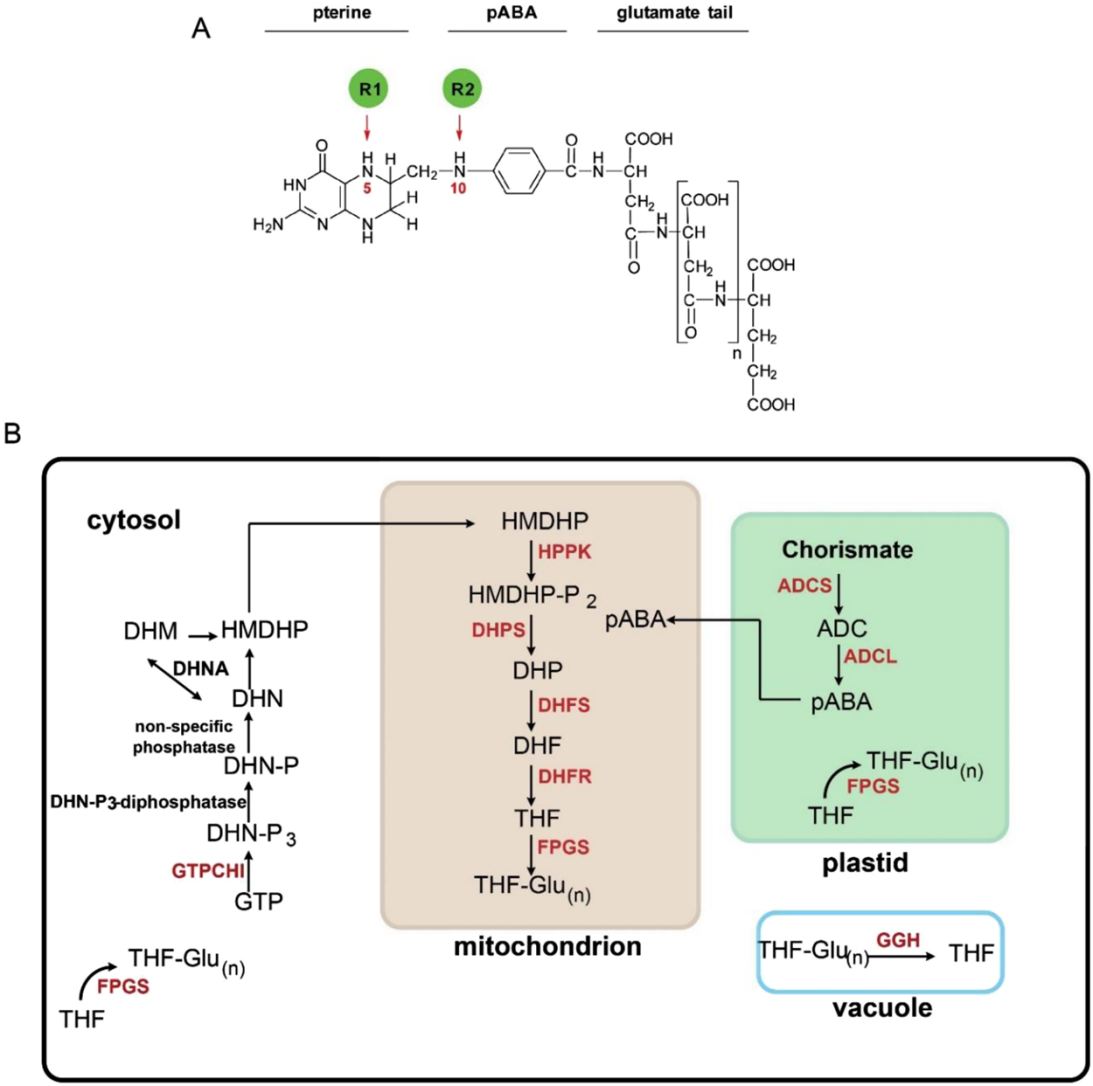
Structure of the THF molecule and folate biosynthesis. (**A**) Structure of THF molecule. **(B)** Folate biosynthesis in higher plants. Precursors: GTP, guanosine triphosphate; DHN-P_3_, dihydroneopterin triphosphate; DHN-P, dihydroneopterin monophosphate; DHN, dihydroneopterin; DHM, dihydromonapterin; HMDHP, 6-hydroxymethyldihydropterin; HMDHP-P_2_, 6-hydroxymethyldihydropterin pyrophosphate; DHP, dihydropteroate; DHF, dihydrofolate; THF, tetrahydrofolate; THF-Glu_(n)_, tetrahydrofolate polyglutamate; ADC, aminodeoxychorismate; pABA, para-aminobenzoic acid. Enzymes: GTPCHI, GTP cyclohydrolase I; DHN-P_3_-diphosphatase, dihydroneopterin triphosphate pyrophosphatase; DHNA, dihydroneopterine aldolase; HPPK, HMDHP pyrophosphokinase; DHPS, dihydropteroate synthase; DHFS, dihydrofolate synthetase; DHFR, dihydrofolate reductase; FPGS, folylpolyglutamate synthetase; ADCS, aminodeoxychorismate synthase; ADCL, aminodeoxychorismate lyase; GGH, gamma-glutamyl hydrolase.

Tetrahydrofolate (THF) and its derivatives, known as folates or B9 vitamins, are essential elements in the metabolism of all living organisms, except methanogenic and sulfate-reducing Archaea that use tetrahydromethanopterin (H4MPT) or its derivative tetrahydrosarcinapterin (H4SPT) in their C1 metabolism^1^. The THF molecule is composed of a pterin moiety, para-aminobenzoic acid (pABA) and a glutamate tail. To the N5 and N10 positions of THF, one-carbon (C1) units of various oxidation states are attached (Fig. 1A). Essentially, folates act as donors and acceptors of C1 units in C1 transfer reactions that are involved in many major metabolic processes. Folates are used in synthesis of nucleic acids (with the exception of cytosine), amino acids, pantothenate, formyl-methionyl-tRNA, and S-adenosyl-methionine (SAM). While animals are mainly depend on their dietary sources for folate supply, bacteria, fungi and plants synthesize folates *de novo*.

In plants, folate biosynthesis localizes to three subcellular compartments. Pterin and pABA are synthesized in cytosol and plastids, respectively. Subsequently, the THF molecule is assembled in mitochondria, and the glutamate tail is added (Fig. 1B).

Like other folate-producing organisms, plants synthesize p-ABA in two steps. The first step is catalysed by aminodeoxychorismate synthase (ADCS) (Fig. 1B). Similar to their fungal^2,3^ and protozoan^4^ counterparts, Arabidopsis and tomato ADCSs exist as a bifunctional protein with two functional domains: the glutamine amidotransferase domain (GAT) homologous to *E. coli* PabA and the chorismate binding domain homologous to *E. coli* PabB^5,6^.

The second step in the biosynthesis of pABA is mediated by aminodeoxychorismate lyase (ADCL) that catalyses the beta-elimination of pyruvate and aromatization of the ADC ring to produce pABA^7,8^.

Synthesis of the pterin moiety starts with the conversion of GTP into dihydroneopterin triphosphate and formate, catalysed by GTP cyclohydrolase I (GTPCHI) (Fig. 1B). Studies on spinach, tomato and Arabidopsis demonstrated that plant GTPCHI has two functional domains that are essential for the enzymatic activity, as neither domain has a complete set of the residues required for substrate binding and catalysis^9–11^. Dihydroneopterin undergoes dephosphorylation that proceeds in two steps. The first step is the removal of pyrophosphate by cytosolic Nudix hydrolase^12^. The second step is catalysed by a non-specific phosphatase^13^. The final step of the synthesis of the pterin moiety is mediated by dihydroneopterin aldolase (DHNA), which cleaves the lateral side chain of dihydroneopterin, releasing glycolaldehyde and 6-hydroxymethyldihydropterin (HMDHP)^14^.

Synthesis of THF in mitochondria starts with pyrophosphorylation of HMDHP and its subsequent coupling with pABA that results in the formation of dihydropteroate (DHP). These two reactions are mediated by HMDHP pyrophosphokinase (HPPK) and dihydropteroate synthase (DHPS) enzymatic activities. These two enzymes were found to be coupled on a single polypeptide in several plant species, such as pea^15,16^, Arabidopsis^17^, rice and wheat^18^. DHP is further converted to dihydrofolate (DHF) in the reaction mediated by dihydrofolate synthetase (DHFS), which attaches the first glutamate residue to the carboxyl moiety of pABA in DHP (Fig. 1B). The penultimate step of folate biosynthesis is catalysed by dihydrofolate reductase (DHFR) that reduces DHF into THF. DHFR activity can be performed by a monofunctional enzyme as in mammals, or by a bifunctional protein, coupled with thymidylate synthase (TS), as in protozoa. Among plant species, bifunctional DHFR-TS genes were described in carrot^19^, pea^20^, maize^21^ and Arabidopsis^22^.

In the pathway leading to THF-Glu_n_ synthesis, two reactions mediate the attachment of glutamate. The first reaction is catalysed by DHFS. In the second reaction catalysed by folylpolyglutamate synthetase (FPGS), the polyglutamate tail of THF-Glu_n_ is formed by the sequential addition of glutamate residues to THF-Glu_1_ (Fig. 1B). The polyglutamate tail can be shortened or removed by the activity of vacuolar gamma-glutamyl hydrolases (GGH) that play an important role in regulation of folate homeostasis^23–25^.

Enzymes involved in the interconversion of various folate species are important components of folate metabolism not only for folate-producing organisms but also for those that rely on their diet for the folate supply^26^. These enzymes orchestrate folate metabolism and therefore regulate the flux of C1 units in response to environmental or developmental cues. Therefore, their activities have to be tightly regulated.

**Figure 2:**
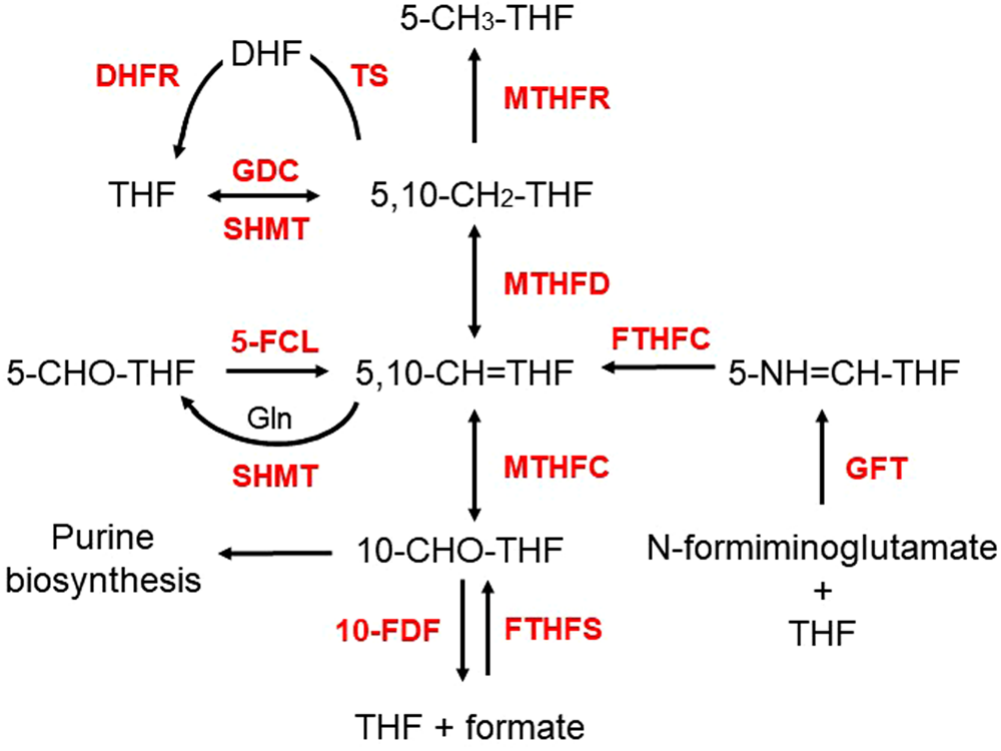
Interconversion of folate species. THF, tetrahydrofolate; 5-CH_3_-THF, 5-methyltetrahydrofolate; 5,10-CH_2_-THF, 5,10-methylenetetrahydrofolate; 5,10-CH^+^-THF, 5,10-methenyltetrahydrofolate; 5-CHO-THF, 5-formyltetrahydrofolate; 10-CHO-THF, 10-formyltetrahydrofolate. SHMT, serine hydroxymethyl transferase; GDC, glycine decarboxylase complex, FTHFC, formiminotetrahydrofolate cyclodeaminase; 5-FCL, 5-formyltetrahydrofolate cycloligase; GFT, glutamate formiminotransferase; FTHFS, 10-CHO-THF synthetase; MTHFD-MTHFC, 5,10- CH_2_-THF dehydrogenase/5,10-CH^+^-THF cyclohydrolase; MTHFR, methylenetetrahydrofolate reductase; 10-FDF, 10-CHO-THF deformylase.

There are several sources of one-carbon units for folate metabolism in plants. Serine is an important entry point to 5,10-methylene-tetrahydrofolate (5,10-CH_2_-THF) in all eukaryotes. Loading of THF occurs upon conversion of Ser into Gly, mediated by SHMT, which hooks a methylene group onto THF, in a reversible reaction (Fig. 2)^27,28^. SHMT is assumed to operate in the cytosol, plastids, and mitochondria^29^. In the latter compartment, the production of 5,10-CH_2_-THF is mainly mediated by Gly cleavage through the action of the T-protein of glycine decarboxylase (GDC), a reaction of paramount importance in photorespiration, typical for C3 plants. (Fig. 2). Another source of one-carbon units for folate metabolism is formate. Formate is converted to 10-formyl-tetrahydrofolate (10-CHO-THF) in the reaction catalysed by 10-CHO-THF synthetase (FTHFS)^30^. Subsequently, 10-CHO-THF can be metabolized in purine biosynthesis or converted to 5,10-methenyl-THF (5,10-CH^+^-THF) via 5,10-CH_2_-tetrahydrofolate dehydrogenase/5,10-CH^+^-tetrahydrofolate cyclohydrolase (MTHFD-MTHFC) (Fig. 2)^31^. MTHFC and MTHFD activities were found to be coupled on a bifunctional polypeptide in pea^31^ and Arabidopsis^32^.

The reverse reaction resulting in hydrolysis of 10-formyl-THF into THF and formate is carried out by 10-CHO-THF deformylase (10-FDF) (Fig. 2). Two *10-FDF* genes were characterized in Arabidopsis and found to be crucial for photorespiration^32^.

There are two alternative ways to generate 5,10-CH^+^-THF. The first one involves 5-formyltetrahydrofolate cycloligase (5-FCL) that catalyses the conversion of 5-formyltetrahydrofolate (5-CHO-THF) into 5,10-CH^+^-THF (Fig. 2). Unlike other folate species, 5-CHO-THF is not used as a one-carbon (C1) donor but acts as a potent inhibitor of SHMT and several other enzymes of C1 metabolism^33^. Therefore, 5-CHO-THF is considered to be a regulator of C1 metabolism. The second way to generate 5,10-CH^+^-THF involves glutamate formiminotransferase (GFT), a folate-dependent enzyme that takes part in histidine degradation in mammals and some bacteria. GFT mediates the transfer of a formimino group from formiminoglutamate to THF, producing 5-formimino-THF which is then converted to 5,10-CH^+^-THF by the action of formiminotetrahydrofolate cyclodeaminase (FTHFC) (Fig. 2)^34^. Biochemical and genetic characterization of plant GFT is still poor^35^.

The flux of one-carbon units from folate metabolism demands the activity of methylenetetrahydrofolate reductase (MTHFR) that catalyses the reduction of 5,10-CH_2_-THF to 5-methyltetrahydrofolate (5-CH_3_-THF) which enters the methyl cycle (Fig. 2).

While biochemistry of plant folate biosynthesis and metabolism is considered well characterized, their compartmentalisation and regulation await further studies. The task could become utmost challenging considering that the regulation of folate metabolism might differ between species. Examples showing that biofortification strategies are not equally efficient for all crop species corroborate this notion^36^. Unfortunately, genes of folate biosynthesis and metabolism were characterised only in few model plant species. Increasing availability of genome-scale data provides an excellent opportunity to trace the origin of the pathway components in algae, to identify possible differences in regulation and compartmentalisation of folate metabolism in various plant species and to plan future biofortification approaches that will take into account individual features of folate metabolism of a given species. To identify the possible differences and to trace their emergence during evolution, we ventured into a comparative genomic and phylogenetic study including all major land plant lineages as well as red and green algae. Our study provides a comprehensive view on the folate biosynthesis and metabolism throughout the plant kingdom and points out novel aspects of folate metabolism. We demonstrate that bifunctionality of enzymes in folate biosynthesis and enzymes involved in interconversion of folate species is a common feature for algae and higher plant species. Our comparative study shows that the number of genes, localization and the structure of the isoforms they encode is highly conserved across algae and land plants. Moreover, our findings underscore the notion that folate metabolism of different subcellular compartments is characterized by specific sources of one-carbon units.

## Results

### Taxon sampling for the analysis and identification of putative orthologs in folate metabolism

Our comparative study is largely focused on land plant species. A moss *Physcomitrella patens*, a lycophyte *Selaginella moellendorfii* and an ancient angiosperm species *Amborella trichopoda* were included in the study. Four monocotyledonous genomes, namely, *Musa acuminata*, *Oryza sativa*, *Sorghum bicolor, Triticum aestivum* and *Zea mays* and sixteen dicotyledonous genomes, namely, *Arabidopsis thaliana*, *Brassica rapa*, *Capsella rubella*, *Carica papaya*, *Citrus sinensis*, *Cucumis sativus*, *Fragaria vesca*, *Glycine max*, *Gossipium raimondii*, *Linum usitatissimum*, *Medicago truncatula*, *Phaseolus vulgaris*, *Populus trichocarpa*, *Solanum lycopersicum*, *Solanum tuberosum* and *Vitis vinifera* were analysed.

Several green algae members of Charophyta, namely, Ostreoc*occus tauri*, *Ostreococcum lucimarinus* and *Micromonas pusilla* (Prasinophyceae), *Chlamydomonas reinhardtii* and *Volvox carteri* (Chlorophyceae), *Chlorella vulgaris* and *Chlorella variabilis* (Trebouxiophyceae), *Ulva mutabilis* (Ulvophyceae), and *Klebsormidium flaccidum* (Klebsormidiophyceae) were used in the study. Two red algae, namely, *Chondrus crispus* (Florideophyceae) and *Cyanidioschyzon merolae* (Cyanidiophyceae) were also covered.

To identify putative orthologs of folate pathway genes in the species listed above, full-length protein sequences from *Arabidopsis thaliana* were used as a query to run a BLAST protein sequence alignment^37^. To infer putative orthology the selected protein sequences were scrutinized for the presence of functional protein domains using the Pfam database^38^. The selected protein sequences were also surveyed for the presence of subcellular targeting signals and for the pattern of conserved protein motifs.

### ADCS

**Figure 3:**
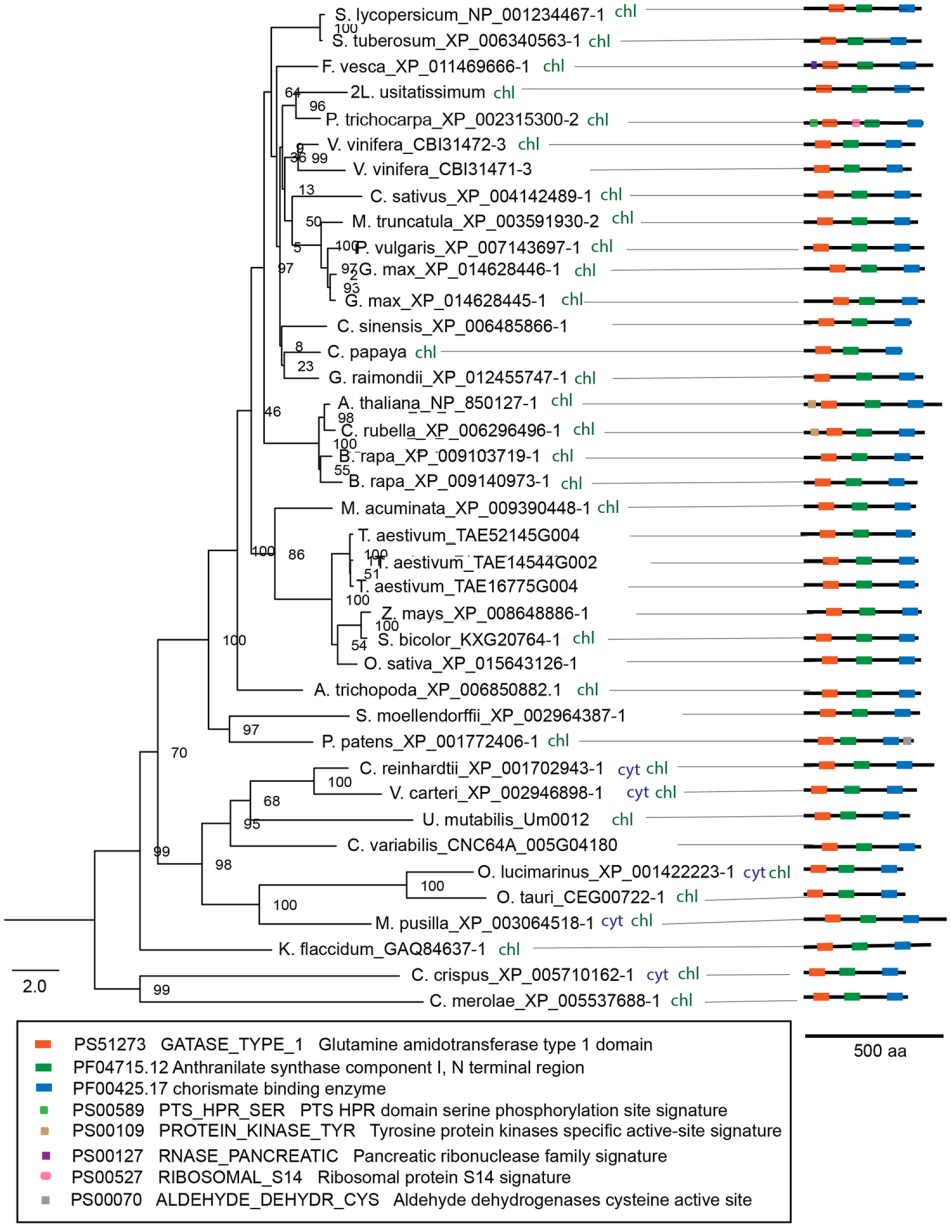
Phylogenetic analysis, subcellular localization and domain composition of ADCS proteins. Species names are followed by protein identifiers. The bar indicates the mean distance of 2.0 changes per amino acid residue. The numbers at the branching points indicate the percentage of times that each branch topology was found during bootstrap analysis (n = 1000). The box contains predicted functional domains. Schemes on the right represent domain organisation of analysed proteins (colored boxes represent functional domains, lengths of black lines correspond to lengths of proteins. The scale bar below shows protein containing 500 amino acids). Cyt, cytosolic localization; chl, plastidial localization. Indication of double localization (e.g. cyt chl) for a single protein implies its probable localization to both compartments.

Our comparative analysis shows that ADCS is encoded by a single copy gene in genomes of all analysed algal species as well as in most analysed higher plant species (Fig. 3). Being in line with reported targeting of Arabidopsis ADCS to chloroplasts^7^ our prediction of the subcellular localization suggested that ADCS homologs in algae and higher plants possess chloroplast targeting signals (Fig. 3).

Examination of selected protein sequences for the presence of functional domains revealed that besides glutamine amidotransferase domain (GAT) and chorismate binding domains, algal and higher plant ADCS bear homology to the N terminal region domain of anthranilate synthase component I (alpha subunit) (Fig. 3). ADCS proteins from *P. patens*, *P. trichocarpa*, *F. vesca*, *A. thaliana* and *C. rubella* contain one or two additional domains (Fig. 3), possibly manifesting a process of neofunctionalization of ADCS within these species. The motif pattern (sequence within which motifs appear) is conserved throughout algal and land plant species (Supplemental Fig. 1).

### ADCL

**Figure 4:**
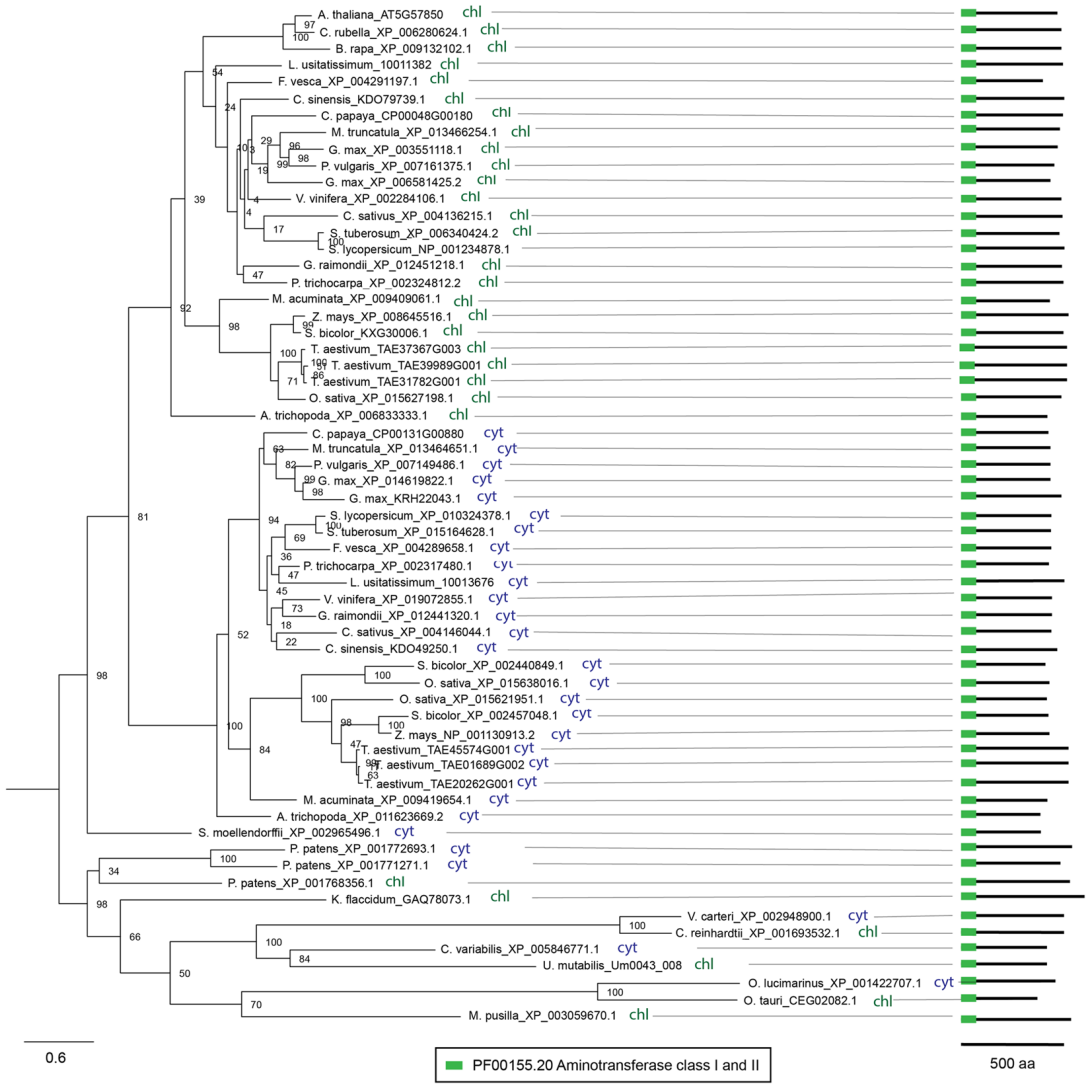
Phylogenetic analysis, subcellular localization and domain composition of ADCL proteins. Species names are followed by protein identifiers. The bar indicates the mean distance of 0.6 changes per amino acid residue. The numbers at the branching points indicate the percentage of times that each branch topology was found during bootstrap analysis (n = 1000). Schemes on the right represent domain organisation of analysed proteins (color boxes represent functional domains, lengths of black lines correspond to lengths of proteins. The scale bar below shows protein containing 500 amino acids). The box contains predicted functional domains. Cyt, cytosolic localization; chl, plastidial localization.

While algae possess a single copy of *ADCL*, the majority of higher plant species contains two or three *ADCL* genes (Fig. 4). Like those of algae, the genomes of *A. thaliana*, *C. rubella* and *B. rapa* also encode a single ADCL gene, indicating loss of the second ADCL gene during evolution of *Brassicaceae*. Our phylogenetic study revealed that ADCL genes of higher plants fall into two clades (Fig. 4). Interestingly, each clade contains an ADCL homolog from every analysed higher plant species. The presence of an ADCL gene from the *A. trichopoda* genome in both clades suggests that the divergence of ADCL genes occurred before diversification of flowering plants. The prediction of the subcellular localization revealed that all proteins from the clade containing *A. trichopoda*_XP_006833333.1 have a chloroplast localization signal, whereas proteins from the clade containing the *A. trichopoda*_XP_011623669.2 isoform are exclusively cytosolic (Fig. 4). Moreover, the *P. patens* genome also encodes two cytosolic and one plastidial ADCL isoform, further confirming that duplication and diversification of ADCL genes occurred during early evolution of land plants (Fig. 4). The plastidial localization of GFP-tagged ADCL from Arabidopsis has been previously demonstrated^7^, while a confirmation for cytosolic ADCL remains unreported.

All analysed ADCL polypeptides contain a single Aminotransferase class I and II (PF00155.20) domain (Fig. 4). The motif pattern is well conserved among the analysed algal and higher plant species (Supplemental Fig. 2).

### GTPCHI

*In silico* analysis demonstrates that while algae have a single GTPCHI isoform, the majority of analysed higher plant species possesses two or more GTPCHI isoforms (Supplemental Fig. 3). Prediction of subcellular targeting suggested cytosolic localization of all analysed proteins, supporting the experimental data for Arabidopsis and tomato GTPCHI proteins^11^.

Our findings reveal the presence of two GTPCHI domains throughout algae and higher plant lineages, with the exception of *L. usitatissimum* GTPCHI that possesses three domains, the first being duplicated (Supplemental Fig. 3). Remarkably, the two GTPCHI domains show very different conserved motif composition, that further suggests their strong divergence (Supplemental Fig. 4).

### HPPK-DHPS

Genomes of the majority of analysed algae and higher plant species contain a single HPPK-DHPS gene (Supplemental Fig. 5). Prediction of their subcellular localization suggested mitochondrial localization of the majority of studied HPPK-DHPS isoforms (Supplemental Fig. 5). This finding is in line with experimentally determined mitochondrial targeting of HPPK-DHPS proteins in pea^15^ and Arabidopsis^39^.

Our comparative study shows that the bifunctionality is conserved across algae and land plant species, as HPPK and two DHPS domains (DHPS1 and DHPS2) were identified in most analysed proteins (Supplemental Fig. 5). While all higher plant species possess two DHPS domains, some algae and lower land plant species (*S. moellendorfii* and *P. patens*) have only one DHPS domain. *P. trichocarpa*, *C. rubella* and *S. bicolor* proteins contain additional domains (Supplemental Fig. 5). Our *in silico* protein analysis indicates that the motif pattern is well conserved (Supplemental Fig. 6).

### DHFS

Phylogenetic analysis revealed that DHFS is encoded by a single copy gene in genomes of most analysed species, with the exception of *G. max* and *M. truncatula* genomes which are characterized by high polyploidy (Supplemental Fig. 7). Being in line with experimental evidence for Arabidopsis DHFS^39^, our analysis suggested that DHFS in higher plant species localizes to mitochondria, while in algae it can also localize to cytosol or plastids (Supplemental Fig. 7).

*In silico* analysis revealed that DHFS proteins contain the same set of functional domains as FPGS polypeptides (see below): FPGS1, FPGS2 and muramyl ligase (Supplemental Fig. 7). Although DHFS and FPGS share similar biochemical function, sequences of these two proteins diverged immensely. The divergence is further illustrated by the protein sequence analysis which demonstrates that DHFS and FPGS proteins have very different patterns of conserved motifs (compare Supplemental Figs 8 and 11). It has been previously reported that Arabidopsis *DHFS* is more similar to the *DHFS-FPGS* gene from *E. coli* than to Arabidopsis *FPGS* genes^39^.

### DHFR

Our phylogenetic analysis demonstrates that single-copy *DHFR-TS* genes are present in algal genomes, while *DHFR-TS* in higher plant species is encoded by two or more genes (Supplemental Fig. 9). Prediction of subcellular localization suggested that DHFR-TS isoforms can be targeted to multiple compartments (Supplemental Fig. 9), corroborating our previous finding of multiple subcellular targeting of DHFR-TS isoforms in Arabidopsis^40^. The data in Supplemental Fig. 9 demonstrate that genomes of all analysed higher plant and algal species encode bifunctional DHFR-TS enzymes.

A previous study of Arabidopsis DHFR-TS demonstrated that one of the isoforms lacks enzymatic activity and acts as an inhibitor of its family members. Phylogenetic analysis suggested that such inhibitory isoforms may be also present in Arabidopsis’ closest relatives, *B. rapa* and *C. rubella*^40^. The present study shows that the inhibitory isoforms lack the last protein motif, while all other analysed DHFR-TS proteins retain it (Supplemental Fig. 10). It is plausible that this motif is important for the DHFR and TS activities, possibly being essential for a proper protein conformation.

### FPGS

**Figure 5:**
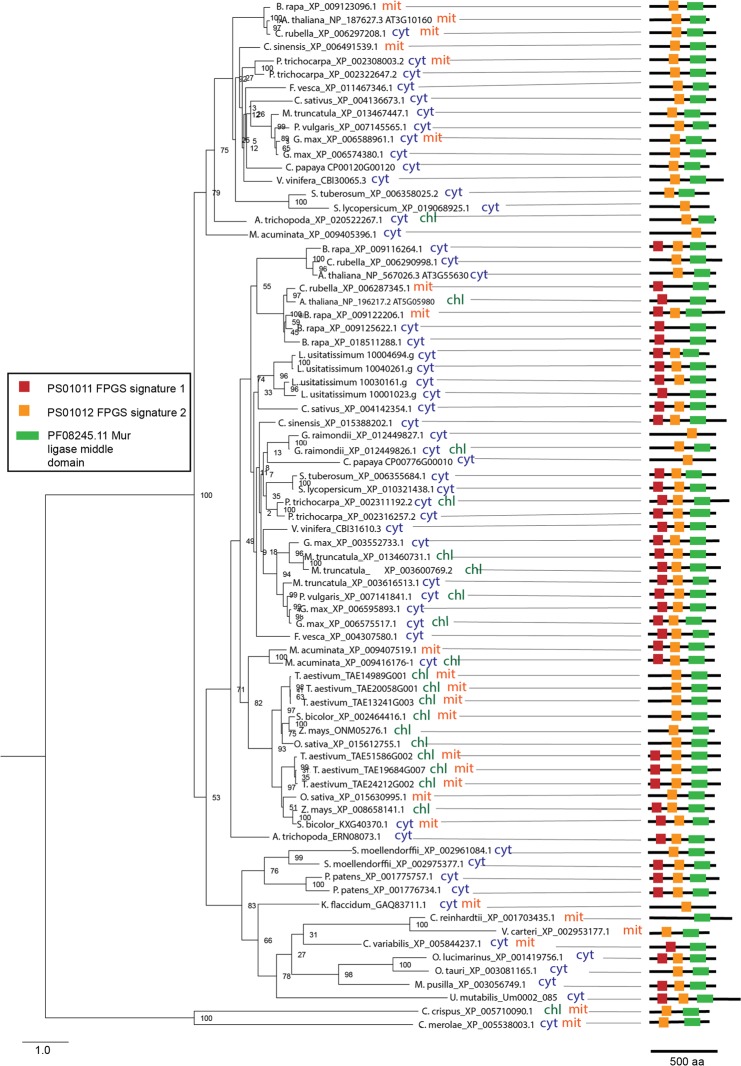
Phylogenetic analysis, subcellular localization and domain composition of FPGS proteins. Species names are followed by protein identifiers. The bar indicates the mean distance of 1.0 change per amino acid residue. The numbers at the branching points indicate the percentage of times that each branch topology was found during bootstrap analysis (n = 1000). Schemes on the right represent domain organisation of analysed proteins (color boxes represent functional domains, lengths of black lines correspond to lengths of proteins. The scale bar below shows protein containing 500 amino acids). The box contains predicted functional domains. Cyt, cytosolic localization; chl, plastidial localization; mit, mitochondrial localization. Indication of double localization (e.g. cyt chl) for a single protein implies its probable localization to both compartments.

The present study shows that, unlike algae, land plant species possess two or more FPGS isoforms. Being in line with experimental data^39,41^ our prediction of subcellular localization suggests that the isoforms localize to multiple subcellular compartments, namely, mitochondria, cytosol and plastids (Fig. 5). Analysis of protein sequences revealed the presence of three functional domains in FPGS polypeptides: a muramyl ligase domain and two FPGS domains (FPGS1 and FPGS2). Our phylogenetic analysis demonstrated that FPGS of higher plants branch into two clades, each containing a gene from *A. trichopoda* (Fig. 5). The two clades differ by their domain composition. The clade containing the *A. trichopoda_*ERN08073.1 isoform retains both FPGS functional domains, while the clade comprising the *A. trichopoda*_XP_020522267 isoform lacks the FPGS1 domain. In previous work it was shown that mit *AtFPGS*, lacking the FPGS1 domain, could complement a yeast FPGS mutant^39^, indicating that the absence of this domain did not impair the activity. Prediction of targeting signals suggests that subcellular localization of FPGS proteins varies within each clade (Fig. 5). This indicates that localization signals of FPGS were gained in each lineage individually.

### GGH

Our analysis shows that GGH is encoded by multiple genes in several algal and land plant genomes (Supplemental Fig. 12). Interestingly, GGH is the only enzyme of the folate pathway that is encoded by several genes in genomes of algae species. This observation corroborates the notion that the enzyme plays an important role in regulation of folate pool^24^ and highlights the demand of a more profound investigation of its function. Being in line with previous studies of GGH enzymes from Arabidopsis and tomato^23,25^ our subcellular localization analysis predicted existence of secretory signals in all analysed GGH proteins (Supplemental Fig. 12). Protein analysis revealed that all analysed GGH proteins possess a single peptidase C26 (PF07722.13) functional domain (Supplemental Fig. 12) and show a highly conserved motif pattern (Supplemental Fig. 13).

### GDC and SHMT

The *in silico* analysis revealed that the GDC T-protein is encoded by a single gene in algal genomes, while land plant species possess single or multiple GDC T-protein genes (Supplemental Fig. 14). The predicted mitochondrial localization of the analysed GDC isoforms is in line with its role in photorespiration^42^. Protein analysis suggested the presence of two functional domains: an aminomethyltransferase folate-binding domain (PF01571.20) and a glycine cleavage T-protein C-terminal barrel domain (PF08669.10). Phylogenetic analysis suggested that genomes of both algae and land plants encode multiple SHMT isoforms that fall into three big clades. According to our prediction of subcellular localization, each clade comprises isoforms with either mitochondrial, cytosolic or plastidial localization, confirming previous findings in pea and potato^20,28^ (Supplemental Fig. 15).

### FTHFS

Our comparative study demonstrates that FTHFS is encoded by a single gene in all examined species, with the exception of *G. max*, *L. usitatissimum* and *P. trichocarpa* that have two *FTHFS* genes (Supplemental Fig. 16). In agreement with the finding that FTHFS activity is associated with the cytosolic fraction of pea cotyledons, our prediction of subcellular localization suggested that FTHFS resides exclusively in the cytosol of all analysed algae and land plant species (Supplemental Fig. 16). Protein analysis revealed that all analysed FTHFS proteins possess a single formate-tetrahydrofolate ligase (PF01268.18) functional domain (Supplemental Fig. 16) and show a highly conserved motif pattern (Supplemental Fig. 17).

### 10-FDF

Phylogenetic analysis reveals the presence of single genes encoding 10-FDF in algal genomes, whereas land plant species might possess several 10-FDF genes (Supplemental Fig. 18). Being in agreement with reported mitochondrial targeting of Arabidopsis 10-FDF isoforms and their role in photorespiration^32^, our subcellular localization prediction suggests mitochondrial localization of the analysed 10-FDF proteins (Supplemental Fig. 18). Our protein analysis demonstrates that all analysed 10-FDF bear a single formate-tetrahydrofolate ligase domain (PF01268.18) and have a highly similar motif pattern (Supplemental Figs 18 and 19).

### MTHFD-MTHFC

*In silico* analysis indicates that higher plant species contain multiple MTHFD-MTHFC isoforms that fall into three clades, according to their predicted subcellular localization in cytosol, mitochondria and plastids, each comprising a gene from *A. trichopoda* (Supplemental Figs 20–22). Each clade includes a MTHFD-MTHFC homolog from *A. trichopoda*, suggesting their emergence early during speciation of flowering plants. Analysis of protein sequences showed that all analysed proteins possess the NAD(P)-binding (PF02882.18) and MTHFD-MTHFC catalytic (PF00763.22) domains, with the exception of two *L. usitatissimum* plastidial MTHFD-MTHFC isoforms that bear an additional domain identified as ACT (Supplemental Figs 20–22). Our study shows that the motif pattern is well conserved (Supplemental Figs 23–25).

### 5-FCL

Supplemental Fig. 26 illustrates a comparative analysis for 5-FCL. It is encoded by a single gene in algal genomes, in contrast to some higher plant species, which contain two 5-FCL coding genes. As *P. patens* genome contains two 5-FCL genes, its duplication presumably occurred during speciation of land plants or earlier. Prediction of subcellular localization has shown that the analysed 5-FCL proteins are mainly targeted to mitochondria but can also reside in plastids and in the cytosol (Supplemental Fig. 26). The prediction is in line with the experimentally determined mitochondrial localization of 5-FCL activity in pea^43^. All analysed proteins have a similar motif pattern (Supplemental Fig. 27) and bear a single 5-formyltetrahydrofolate cycloligase (PF01812.19) functional domain (Supplemental Fig. 26); no other domains could be identified.

### GFT

GFT appears to be encoded by a single gene in algae, whereas genomes of land plants can contain single or multiple GFT genes (Supplemental Fig. 28). Subcellular localization prediction suggested that GFT isoforms predominantly reside in the cytosol in higher plants, but can also be targeted to both mitochondria and plastids, as for instance, in *A. trichopoda*, *C. sinensis*, *L. usitatissimum*, *O. sativa*, *P. trichocarpa* and *Z. mays* (Supplemental Fig. 28). Protein analysis demonstrated that GFT proteins possess a single formiminotransferase domain (PF07837.11) and show a well conserved motif pattern throughout algal and land plant lineages (Supplemental Fig. 29). *G. raimondii*, *L. usitatissimum*, *C. sinensis* and *C. sativus* bear an additional domain.

### MTHFR

Our comparative analysis illustrates that unlike those of algal species, genomes of most land plant species encode several MTHFR isoforms (Supplemental Fig. 30). Prediction of subcellular localization suggested that MTHFR of algae and land plant species reside in the cytosol (Supplemental Fig. 30). The cytosolic localization of MTHFR is in line with the assumption that the cytosol is the predominant location of the one-carbon flux from folate metabolism toward methionine production^44^. Analysis of protein sequences demonstrated that MTHFR of algae and land plants contain a single MTHFR functional domain (Supplemental Fig. 28) and show a well conserved motif pattern (Supplemental Fig. 31).

### Evolution of DHFR-TS and HPPK-DHPS in plants versus other organisms

In the green lineage, the assembly of the folate molecule occurs within the mitochondria (Fig. 1) and involves two bifunctional enzymes, DHFR-TS and HPPK-DHPS. This is not always the case in other organisms where these activities can be driven by monofunctional enzymes. It is well recognized that the fusion between DHFR and TS is a milestone in evolution, illustrating the separation between unikonts and bikonts^45^. To test whether the other bifunctional enzyme of the folate biosynthesis pathway follows the same evolutionary history as DHFR-TS, we compared the phylogenetic evolution of the TS and DHPS domains in wide range of organisms, including bacteria, protists (Alveolata, fungi, red algae (Rhodophyta), Rhizaria, stramenopiles), plants and animals.

### TS

**Figure 6:**
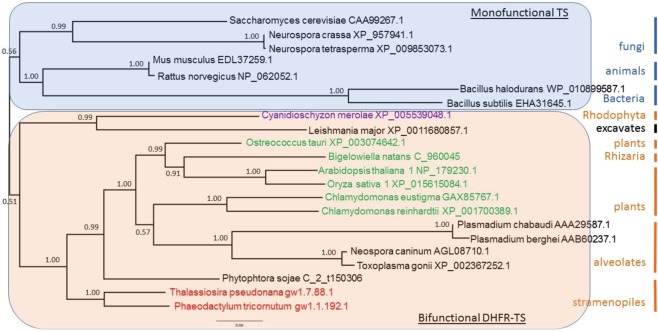
Phylogenetic trees of the protein sequences of the TS domain constructed using maximum likelihood method Phyml (see text). The selected model using Bayesian information criterion was LG + G + I with gamma shape parameter estimate = 1.117 and the proportion of invariable site estimate = 0.207. Nodes values represents the Bayesian posterior probabilities branch support. The species colour code corresponds to the type of plastid pigments, as follows: purple, chlorophyll a; green, chlorophyll a and b; red, chlorophyll a and c.

The phylogeny based on the maximum likelihood (ML) for the TS domain from 22 species representative of the various kingdoms clearly illustrates the evolutionary separation between organisms having monofunctional TS (unikonts) versus those having bifunctional DHFR-TS (bikonts) (Fig. 6).

**Figure 7:**
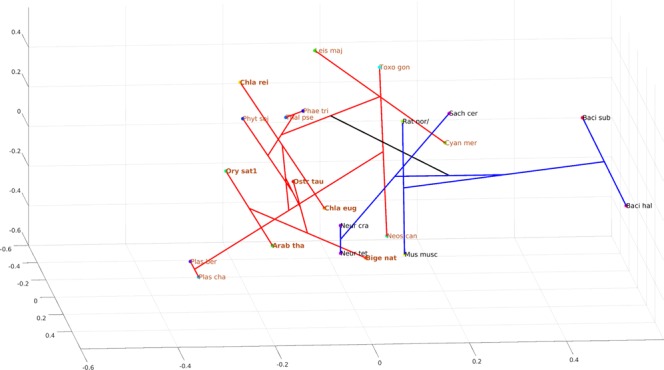
Phylogenetic representation in the protein sequence space for TS domains. Non-linear mapping methods are designed to offer a configuration of points in this multidimensional space that is representative of the observed distances. With this method, axes become arbitrary and every rotation or symmetry is admissible. In this figure, the distance between two data points on the figure tends to display the distance between species. Links between points show the ML phylogenic tree presented in the Fig. 6. Blue branches: monofunctional TS, red branches: bifunctional TS. Brown letters, bikont cluster; brown letters in bold, the green lineage; black letters, unikont cluster. Abbreviations for genus names: Arab_tha1, *Arabidopsis thaliana* 1; Ory_sat1, *Oryza sativa* 1; Chla_rei, *Chlamydomonas reinhardtii*; Chla_eug, *Chlamydomonas eustigma*; Ostr_tau, *Ostreococcus tauri*; Plas_ber, *Plasmodium berghei*; Plas_cha, *Plasmodium chabaudi*; Toxo_gon, *Toxoplasma gondii*; Neos_can, *Neospora caninum*; Cyan_mer, *Cyanidioschyzon merolae*; Leis_maj, *Leishmania major* strain Friedlin; Phae_tri, *Phaeodactylum tricornutum*; Thal_pse, *Thalassiosira pseudonana*; Phyt_soj, *Phytophthora sojae*; Mus_musc, *Mus musculus*; Rat_nor, *Rattus norvegicus*; Neur_cra, *Neurospora crassa*; Neur_tet, *Neurospora tetrasperma*; Sach_cer, *Saccharomyces cerevisiae*; Metha_ja, *Methanocaldococcus jannaschii*; Baci_sub, *Bacillus subtilis*; Baci_hal, *Bacillus halodurans*.

Thus, the phylogeny of the TS domain clearly revealed a monophyletic origin of the bikont clade. To obtain a better idea of the relative distance between these various species, we represented the ML phylogeny in a three-dimensional space (Fig. 7).

This figure represents a ‘sequence space’. It is the projection in 3D of the highly multidimensional space containing all the possible sequences of the TS domain^46,47^. To assign a position to each sequence in this three dimensional space, non-linear mapping methods are used to offer a configuration of points preserving as much as possible the distances observed between the various sequences in the original space (see Material and Methods). With such methods, axes become arbitrary and every rotation or symmetry is admissible^48^. The meaning of this representation is therefore essentially carried by distance: the distance between two points on the figure tends to display the distance between species. Links between points show the ML phylogenic tree, and thus the figure combines distance-based and ML-based phylogenies.

Such representation can provide additional information compared to the previous one. For example, Toxoplasma gondii, an apicomplexan having a bifunctional DHFR-TS, was positioned far from other apicomplexa but close to organisms such as Leishmania major, an excavate, a situation different from that seen in Fig. 6. This illustrates that two points relatively close in the sequence space are not necessarily close from an evolutionary point of view. For example, Bigelowiella natans and Mus musculus, or Toxoplasma gondii and Saccharomyces cerevisiae may appear close in the sequence space whereas they are not related in the ML tree. In other words, the evolutionary distances (as shown in the ML tree) can be large, despite relative similarities between the sequences. It is also clear that monofunctional (in blue) and bifunctional (in red) branches occupy a different space in this three-dimensional representation, indicating a totally independent evolution of the TS domain after its fusion with DHFR. In addition, the location of the Toxoplasma/Neospora group, far from that of the Plasmodium group in the sequence space could explain the relatively low support value of 0.57 (Fig. 6) calculated for the branch separation leading to Chlamydomonas on one hand and alveolates on the other hand.

### DHPS

**Figure 8:**
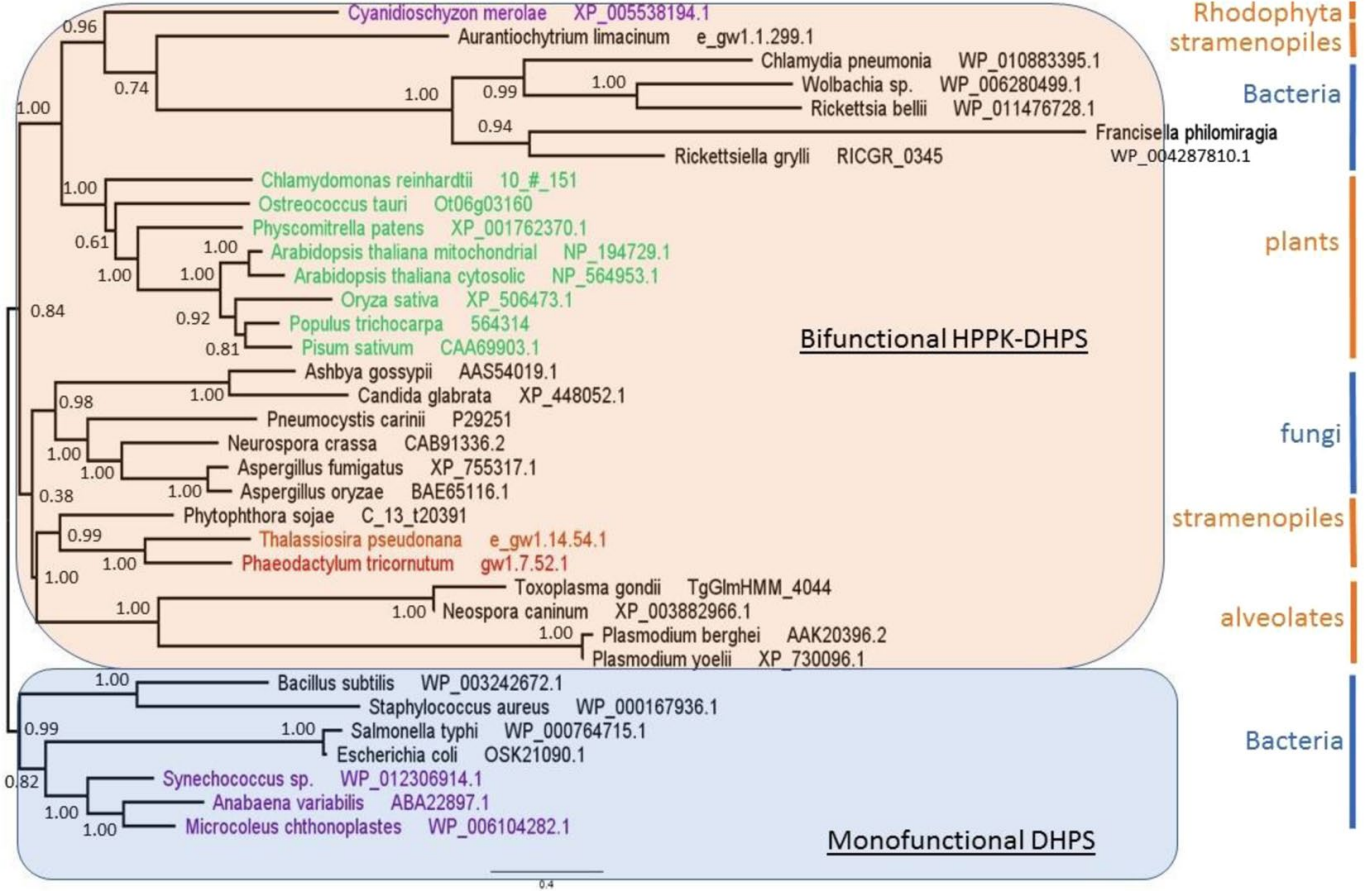
Phylogenetic trees of the protein sequences of the DHPS domain constructed using maximum likelihood method Phyml (see text). The selected model using Bayesian information criterion was LG + G + I with gamma shape parameter estimate = 1.058 and the proportion of invariable site estimate = 0.047. Nodes values represents the Bayesian posterior probabilities branch support. The species colour code corresponds to the type of plastid pigments, as follows: purple, chlorophyll a; green, chlorophyll a and b; and red, chlorophyll a and c.

The maximum likelihood phylogenetic tree obtained for the DHPS domain appears very different from that of TS. In this case also, we identified organisms having a monofunctional DHPS and others having a bifunctional HPPK-DHPS (Fig. 8).

**Figure 9:**
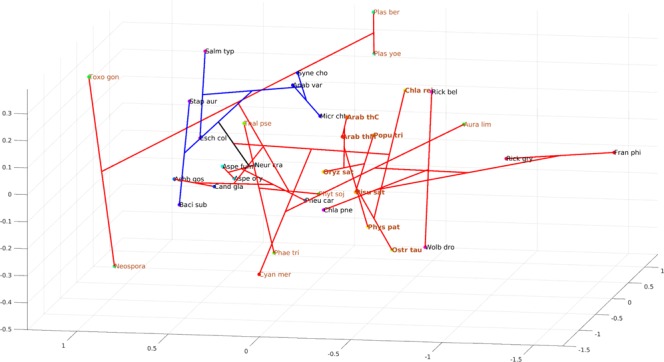
Phylogenetic representation in the protein sequence space for DHPS domains. Non-linear mapping methods are designed to offer a configuration of points in this multidimensional space that is representative of the observed distances. With this method, axes become arbitrary and every rotation or symmetry is admissible. In this figure, the distance between two data points on the figure tends to display the distances between species. Links between points show the ML phylogenic tree presented in the Fig. 8. Blue branches: monofunctional enzyme, red branches: bifunctional enzyme. Brown letters, bikont cluster; brown letters in bold, the green lineage; black letters, unikont cluster. Abbreviations for genus names: Arab_thM, *Arabidopsis thaliana* Mitochondrial; Arab_thC, *Arabidopsis thaliana* Cytosolic; Popu_tri, *Populus trichocarpa*; Pisu_sat, *Pisum sativum*; Oryz_sat, *Oryza sativa*; Phys_pat, *Physcomitrella patens*; Chla_rei, *Chlamydomonas reinhardtii*; Ostr_tau, *Ostreococcus tauri*; Plas_yoe, *Plasmodium yoelii*; Plas_ber, *Plasmodium berghei*; Toxo_gon, *Toxoplasma gondii*; Neos_can, *Neospora caninum*; Cyan_mer, *Cyanidioschyzon merolae*; Phae_tri, *Phaeodactylum tricornutum*; Thal_pse, *Thalassiosira pseudonana*; Aura_lim, *Aurantiochytrium limacinum*; Phyt_soj, *Phytophthora sojae*; Aspe_fum, *Aspergillus fumigatus*; Aspe_ory, *Aspergillus oryzae*; Neur_cra, *Neurospora crassa*; Pneu_car, *Pneumocystis carinii*; Ashb_gos, *Ashbya gossypii*; Cand_gla, *Candida glabrata*; Bige_nat, *Bigelowiella natans*; Rick_gry, *Rickettsiella grylli*; Chla_pne, *Chlamydia pneumonia*; Rick_bel, *Rickettsia bellii*; Wolb_dro, *Wolbachia Drosophila* simulans; Fran_phi, *Francisella philomiragia*; Esch_col*, Escherichia coli*; Salm_typ, *Salmonella typhi*; Baci_sub, *Bacillus subtilis*; Stap_aur, *Staphylococcus aureus*; Syne_cho, *Synechococcus sp*.; Anab_var, *Anabaena variabilis*; Micr_cht, *Microcoleus*.

Animals, which depend on their diet for folate supply, do not possess DHPS activity. Most of the monofunctional DHPS are found in bacteria (unikonts), but some bacteria display also a bifunctional enzyme. From this point of view, it is interesting to note that within gamma proteobacteria there are species displaying monofunctional DHPS (*Escherichia coli*, *Salmonella typhi*) whereas others (*Francisella philomiragia, Ricketsellia grylli*) display a bifunctional HPPK-DHPS. This is also true for the alpha proteobacteria (containing *Rickettsia bellii, Wolbachia*), while beta proteobacteria (not represented here) and firmicutes (*Bacillus subtilis, Staphylococcus aureus*) contain mostly monofunctional DHPS. These data raise the question of the origin of the fusion of the two domains. Likewise, fungi (unikonts) also display a bifunctional HPPK-DHPS, as found in plants (bikonts). Thus, the separation unikonts/bikonts does not apply for this enzyme, suggesting that the evolutionary events leading to the two bifunctional enzymes HPPK-DHPS and DHFR-TS were completely different. The 3D representation (Fig. 9) also shows a different situation compared with the one obtained with the TS domain. In this case, monofunctional and bifunctional DHPS do not occupy a different space. Monofunctional enzymes (blue branches) occupy a small space compared to the bifunctional HPPK-DHPS (red branches) which is widely spread within the entire sequence space. This might suggest more constraint on the monofunctional enzyme, which evolved less than the bifunctional one. It is interesting to note that in Fig. 7 as well as in Fig. 9, the green lineage is always compacted in a rather small space, whereas the apicomplexa are quite widely distributed. This is indicative of a much higher mutational rate in the latter compared with the former.

## Discussion

Folate biosynthesis and metabolism in higher plants have been studied for almost two decades. Despite the wealth of genetic and biochemical evidence, many questions remain to be addressed. Particularly, the regulation and compartmentalisation of folate biosynthesis and metabolism in various plant lineages require scrutiny. Our comparative study encompasses all steps of folate biosynthesis and interconversion of folate species in land plants, as well as green and red algae and reveals novel aspects of folate metabolism that can support further experimental studies in the field and help to design effective biofortification strategies. An earlier comparative genomic analysis of bacterial and plant folate metabolism identified new bacterial GTPCHI and FPGS gene families and predicted a bacterial folate transporter^49^.

Our comparative study revealed that while algae possess single isoforms of the studied genes, plant species tend to have multiple isoforms regulating the same steps in folate metabolism. The multiple isoforms could derive from whole genome duplication or duplication of certain genomic regions, contributing to the development of organelle-specific features of folate metabolism of land plants. Interestingly, three steps of folate biosynthesis, namely ADCS, HPPK-DHPS and DHFS, are catalysed by single isoforms in most analysed land plant species. The restriction of the number of these genes to a single copy in land plant genomes might reflect the necessity for a tight regulation of the abundance of folate intermediates. Being in line with the known inhibition by folate intermediates both at the transcript and the protein levels^50^, this notion suggests that biofortification strategies aiming to enhance folate content should involve elevation of the expression of the downstream genes of folate biosynthesis pathway, namely, ADCS, HPPK-DHPS and DHFS. Such an approach has been already successfully employed to elevate folate content in potato^51^.

It remains to be addressed why certain enzymes of folate biosynthesis are encoded by single isoforms in duplication-prone land plant genomes. Exceptions with two ADCS (in *B. rapa*, *G. max* and *V. vinifera*), HPPK-DHPS (*G. max* and *L. usitatissimum*) and DHFS (*G. max* and *M. truncatula*) are also intriguing. On the one hand, one can ascribe the existence of duplicated isoforms to genetic redundancy inherent to plant genomes. On the other hand, it is possible that the duplicated isoforms might implement a different function or reflect a difference in the regulation of folate biosynthesis. In the future, it will also be important to address whether the duplicated genes encode functional enzymes. Our analysis of conserved amino acids for several folate pathway enzymes, namely, GTPCHI, ADCS, HPPK-DHPS, DHFS and MTHFR, suggests that the isoforms included in our study bear enzymatic activity (Supplemental Figs 32–36). Interestingly, two out the five plant species that bear two copies of ADCS and HPPK-DHPS are naturally rich in folates, namely, *B. rapa* and *G. max*. Apart from ADCS and HPPK-DHPS, *B. rapa* genome bears several copies of GTPCHI. The *B. rapa* case echoes the biofortification strategy applied for potato, where ADCS, GTPCHI and HPPK-DHPS genes have been overexpressed^51^. It is tempting to speculate that such an enhancement approach might prove efficient for species that bear a single copy of the abovementioned genes. However versatile, Arabidopsis is not a flawless model to be used to study regulation of plant metabolism. As highlighted by the study of bifunctional DHFR-TS^40^ and by the present study, Arabidopsis and other species from Brassicaceae, namely, *C. rubella* and *B. rapa* have unique features that are not shared with species from other land plant lineages. Thus, species from Brassicaceae bear enzymatically inactive DHFR-TS homologs that inhibit activity of active homologs, thereby regulating availability of THF^40^. This regulation seems not to be operating in other plant lineages. Brassicaceae members also exceptionally lack cytosolic ADCL isoform (Fig. 4). Moreover, Arabidopsis bears a cytosolic HPPK-DHPS isoform that might be involved in stress response^17^. Interestingly, this feature is unique to Arabidopsis and not shared even with other Brassicaceae species (Supplemental Fig. 5). It is tempting to assume that Arabidopsis might differ from species of other plant lineages in its regulation of folate biosynthesis. Taking this into consideration, drawing parallels from studies using Arabidopsis should be done with caution.

Prediction of subcellular localization allowed to identify novel aspects of folate biosynthesis and metabolism. Although it must be kept in mind that these *in silico* predictions have to be experimentally confirmed, our study suggests that ADCL can localize to the cytosol. The presence of the cytosolic isoform in every analysed land plant species, except species from *Brassicaceae*, might indicate an important function different from that in folate biosynthesis. To date, the cytosolic role of the enzyme remains unknown. It is tempting to speculate that cytosolic ADCL contributes to pABA production that so far has been only reported to occur in plastids in the plant cell. This assumption is in line with studies showing cytosolic localization of certain enzymes operating in the shikimate pathway that provides chorismate for pABA synthesis. If this scenario is operative, there should be a cytosolic conversion of chorismate to aminodeoxychorismate (ADC) similar to that performed by ADCS in plastids; alternatively, ADC can be exported from plastids. As a folate independent, stress-related function for the cytosolic isoform of HPPK-DHPS, an enzyme catalysing the successive step in the folate biosynthesis, has been suggested previously^52^, it is possible that the cytosolic ADCL might also fulfil a role in stress response. Generally speaking, the intracellular compartmentalization of folate biosynthesis established for Arabidopsis (depicted in Fig. 1) seems to be a general feature for land plants. However, the situation might be more complex in algae since the predicted localization of the enzymes appeared more fluctuating from one species to another. For example, there is no putative DHFR activity located in the mitochondria of most algae, and in *K. flaccidum* the entire folate biosynthesis pathway (except for the ADCS activity) could take place in the cytosol.

**Figure 10:**
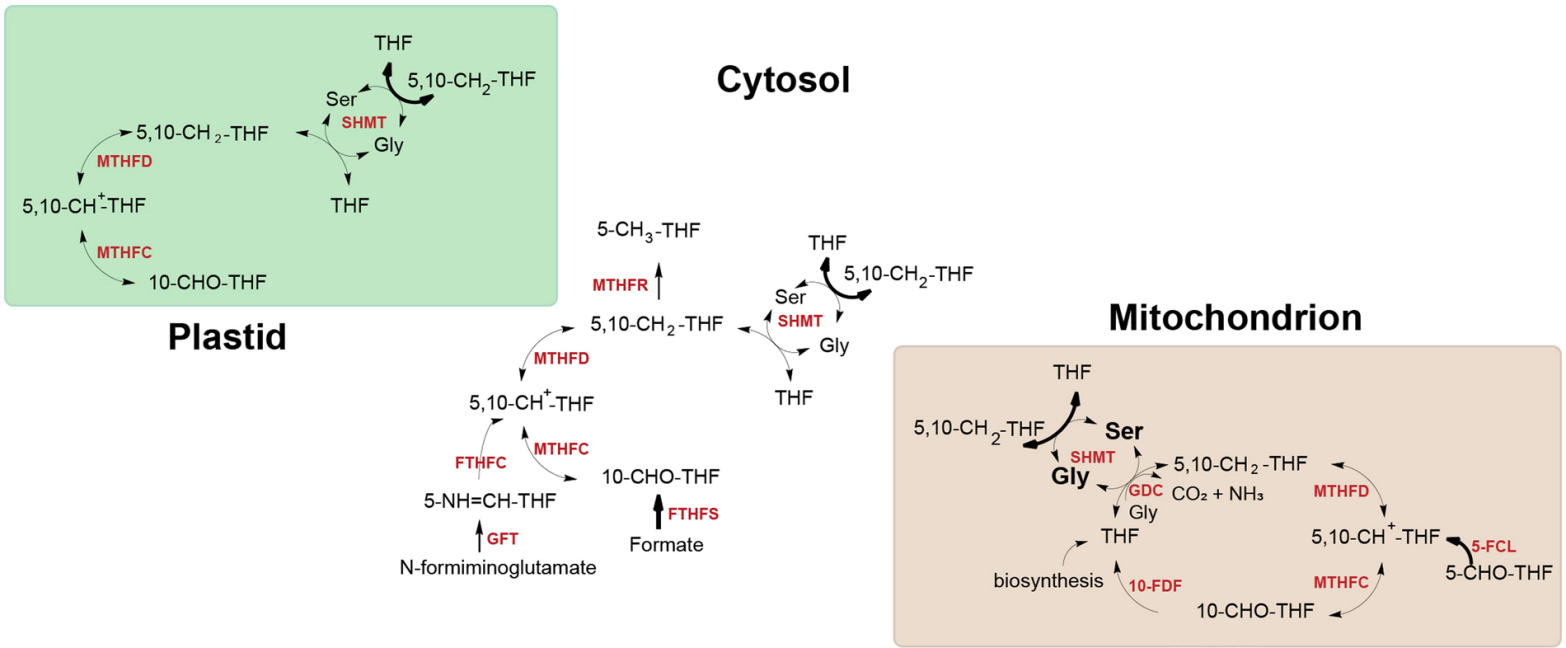
Sources of one-carbon units in the plant cell. Arrows in bold indicate sources of one-carbon units. THF, tetrahydrofolate; 5-CH_3_-THF, 5-methyltetrahydrofolate; 5,10-CH_2_-THF, 5,10-methylenetetrahydrofolate; 5,10-CH^+^-THF, 5,10-methenyltetrahydrofolate; 5-CHO-THF, 5-formyltetrahydrofolate; 10-CHO-THF, 10-formyltetrahydrofolate; Ser, serine; Gly, glycine. SHMT, serine hydroxymethyltransferase; GDC, glycine decarboxylase complex, FTHFC, formiminotetrahydrofolate cyclodeaminase; 5-FCL, 5-formyltetrahydrofolate cycloligase; GFT, glutamate formiminotransferase; FTHFS, 10-CHO-THF synthetase; MTHFD-MTHFC, 5,10- CH_2_-THF dehydrogenase/5,10-CH^+^-THF cyclohydrolase; MTHFR, methylenetetrahydrofolate reductase; 10-FDF, 10-CHO-THF deformylase.

Analysis of subcellular compartmentalization of genes involved in interconversion of folate species suggests that the sources of one-carbon units in plant cells are specific for different subcellular compartments and this specificity is highly conserved across higher plant lineages (Fig. 10). Thus, conversion of 5-CHO-THF, a putative folate storage form, by 5-FCL and the flux of one-carbon units from 10-CHO-THF to 5,10-CH_2_-THF via the sequential action of 10-FDF and GDC are assumed to mainly occur in mitochondria. In the cytosol, one-carbon units appear to be mainly derived from the conversion of Ser to Gly catalysed by SHMT. However, the conversion offormate to 10-CHO-THF mediated by FTHFS and the conversion of N-formiminoglutamate into 5-formimino-THF catalysed by GFT can also serve as a source of one-carbon units. As plastids seem to lack the abovementioned enzymes, they are assumed to mainly obtain their C1-THF derivatives from the activity of SHMT and the transport of metabolically active folate forms from mitochondria and cytosol. Because of the present lack of reliable *in silico* tools for prediction of subcellular localization of algal proteins, localization of folate pathway enzymes in the algae species analysed cannot be unequivocally concluded. Presently, only experimental approaches are able to reveal the localization of enzymatic steps of folate biosynthesis and metabolism.

Our comparative analysis shows that HPPK and DHPS as well as DHFR and TS activities are coupled on a single bifunctional polypeptide across algae and land plant lineages. This is not the case in all organisms, and, as a matter of fact, the fusion between the DHFR and TS genes coincides with the appearance of biflagellate organisms. Indeed, unikonts, with one cilium, show monofunctional enzymes, while bikonts, with two cilia, possess bifunctional enzymes. Our results confirm this well-recognised milestone in the phylogenetic tree of evolution^45^. However, this is not the case for the fusion of the HPPK and DHPS genes which have a completely different evolutionary history compared with DHFR and TS. Indeed, the clade for the bifunctional HPPK-DHPS contains bacteria and fungi (unikonts) as well as plants and alveolates (bikonts). It is interesting to note that all eukaryotes analysed here were grouped in the clade containing the bifunctional enzyme (Fig. 8). Since HPPK-DHPS is a mitochondrial enzyme, it is possible that the bacteria at the origin of the endosymbiotic event that resulted in the advent of mitochondria already contained a bifunctional HPPK-DHPS, and that the gene coding for this enzyme was thereafter transferred to the nucleus. It was proposed that the ancient bacterium likely at the origin of mitochondria belonged to the α-proteobacteria class^53^. Since α-proteobacteria contain species displaying either monofunctional DHPS or bifunctional HPPK-DHPS such as those presented here (Rickettsia, Wolbachia), it would be interesting to know whích α-proteobacterial lineage provided the mitochondrial ancestor. Several studies hint towards the group of Rickettsiales, but this conclusion is today highly challenged^54,55^.

Furthermore, our study reveals that all analysed ADCS homologs, in addition to GAT and chorismate binding domains, possess an anthranilate synthase (AS) domain. Anthranilate synthase (AS) catalyses the first step in the biosynthesis of tryptophan from chorismate^56^. It is thus tempting to speculate that ADCS of algae and higher plant species are capable of anthranilate synthesis. If the AS domain of ADCS is functional, the protein might play a role in tryptophan synthesis that is known to occur in plastids of higher plants^57^ and subsequently contribute to auxin biosynthesis, known to use Trp as a major precursor^58^. Alternatively, it may play a regulatory role therein. A possible contribution of ADCS to auxin synthesis might link the folate and auxin pathways. Interestingly, folate has previously been reported to influence auxin distribution during seedling development, together with sucrose^59^. Furthermore, it was recently shown that root gravitropism is regulated by a crosstalk between PABA (product of the ADCS + ADCL reaction), ethylene and auxin in Arabidopsis^60^.

In several higher plant species some folate pathway enzymes, namely, ADCS, HPPK-DHPS, plastidial MTHFD-MTHFC and GFT, bear additional functional domains. The ACT domain residing on the polypeptide chain of bifunctional MTHFD-MTHFC of *L. usitatissimum* is often found in proteins regulated by amino acids. It is tempting to speculate that this domain might be involved in regulation of the protein by Ser and Gly abundance. However, the domain is absent in all other analysed species, even in the closest relatives of *L. usitatissimum*. Apparently, this domain appeared in MTHFD-MTHFC as a result of a random insertion during genome rearrangement of *L. usitatissimum*. Presence of additional domains in ADCS, HPPK-DHPS and GFT cannot be immediately linked to a specific function. Moreover, the rare occurrence of the additional domains suggests that they occurred in result of random insertions.

In conclusion, the present comparative study revealed novel features of folate metabolism and emphasized the need for further understanding of its regulation in different plant species. Our study suggests that the subcellular localization of folate biosynthesis might be different between algae and land plants, indicating that the localization established in Arabidopsis might not be an absolute physiological requirement. Our phylogenetic analysis implies that duplication of genes controlling certain steps in the folate biosynthesis pathway might have served as a way to increase folate production in certain species. Consequently, the information on the gene numbers could be informative for planning biofortification strategies for related species. The analyses of DHFR/TS and HPPK/DHPS (Figs 6–9) suggest that different steps in the biosynthetic pathway might have a unique evolutionary history.

Once experimentally verified, our findings will be useful for development of species-specific biofortification strategies and better understanding of roles of folate metabolism in plant physiology.

## Materials and Methods

### Identification of putative orthologs and protein analysis

To identify putative orthologs of folate pathway genes, full-length protein sequences from Arabidopsis thaliana were used as a query to run a blastp with E-value ≤ 1e-10 cutoff ^37^. To infer putative orthology the selected protein sequences were scrutinized for the presence of functional protein domains using the Pfam database^38^ and ScanProsite^61^. The conserved motifs were identified using the MEME suite^62^. Analysis of conserved amino acids was conducted using BLAST Conserved Domain Database tool^63^.

### Phylogenetic analysis

Full-length amino acid sequences were aligned and manually corrected and edited in MEGA 7^64^ using MUSCLE software^65^. The phylogenetic relationship was inferred using the maximum likelihood method implemented in RaxML^66^ using WAG + G + I model that was selected by ProtTest^67^. The maximum likelihood tree was evaluated with 1000 bootstrap replicates. Phylogenetic trees were visualized using FigTree. The method described above was used to build phylogenetic trees for Figs 3–5 and Supplemental Figs 3, 5, 7, 9, 12, 14–16, 18, 20–22, 26, 28, 30.

For the comparison of TS and DHPS domains from plants and other organisms, amino acid sequences were retrieved from NCBI resources, JGI genome portal or specific databases such as, plasmoDB and TAIR. Phylogenetic studies of the bifunctional enzymes DHFR-TS and HPPK-DHPS were performed with the largest domain in each case, i.e. TS and DHPS which have been identified using Conserved Domain Search Service v3.15^63^ Parameters: Expect Value threshold = 0.01, Composition based statistics adjustment. Amino acid domains sequences were aligned in an iterative process using ClustalX software^68^. During the process, alignments were curated using both ClustalX 2.0 and Jalview 2^69^. The phylogenetic relationship was inferred using the maximum likelihood method PhyML^70^ with the Smart Model Selection^71^ under the Bayesian information criterion (BIC)^72^. The maximum likelihood tree was evaluated with aBayes^73^ and phylogenetic trees were visualized using FigTree.

The phylogenetic trees obtained from the maximum likelihood method PhyML were represented in the protein sequence space, where each residue in the protein is represented by a dimension with 20 possible positions along that axis, corresponding to the possible amino acids^47,74,75^. This space is high-dimensional since its dimension is 20 to the power of the number of residues in the sequence. Nevertheless, it can be represented in a 3D space by using Multidimensional scaling methods which consider the distances between data in the original high dimensional space and associate a configuration of points in a lower dimensional space (the so called representation space, which is here a 3D Euclidean space)^76,77^. Obviously, distances cannot be always preserved and distortions appear^78^. Most often, the preservation of short distances is favored so as to preserve local properties. Consequently, axes have no specific meaning and maps must be considered as invariant by translation, rotation and symmetry. The information in maps is carried by distances between points. Here we used the Sammon’s mapping^79^. It should be emphasized that multidimensional scaling offers an intuitive representation of the original data structure (the similarity between sequences). Distances between sequences were computed using the PAM250 matrix and the Phylip prodist program^80^. The method described above was used to build phylogenetic trees for Figs 6 and 8.

### Protein subcellular localization

Subcellular localization of analysed proteins was predicted using TargetP^81^, SherLoc2^82^, MultiLoc^83^, PredAlgo^84^. Double localizations originated from predictions by different tools.

## Supplementary information

Supplemental data

## References

[1] Chistoserdova L, Vorholt JA, Thauer RK, Lidstrom ME (1998). C1 transfer enzymes and coenzymes linking methylotrophic bacteria and methanogenic Archaea. Science.

[2] Edman JC, Goldstein AL, Erbe JG (1993). Para‐aminobenzoate synthase gene of Saccharomyces cerevisiae encodes a bifunctional enzyme. Yeast.

[3] James TY (2002). The pab1 gene of Coprinus cinereus encodes a bifunctional protein for paraaminobenzoic acid (PABA) synthesis: implications for the evolution of fused PABA synthases. Journal of Basic Microbiology.

[4] Triglia T, Cowman AF (1999). Plasmodium falciparum: a homologue of p-aminobenzoic acid synthetase. Experimental Parasitology.

[5] Basset GJ (2004). Folate synthesis in plants: the p-aminobenzoate branch is initiated by a bifunctional PabAPabB protein that is targeted to plastids. Proceedings of the National Academy of Sciences of the United States of America.

[6] Camara D, Richefeu-Contesto C, Gambonnet B, Dumas R, Rébeillé F (2011). The synthesis of pABA: Coupling between the glutamine amidotransferase and aminodeoxychorismate synthase domains of the bifunctional aminodeoxychorismate synthase from Arabidopsis thaliana. Archives of Biochemistry and Biophysics.

[7] Basset GJ (2004). Folate synthesis in plants: the last step of the p‐aminobenzoate branch is catalyzed by a plastidial aminodeoxychorismate lyase. The Plant Journal.

[8] Green JM, Merkel WK, Nichols BP (1992). Characterization and sequence of Escherichia coli pabC, the gene encoding aminodeoxychorismate lyase, a pyridoxal phosphate-containing enzyme. Journal of Bacteriology.

[9] Auerbach G (2000). Zinc plays a key role in human and bacterial GTP cyclohydrolase I. Proceedings of the National Academy of Sciences.

[10] Nar H (1995). Active site topology and reaction mechanism of GTP cyclohydrolase I. Proceedings of the National Academy of Sciences.

[11] Basset G (2002). Folate synthesis in plants: the first step of the pterin branch is mediated by a unique bimodular GTP cyclohydrolase I. Proceedings of the National Academy of Sciences.

[12] Klaus SM (2005). A nudix enzyme removes pyrophosphate from dihydroneopterin triphosphate in the folate synthesis pathway of bacteria and plants. Journal of Biological Chemistry.

[13] Suzuki Y, Brown GM (1974). The biosynthesis of folic acid XII. Purification and properties of dihydroneopterin triphosphate pyrophosphohydrolase. Journal of Biological Chemistry.

[14] Goyer A (2004). Folate biosynthesis in higher plants. cDNA cloning, heterologous expression, and characterization of dihydroneopterin aldolases. Plant Physiology.

[15] Rébeillé F, Macherel D, Mouillon JM, Garin J, Douce R (1997). Folate biosynthesis in higher plants: purification and molecular cloning of a bifunctional 6‐hydroxymethyl‐7,8‐dihydropterin pyrophosphokinase/7,8‐dihydropteroate synthase localized in mitochondria. The EMBO Journal.

[16] Mouillon J-M, Ravanel S, Douce R, Rébeillé F (2002). Folate synthesis in higher-plant mitochondria: coupling between the dihydropterin pyrophosphokinase and the dihydropteroate synthase activities. Biochemical Journal.

[17] Storozhenko S (2007). Cytosolic Hydroxymethyldihydropterin Pyrophosphokinase/Dihydropteroate Synthase from Arabidopsis thaliana A Specific Role in Early Development and Stress Response. Journal of Biological Chemistry.

[18] Gillies, S. A., McIntosh, S. R. & Henry, R. J. A cereal crop with enhanced folate: Rice transgenic for wheat HPPK/DHPS (2008).

[19] Luo M, Orsi R, Patrucco E, Pancaldi S, Cella R (1997). Multiple transcription start sites of the carrot dihydrofolate reductase-thymidylate synthase gene, and sub-cellular localization of the bifunctional protein. Plant Molecular Biology.

[20] Neuburger M, Rébeillé F, Jourdain A, Nakamura S, Douce R (1996). Mitochondria are a major site for folate and thymidylate synthesis in plants. Journal of Biological Chemistry.

[21] Cox K, Robertson D, Fites R (1999). Mapping and expression of a bifunctional thymidylate synthase, dihydrofolate reductase gene from maize. Plant Molecular Biology.

[22] Lazar G, Zhang H, Goodman HM (1993). The origin of the bifunctional dihydrofolate reductasethymidylate synthase isogenes of Arabidopsis thaliana. The Plant Journal.

[23] Akhtar TA (2008). Tomato γ-glutamylhydrolases: expression, characterization, and evidence for heterodimer formation. Plant Physiology.

[24] Akhtar TA (2010). A central role for gamma‐glutamyl hydrolases in plant folate homeostasis. The Plant Journal.

[25] Orsomando G (2005). Plant γ-Glutamyl Hydrolases and Folate Polyglutamates Characterization, Compartmentation, and Co-Occurrence in Vacuoles. Journal of Biological Chemistry.

[26] Ravanel, S., Douce, R. & Rebeille, F. In *Advances in Botanical Researc*h Vol. 59, 67–106 (Elsevier, 2011).

[27] Hanson AD, Roje S (2001). One-carbon metabolism in higher plants. Annual Review of Plant Biology.

[28] Mouillon JM (1999). Glycine and serine catabolism in non‐photosynthetic higher plant cells: their role in C1 metabolism. The Plant Journal.

[29] Besson V, Neuburger M, Rébeillé F, Douce R (1995). Evidence for three serine hydroxymethyltransferases in green. Plant Physiol. Biochem.

[30] Kirk CD, Imeson HC, Zheng L-L, Cossins EA (1994). The affinity of pea cotyledon 10-formyltetrahydrofolate synthetase for polyglutamate substrates. Phytochemistry.

[31] Kirk CD, Chen L, Imeson HC, Cossins EA (1995). A 5, 10-methylenetetrahydrofolate dehydrogenase: 5, 10-methenyltetrahydrofolate cyclohydrolase protein from Pisum sativum. Phytochemistry.

[32] Collakova E (2008). Arabidopsis 10-formyl tetrahydrofolate deformylases are essential for photorespiration. The Plant Cell.

[33] Stover P, Schirch V (1993). The metabolic role of leucovorin. Trends in Biochemical Sciences.

[34] Jeanguenin L (2010). Moonlighting glutamate formiminotransferases can functionally replace 5-formyltetrahydrofolate cycloligase. Journal of Biological Chemistry.

[35] Guo, W. *et al*. In *ICPM conference Dalian*, *China* (2017).

[36] Strobbe S, Van Der Straeten D (2017). Folate biofortification in food crops. Current Opinion in Biotechnology.

[37] Altschul SF, Gish W, Miller W, Myers EW, Lipman DJ (1990). Basic local alignment search tool. Journal of Molecular Biology.

[38] Bateman A (2002). The Pfam protein families database. Nucleic Acids Research.

[39] Ravanel S (2001). Tetrahydrofolate biosynthesis in plants: molecular and functional characterization of dihydrofolate synthetase and three isoforms of folylpolyglutamate synthetase in Arabidopsis thaliana. Proceedings of the National Academy of Sciences.

[40] Gorelova V (2017). Dihydrofolate reductase/thymidylate synthase fine-tunes the folate status and controls redox homeostasis in plants. The Plant Cell.

[41] Mehrshahi P (2010). Functional analysis of folate polyglutamylation and its essential role in plant metabolism and development. The Plant Journal.

[42] Oliver DJ (1994). The glycine decarboxylase complex from plant mitochondria. Annual Review of Plant Biology.

[43] Roje S, Janave MT, Ziemak MJ, Hanson AD (2002). Cloning and characterization of mitochondrial 5-formyltetrahydrofolate cycloligase from higher plants. Journal of Biological Chemistry.

[44] Isegawa Y, Watanabe F, Kitaoka S, Nakano Y (1993). Subcellular distribution of cobalamin-dependent methionine synthase in Euglena gracilis Z. Phytochemistry.

[45] Stechmann A, Cavalier-Smith T (2002). Rooting the eukaryote tree by using a derived gene fusion. Science.

[46] Bastien O (2004). Analysis of the compositional biases in Plasmodium falciparum genome and proteome using Arabidopsis thaliana as a reference. Gene.

[47] Bastien O, Ortet P, Roy S, Maréchal E (2005). A configuration space of homologous proteins conserving mutual information and allowing a phylogeny inference based on pair-wise Z-score probabilities. BMC Bioinformatics.

[48] Degret, F. & Lespinats, S. In *MATEC Web of Conferences*. 10002 (EDP Sciences) (2018).

[49] de Crécy-Lagard V, El Yacoubi B, de la Garza RD, Noiriel A, Hanson AD (2007). Comparative genomics of bacterial and plant folate synthesis and salvage: predictions and validations. BMC Genomics.

[50] Gorelova, V., Ambach, L., Rébeillé, F., Stove, C. & Van Der Straeten, D. Folates in Plants: Research Advances and Progress in Crop Biofortification. *Frontiers in Chemistry* **5** (2017). 10.3389/fchem.2017.00021 PMC5372827 28424769

[51] De Lepeleire J (2018). Folate biofortification of potato by tuber-specific expression of four folate biosynthesis genes. Molecular Plant.

[52] Navarrete O (2012). A folate independent role for cytosolic HPPK/DHPS upon stress in Arabidopsis thaliana. Phytochemistry.

[53] Yang D, Oyaizu Y, Oyaizu H, Olsen GJ, Woese CR (1985). Mitochondrial origins. Proceedings of the National Academy of Sciences.

[54] Fitzpatrick DA, Creevey CJ, McInerney JO (2005). Genome phylogenies indicate a meaningful α-proteobacterial phylogeny and support a grouping of the mitochondria with the Rickettsiales. Molecular Biology and Evolution.

[55] Martijn J, Vosseberg J, Guy L, Offre P, Ettema TJ (2018). Deep mitochondrial origin outside the sampled alphaproteobacteria. Nature.

[56] Kawamura M, Keim PS, Goto Y, Zalkin H, Heinrikson RL (1978). Anthranilate synthetase component II from Pseudomonas putida. Covalent structure and identification of the cysteine residue involved in catalysis. Journal of Biological Chemistry.

[57] Radwanski ER, Last RL (1995). Tryptophan biosynthesis and metabolism: biochemical and molecular genetics. The Plant Cell.

[58] Mano Y, Nemoto K (2012). The pathway of auxin biosynthesis in plants. Journal of Experimental Botany.

[59] Stokes ME, Chattopadhyay A, Wilkins O, Nambara E, Campbell MM (2013). Interplay between sucrose and folate modulates auxin signaling in Arabidopsis. Plant Physiology.

[60] Nziengui H (2018). Root gravitropism is regulated by a crosstalk between para-aminobenzoic acid, ethylene, and auxin. Plant Physiology.

[61] Gattiker A, Gasteiger E, Bairoch AM (2002). ScanProsite: a reference implementation of a PROSITE scanning tool. Applied Bioinformatics.

[62] Bailey TL (2009). MEME SUITE: tools for motif discovery and searching. Nucleic Acids Research.

[63] Marchler-Bauer A (2010). CDD: a Conserved Domain Database for the functional annotation of proteins. Nucleic Acids Research.

[64] Kumar S, Stecher G, Tamura K (2016). MEGA7: Molecular Evolutionary Genetics Analysis version 7.0 for bigger datasets. Molecular Biology and Evolution.

[65] Edgar RC (2004). MUSCLE: multiple sequence alignment with high accuracy and high throughput. Nucleic Acids Research.

[66] Stamatakis A (2014). RAxML version 8: a tool for phylogenetic analysis and post-analysis of large phylogenies. Bioinformatics.

[67] Abascal F, Zardoya R, Posada D (2005). ProtTest: selection of best-fit models of protein evolution. Bioinformatics.

[68] Larkin MA (2007). Clustal W and Clustal X version 2.0. Bioinformatics.

[69] Waterhouse AM, Procter JB, Martin DM, Clamp M, Barton GJ (2009). Jalview Version 2—a multiple sequence alignment editor and analysis workbench. Bioinformatics.

[70] Guindon S (2010). New algorithms and methods to estimate maximum-likelihood phylogenies: assessing the performance of PhyML 3.0. Systematic Biology.

[71] Lefort, V., Longueville, J.-E. & Gascuel, O. SMS: Smart Model Selection in PhyML. *Molecular Biology and Evolution*, msx149 (2017). 10.1093/molbev/msx149 PMC5850602 28472384

[72] Neath AA, Cavanaugh JE (2012). The Bayesian information criterion: background, derivation, and applications. Wiley Interdisciplinary Reviews: Computational Statistics.

[73] Anisimova M, Gil M, Dufayard J-F, Dessimoz C, Gascuel O (2011). Survey of branch support methods demonstrates accuracy, power, and robustness of fast likelihood-based approximation schemes. Systematic Biology.

[74] Bornberg-Bauer E, Chan HS (1999). Modeling evolutionary landscapes: mutational stability, topology, and superfunnels in sequence space. Proceedings of the National Academy of Sciences.

[75] Dryden DT, Thomson AR, White JH (2008). How much of protein sequence space has been explored by life on Earth?. Journal of The Royal Society Interface.

[76] France SL, Carroll JD (2011). Two-way multidimensional scaling: A review. IEEE Transactions on Systems, Man, and Cybernetics, Part C (Applications and Reviews).

[77] Lee, J. A. & Verleysen, M. *Nonlinear Dimensionality Reduction*. (Springer Science & Business Media, 2007).

[78] Lespinats, S. & Aupetit, M. In *Computer Graphics Forum*. 113–125 (Wiley Online Library, 2010).

[79] Sammon JW (1969). A nonlinear mapping for data structure analysis. IEEE Transactions on Computers.

[80] Felsenstein J (1981). Evolutionary trees from DNA sequences: a maximum likelihood approach. Journal of Molecular Evolution.

[81] Emanuelsson O, Brunak S, Von Heijne G, Nielsen H (2007). Locating proteins in the cell using TargetP, SignalP and related tools. Nature Protocols.

[82] Briesemeister S (2009). SherLoc2: a high-accuracy hybrid method for predicting subcellular localization of proteins. Journal of Proteome Research.

[83] Blum T, Briesemeister S, Kohlbacher O (2009). MultiLoc2: integrating phylogeny and Gene Ontology terms improves subcellular protein localization prediction. BMC Bioinformatics.

[84] Tardif M (2012). PredAlgo: a new subcellular localization prediction tool dedicated to green algae. Molecular Biology and Evolution.

