# Supplementary material for: Evolution of folate biosynthesis and metabolism across algae and land plant lineages
Source: Sci Rep. 2019 Apr 5;9:5731. doi: 10.1038/s41598-019-42146-5 (PMC6451014; doi:10.1038/s41598-019-42146-5)
Supplement: Supplementary file 1 — Supplemental data [file 41598_2019_42146_MOESM1_ESM.docx]

**Supplementary Information**

**Evolution of folate biosynthesis and metabolism across algae and land plant lineages**

**Gorelova, V., Bastien, O., De Clerck, O., Lespinats, S., Rébeillé, F., Van Der Straeten, D.**


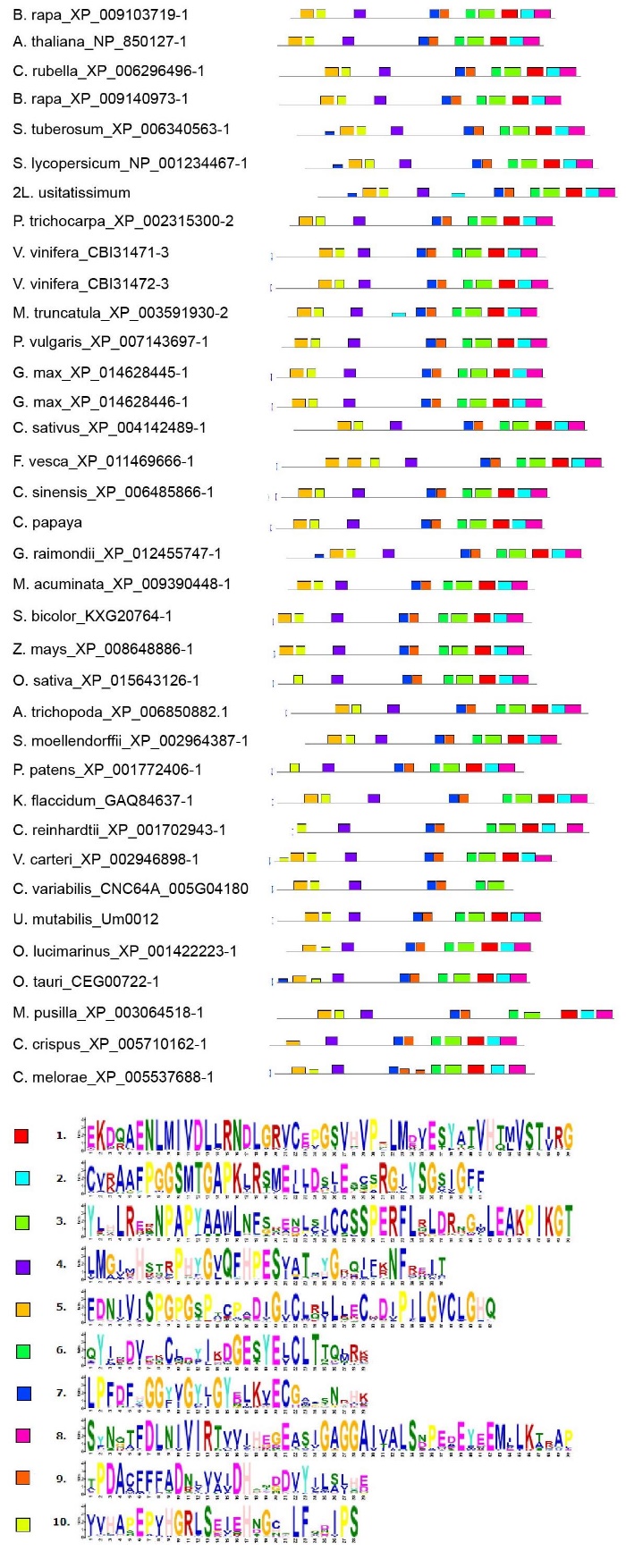


**Supplemental Figure 1.** Conserved protein motif pattern of ADCS.

Species names are followed by protein identifiers, blocks represent conserved protein motifs. Logos visualize motifs. The height of a letter indicates its relative frequency at the given position.


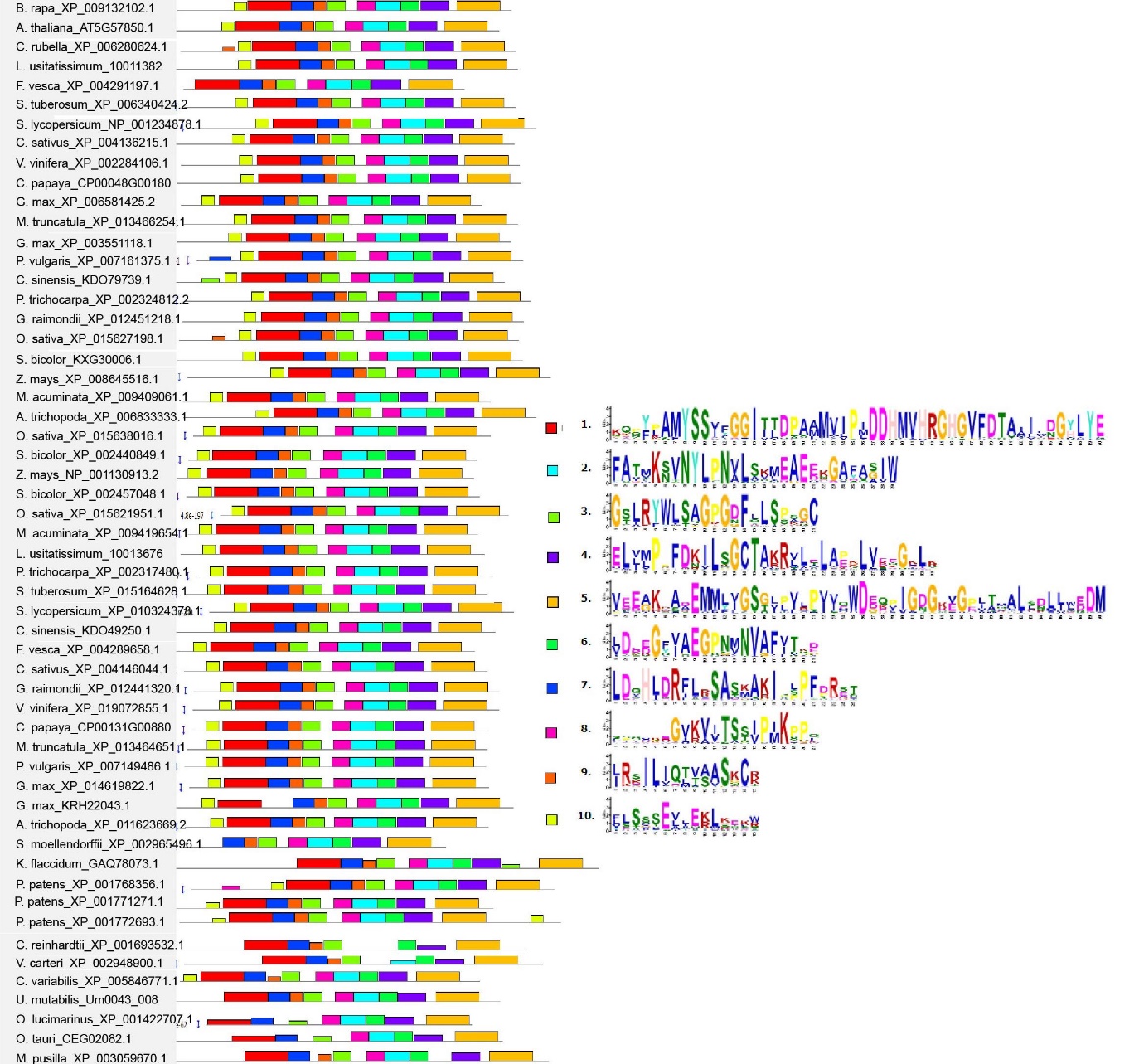


**Supplemental Figure 2.** Conserved protein motif pattern of ADCL.

Species names are followed by protein identifiers, blocks represent conserved protein motifs. Logos visualize motifs. The height of a letter indicates its relative frequency at the given position.


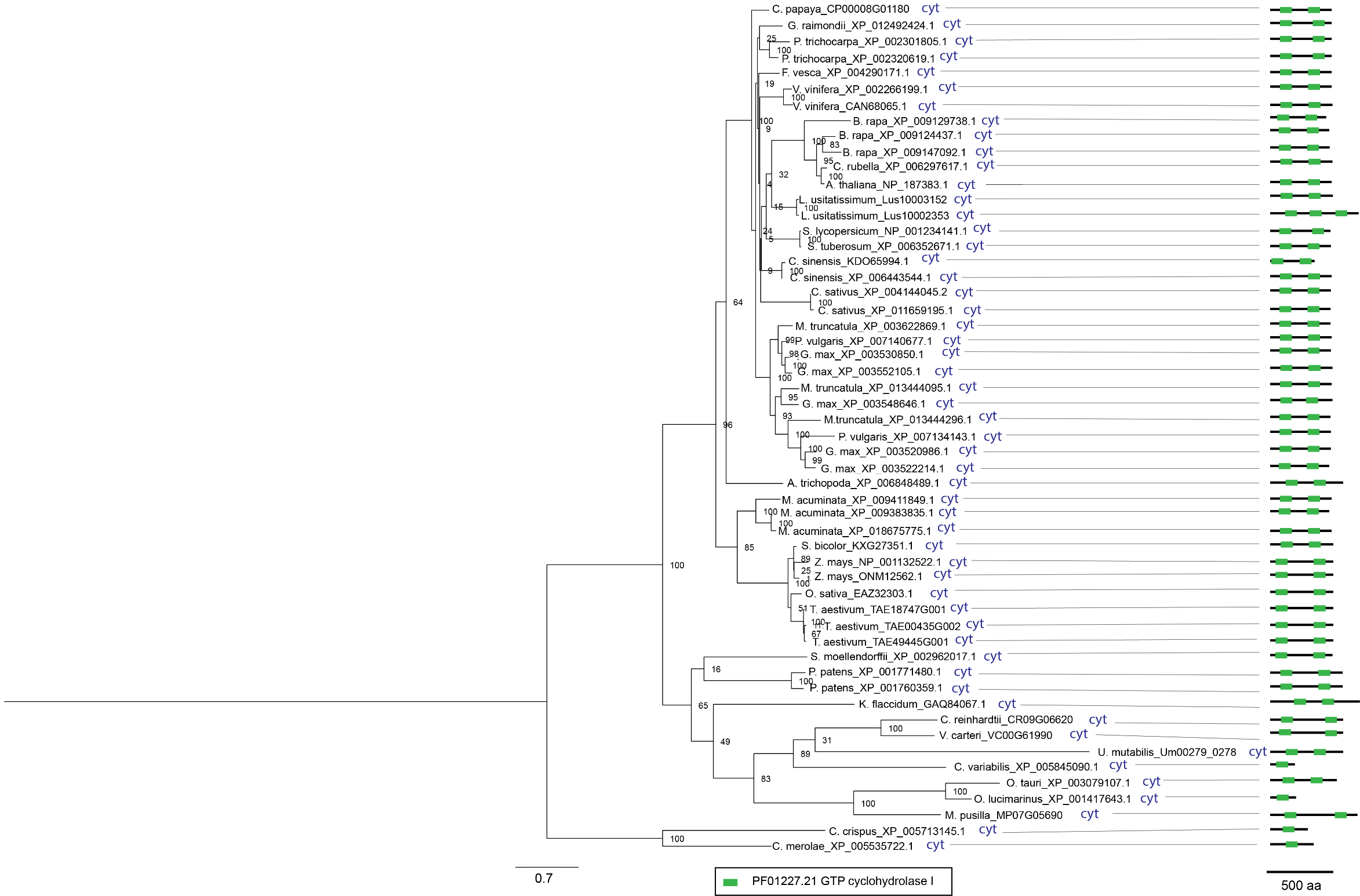


**Supplemental Figure 3.** Phylogenetic analysis, subcellular localization and domain composition of GTPCHI proteins.

Species names are followed by protein identifiers. The bar indicates the mean distance of 0.7 changes per amino acid residue. The numbers at the branching points indicate the percentage of times that each branch topology was found during bootstrap analysis (n=1000). Schemes on the right represent domain organisation of analysed proteins (color boxes represent functional domains, lengths of black lines correspond to lengths of proteins. The scale bar below shows protein containing 500 amino acids). The box contains predicted functional domains. Cyt, cytosolic localization.


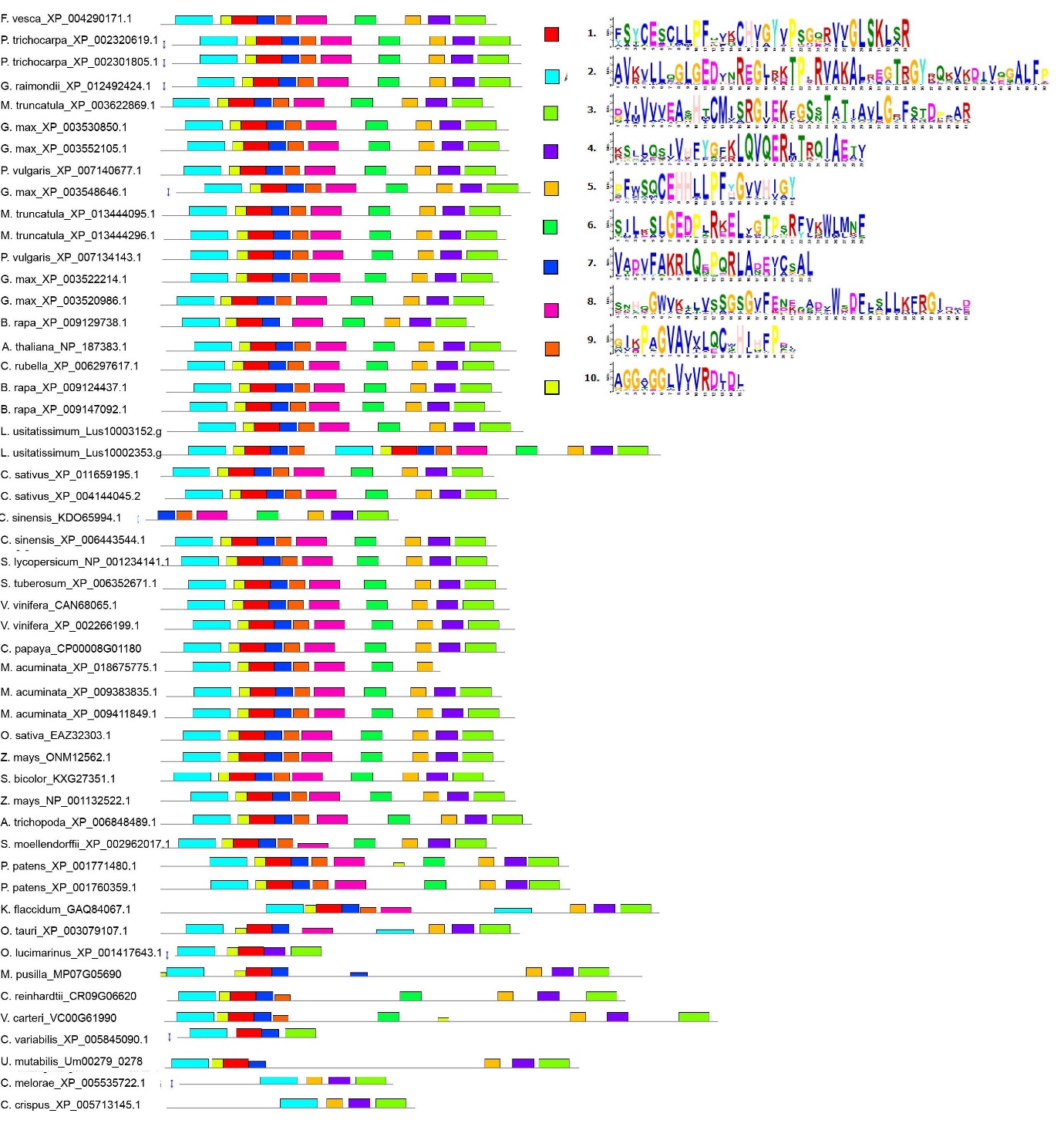


**Supplemental Figure 4.** Conserved protein motif pattern of GTPCHI.

Species names are followed by protein identifiers, blocks represent conserved protein motifs. Logos visualize motifs. The height of a letter indicates its relative frequency at the given position.


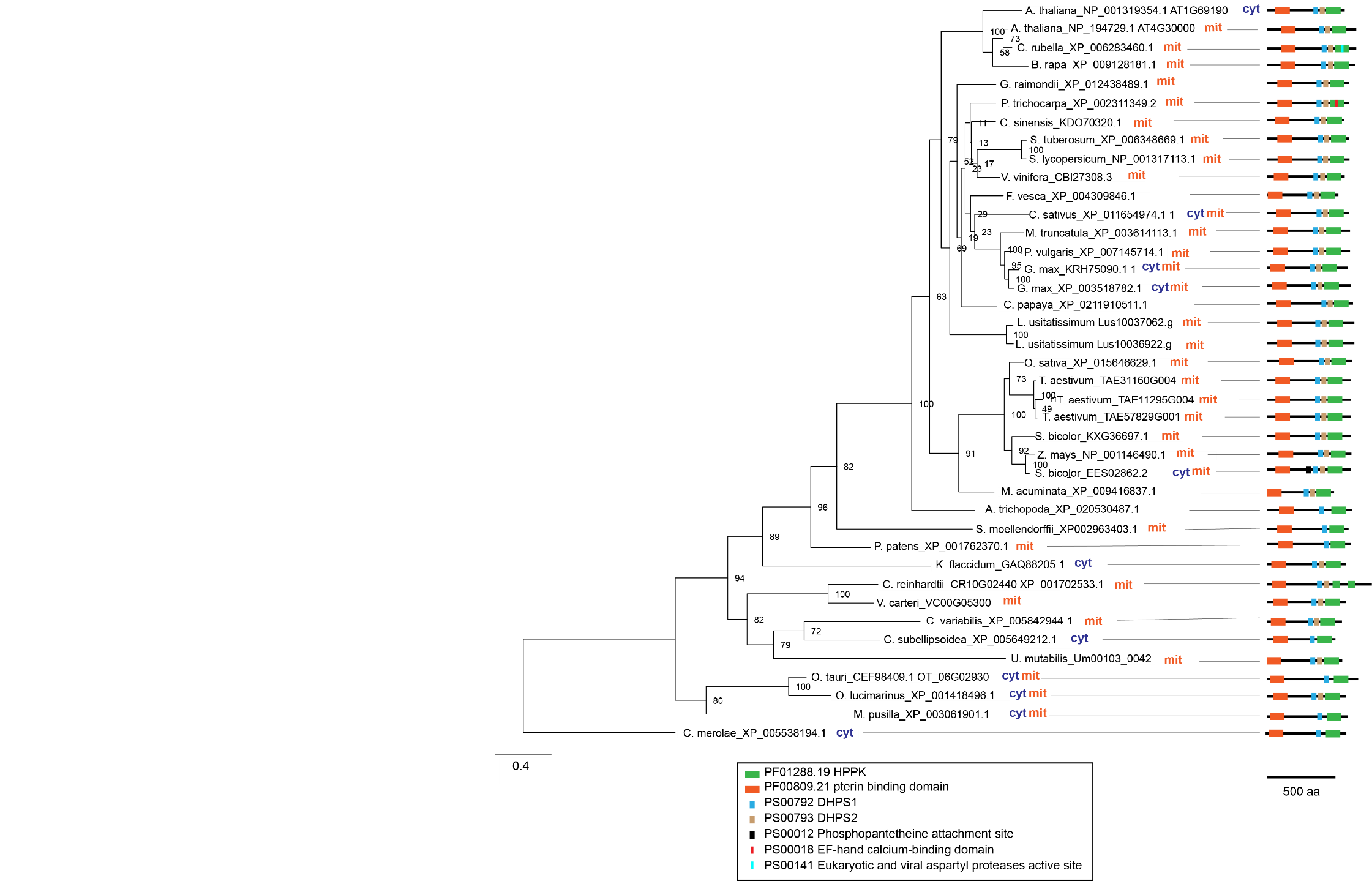


**Supplemental Figure 5.** Phylogenetic analysis, subcellular localization and domain composition of HPPK-DHPS proteins.

Species names are followed by protein identifiers. The bar indicates the mean distance of 0.4 changes per amino acid residue. The numbers at the branching points indicate the percentage of times that each branch topology was found during bootstrap analysis (n=1000). Schemes on the right represent domain organisation of analysed proteins (color boxes represent functional domains, lengths of black lines correspond to lengths of proteins. The scale bar below shows protein containing 500 amino acids). The box contains predicted functional domains. Cyt, cytosolic localization; mit, mitochondrial localization, amb, ambiguous localization.


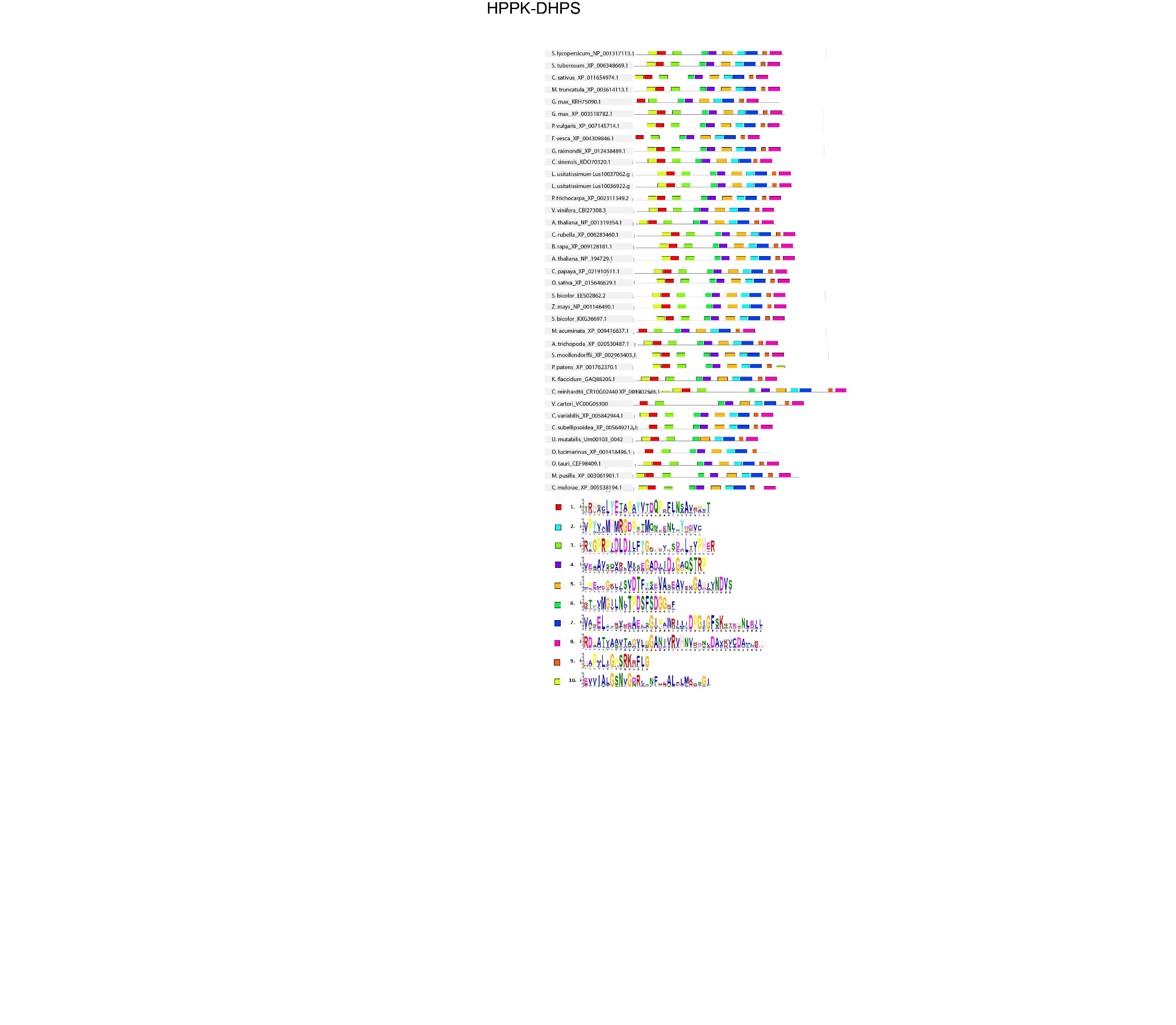


**Supplemental Figure 6.** Conserved protein motif pattern of HPPK-DHPS.

Species names are followed by protein identifiers, blocks represent conserved protein motifs. Logos visualize motifs. The height of a letter indicates its relative frequency at the given position.


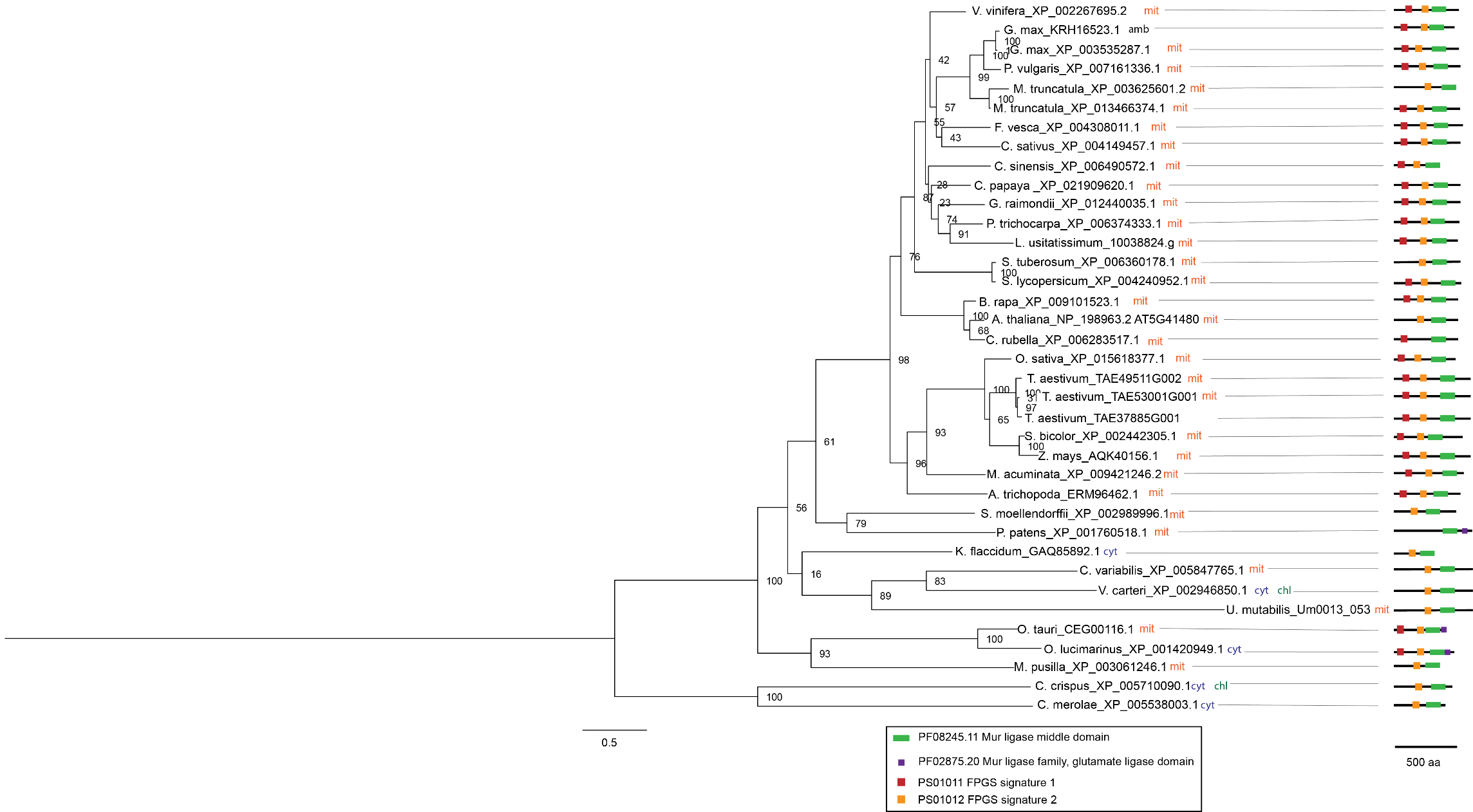


**Supplemental Figure 7.** Phylogenetic analysis, subcellular localization and domain composition of DHFS proteins.

Species names are followed by protein identifiers. The bar indicates the mean distance of 0.5 changes per amino acid residue. The numbers at the branching points indicate the percentage of times that each branch topology was found during bootstrap analysis (n=1000). Schemes on the right represent domain organisation of analysed proteins (color boxes represent functional domains, lengths of black lines correspond to lengths of proteins. The scale bar below shows protein containing 500 amino acids). The box contains predicted functional domains. Cyt, cytosolic localization; chl, plastidial localization; mit, mitochondrial localization.


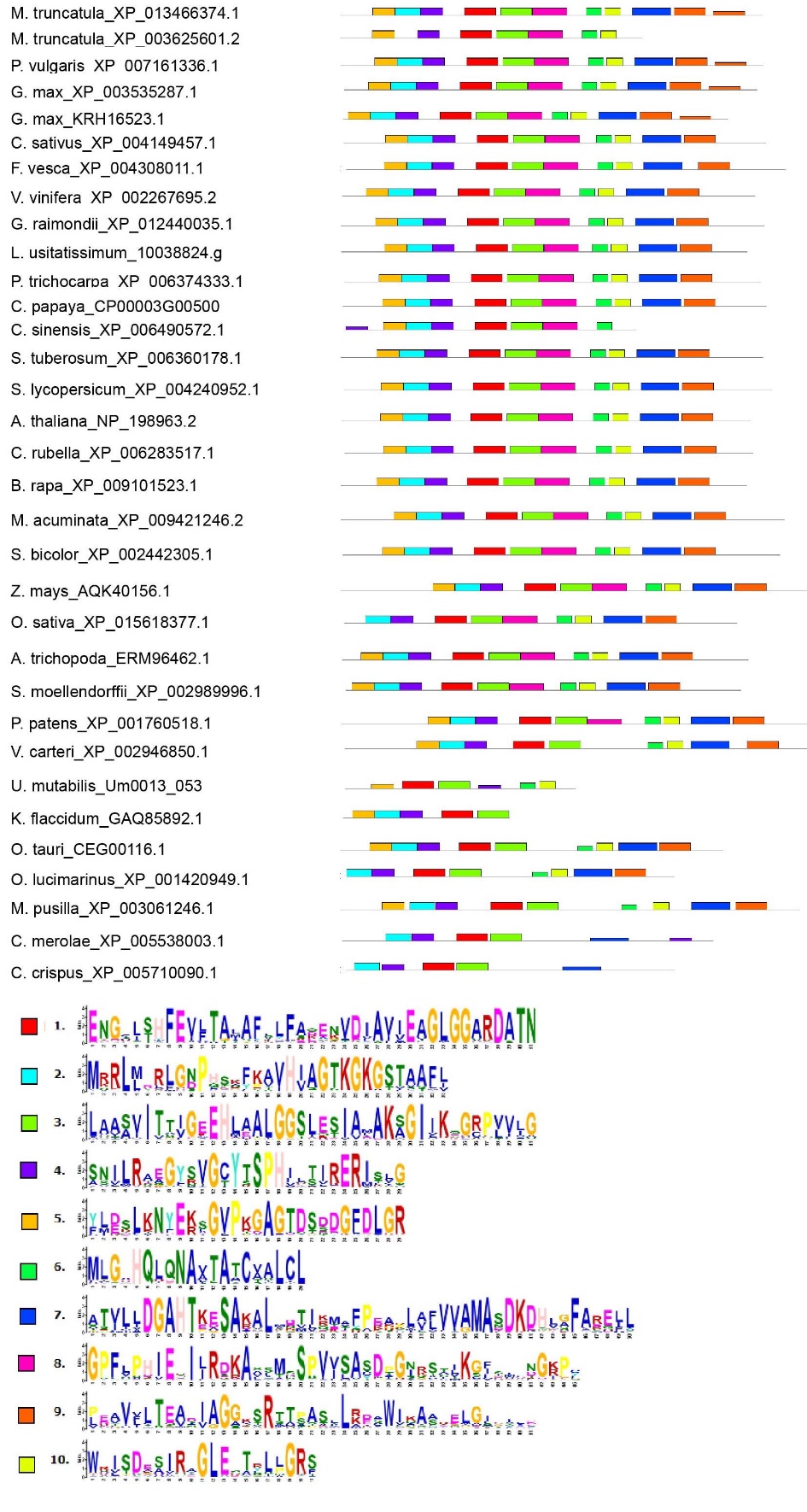


**Supplemental Figure 8.** Conserved protein motif pattern of DHFS.

Species names are followed by protein identifiers, blocks represent conserved protein motifs. Logos visualize motifs. The height of a letter indicates its relative frequency at the given position.


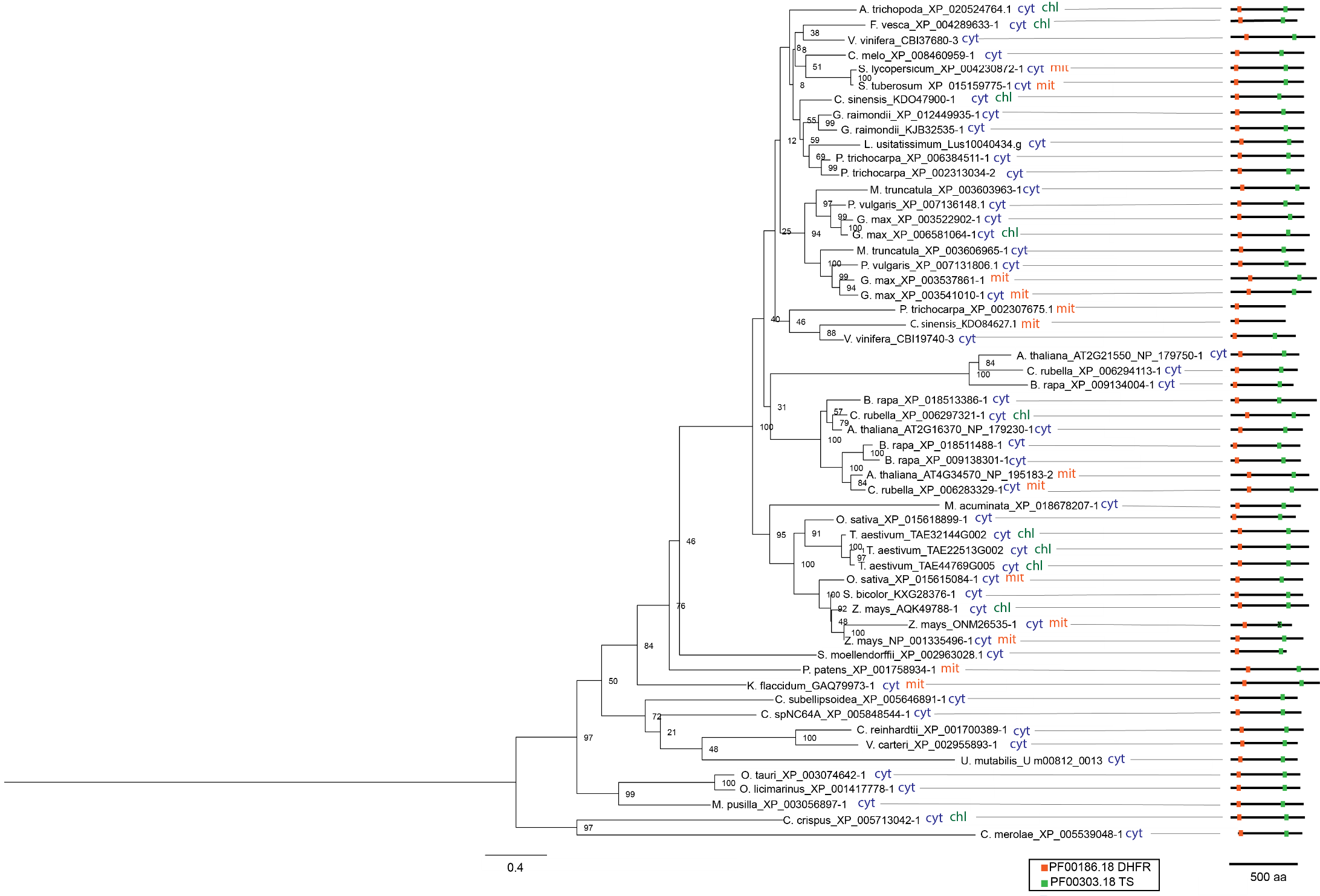


**Supplemental Figure 9.** Phylogenetic analysis, subcellular localization and domain composition of DHFR proteins.

Species names are followed by protein identifiers. The bar indicates the mean distance of 0.4 changes per amino acid residue. The numbers at the branching points indicate the percentage of times that each branch topology was found during bootstrap analysis (n=1000). Schemes on the right represent domain organisation of analysed proteins (color boxes represent functional domains, lengths of black lines correspond to lengths of proteins. The scale bar below shows protein containing 500 amino acids). The box contains predicted functional domains. Cyt, cytosolic localization; chl, plastidial localization; mit, mitochondrial localization.


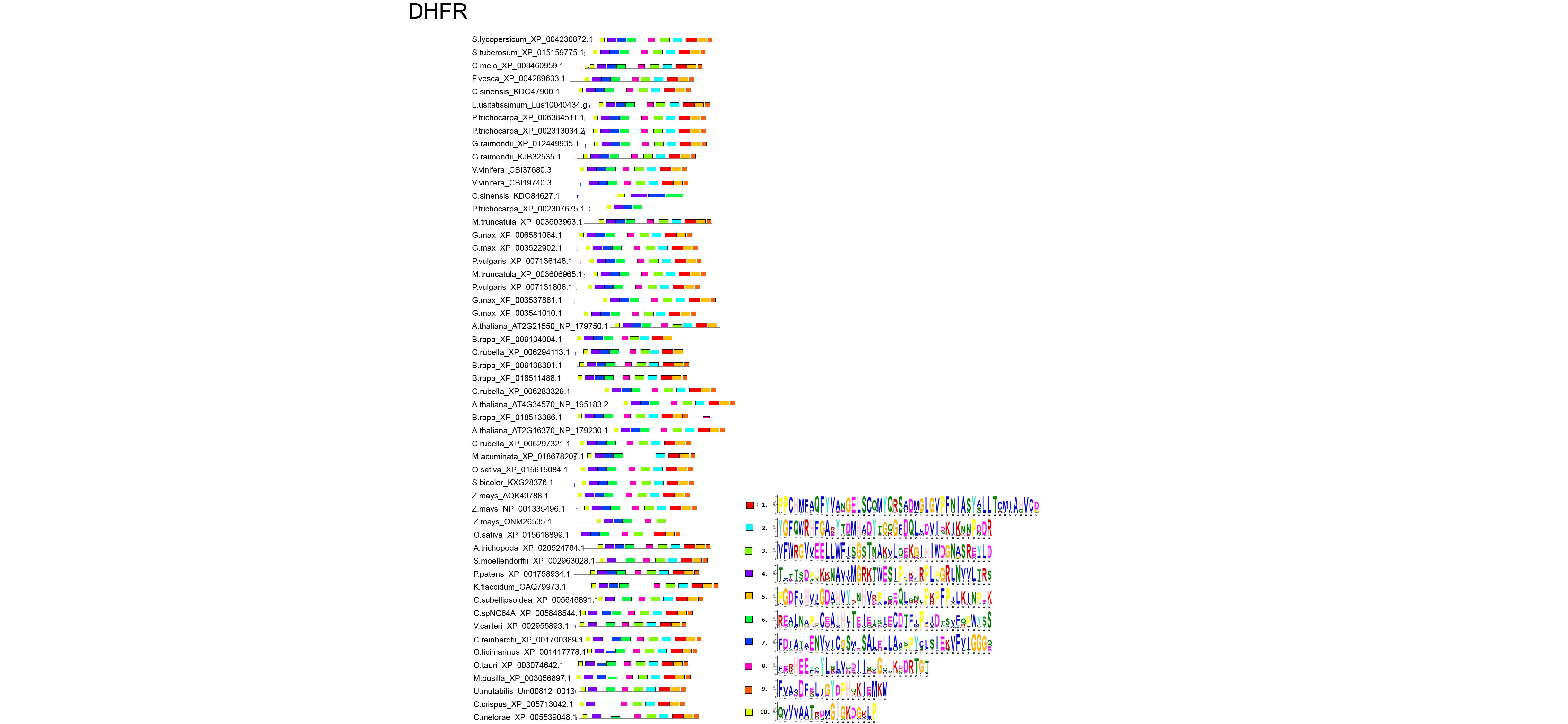


**Supplemental Figure 10.** Conserved protein motif pattern of DHFR.

Species names are followed by protein identifiers, blocks represent conserved protein motifs. Logos visualize motifs. The height of a letter indicates its relative frequency at the given position.


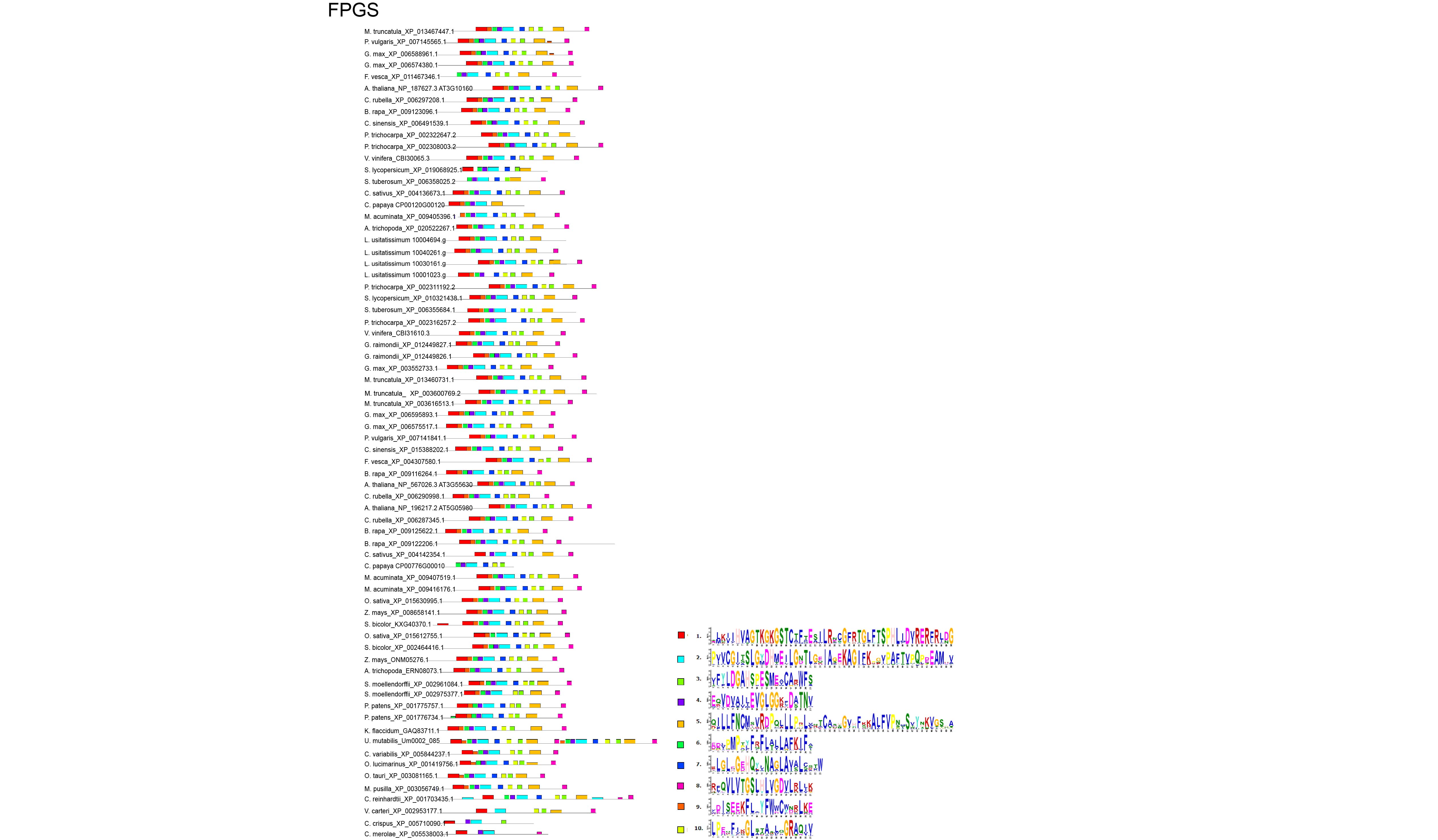


**Supplemental Figure 11.** Conserved protein motif pattern of FPGS.

Species names are followed by protein identifiers, blocks represent conserved protein motifs. Logos visualize motifs. The height of a letter indicates its relative frequency at the given position.


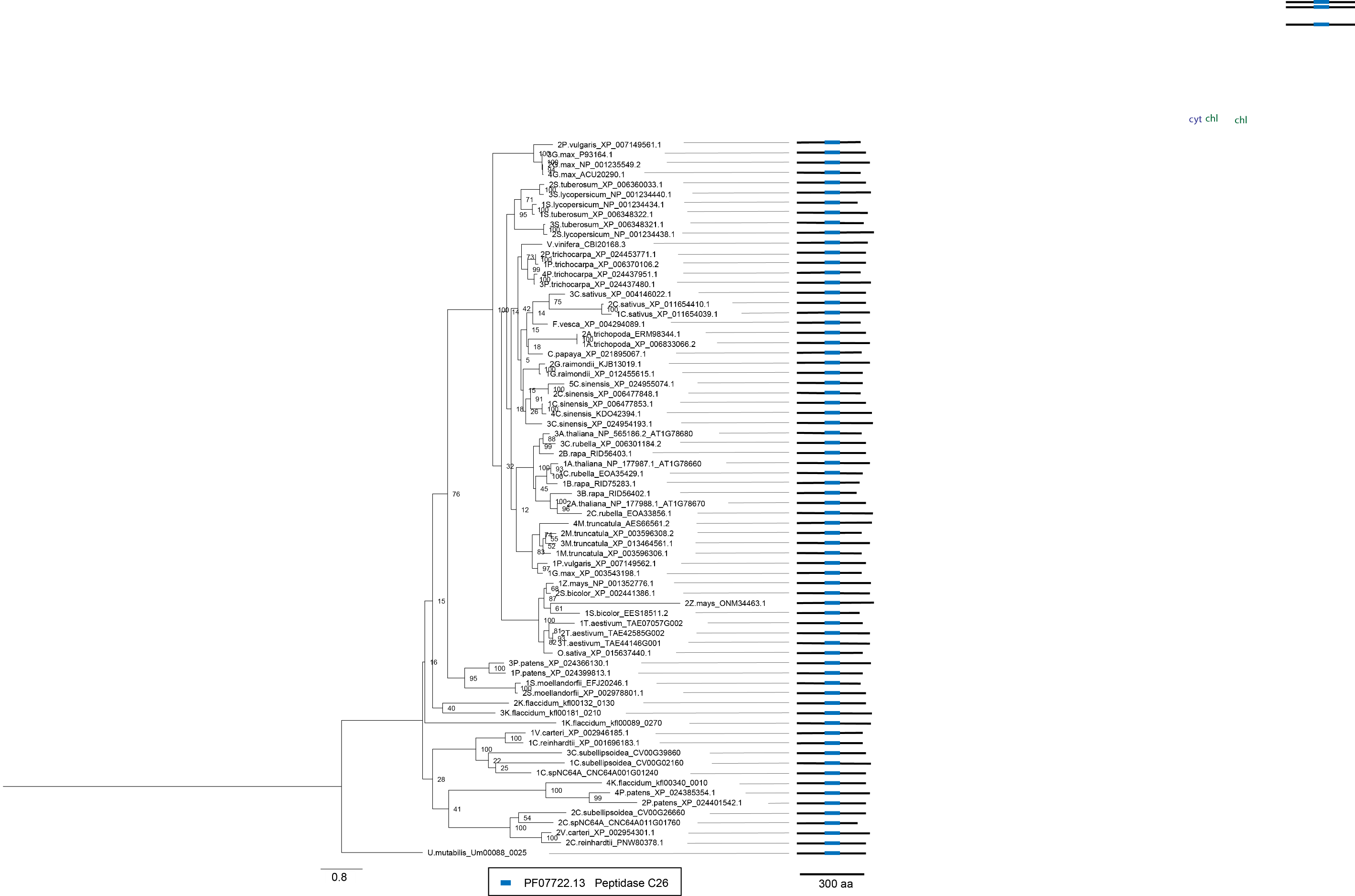


**Supplemental Figure 12.** Phylogenetic analysis, subcellular localization and domain composition of GGH.

Species names are followed by protein identifiers. The bar indicates the mean distance of 0.8 changes per amino acid residue. The numbers at the branching points indicate the percentage of times that each branch topology was found during bootstrap analysis (n=1000). Schemes on the right represent domain organisation of analysed proteins (color boxes represent functional domains, lengths of black lines correspond to lengths of proteins. The scale bar below shows protein containing 300 amino acids). The box contains predicted functional domains.


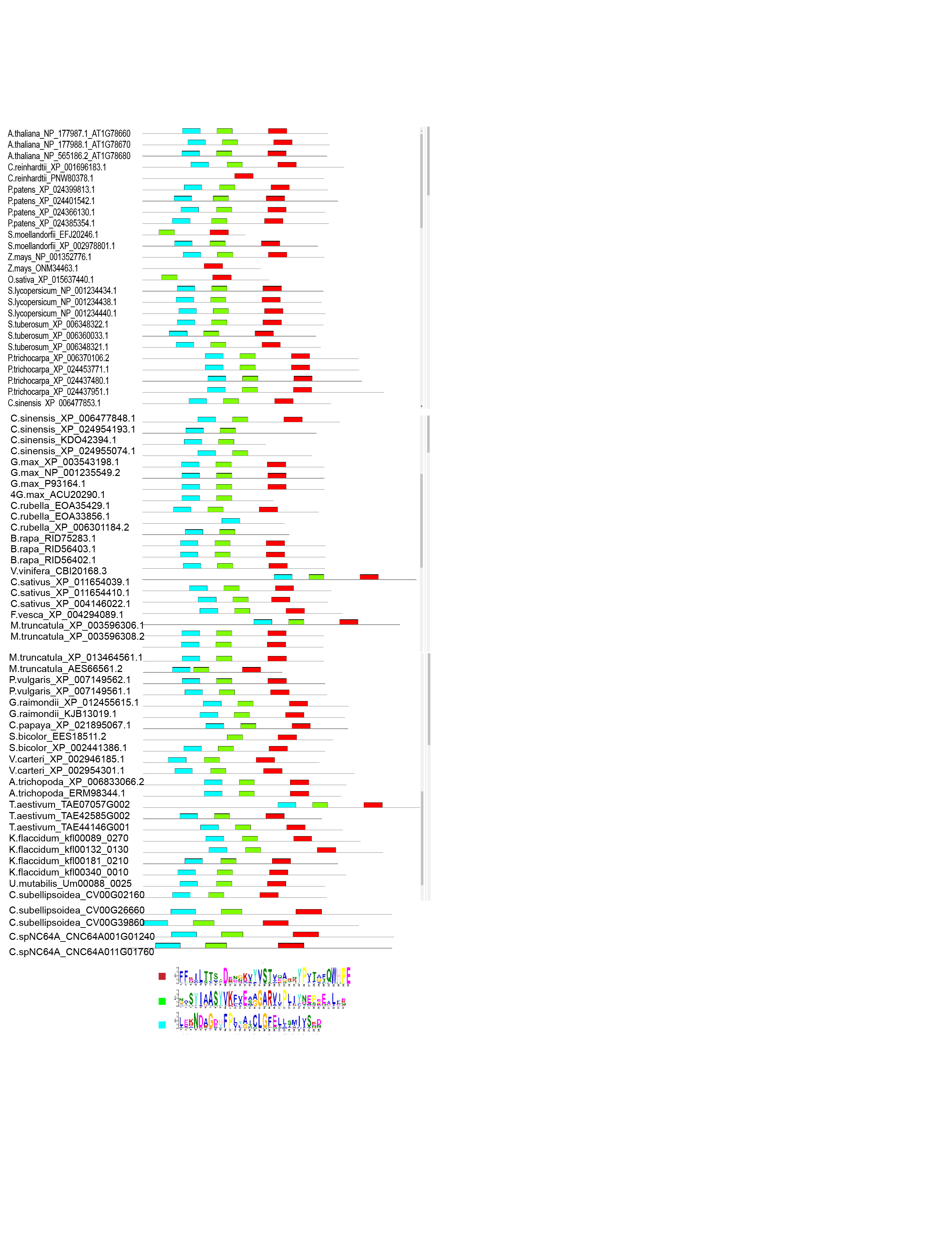


**Supplemental Figure 13.** Conserved protein motif pattern of GGH.

Species names are followed by protein identifiers, blocks represent conserved protein motifs. Logos visualize motifs. The height of a letter indicates its relative frequency at the given position.


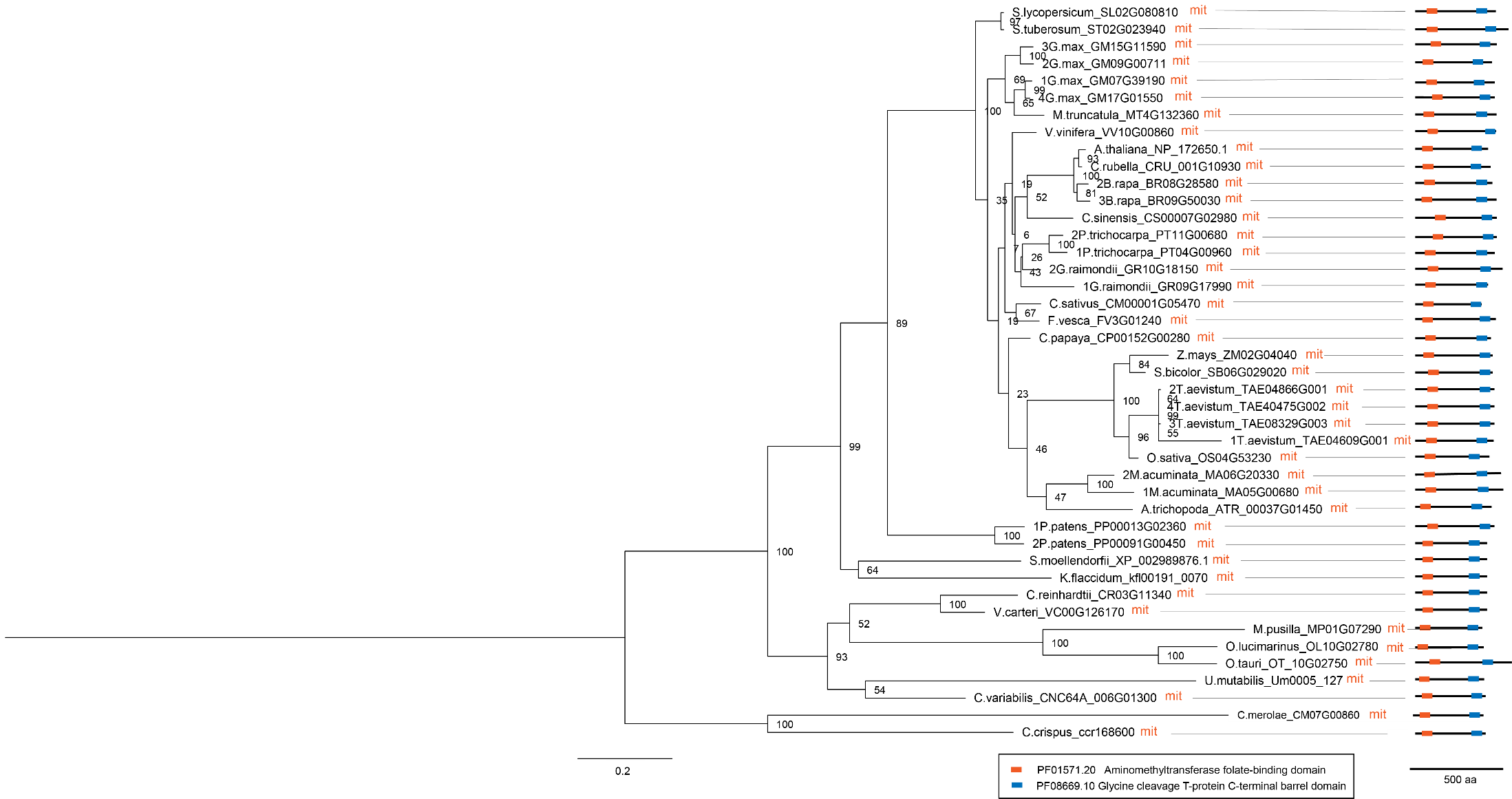


**Supplemental Figure 14.** Phylogenetic analysis, subcellular localization and domain composition of T-protein of GDC complex.

Species names are followed by protein identifiers. The bar indicates the mean distance of 0.2 changes per amino acid residue. The numbers at the branching points indicate the percentage of times that each branch topology was found during bootstrap analysis (n=1000). Schemes on the right represent domain organisation of analysed proteins (color boxes represent functional domains, lengths of black lines correspond to lengths of proteins. The scale bar below shows protein containing 500 amino acids). The box contains predicted functional domains. Mit, mitochondrial localization.


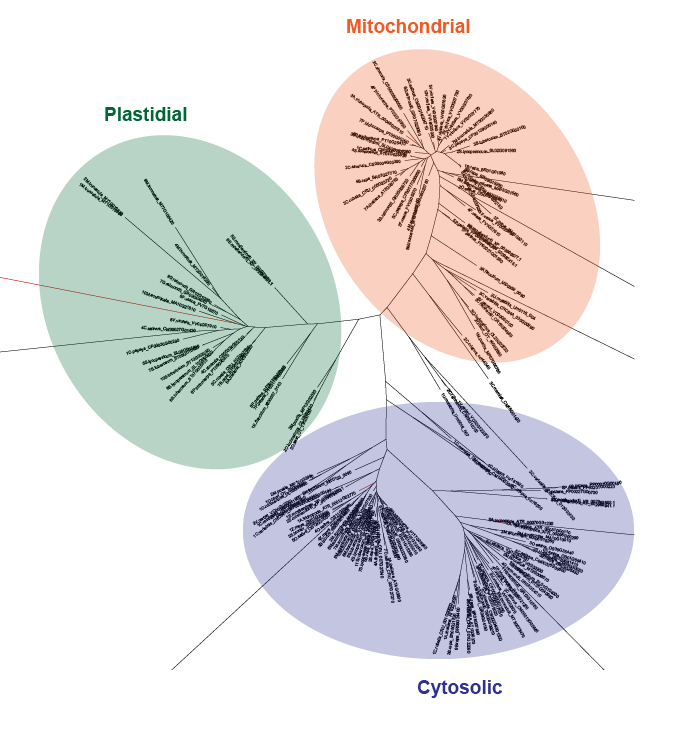


**Supplemental Figure 15** Phylogenetic analysis of SHMT.

**
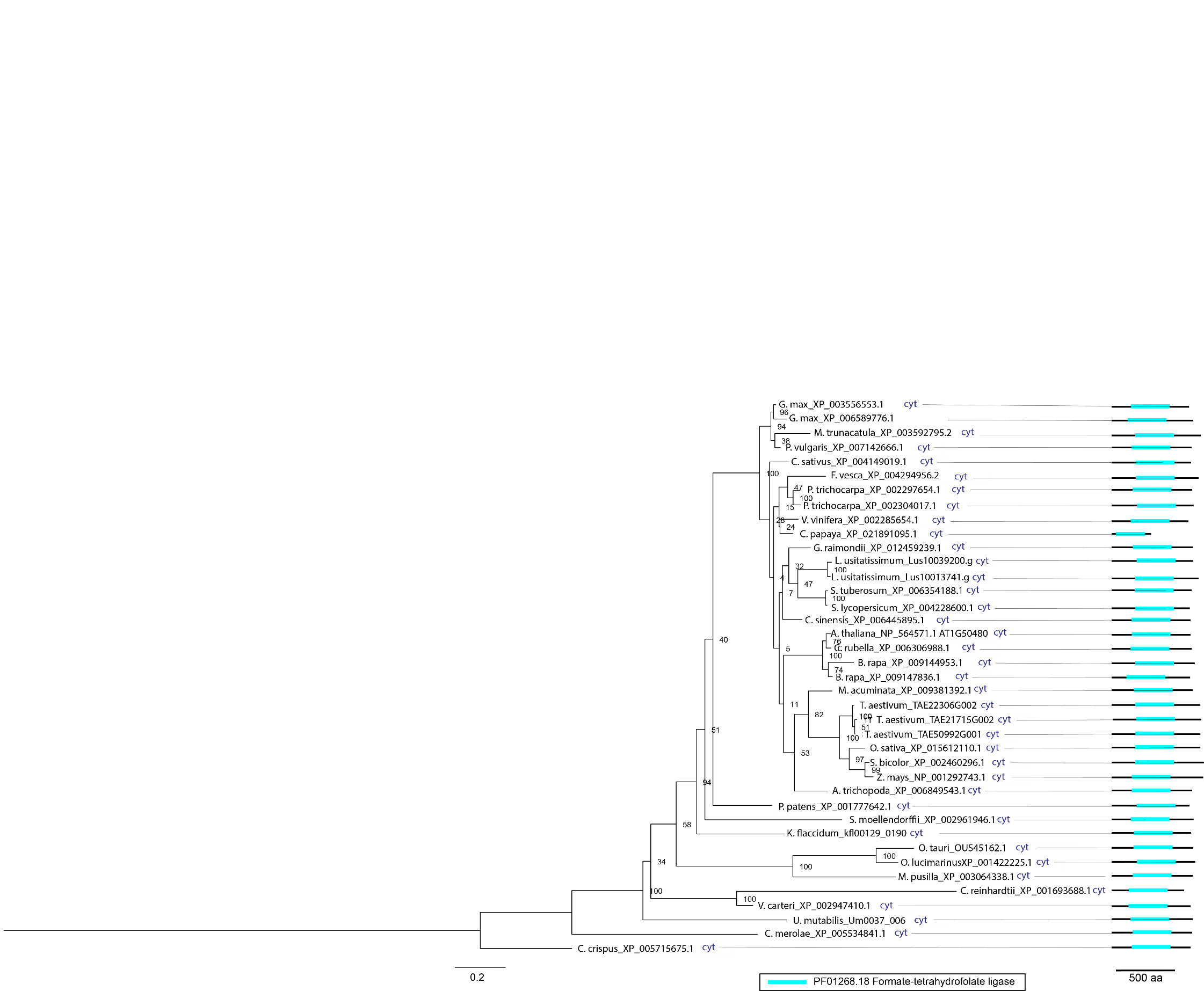
**

**Supplemental Figure 16.** Phylogenetic analysis, subcellular localization and domain composition of FTHFS proteins.

Species names are followed by protein identifiers. The bar indicates the mean distance of 0.2 changes per amino acid residue. The numbers at the branching points indicate the percentage of times that each branch topology was found during bootstrap analysis (n=1000). Schemes on the right represent domain organisation of analysed proteins (color boxes represent functional domains, lengths of black lines correspond to lengths of proteins. The scale bar below shows protein containing 500 amino acids). The box contains predicted functional domains. Cyt, cytosolic localization.


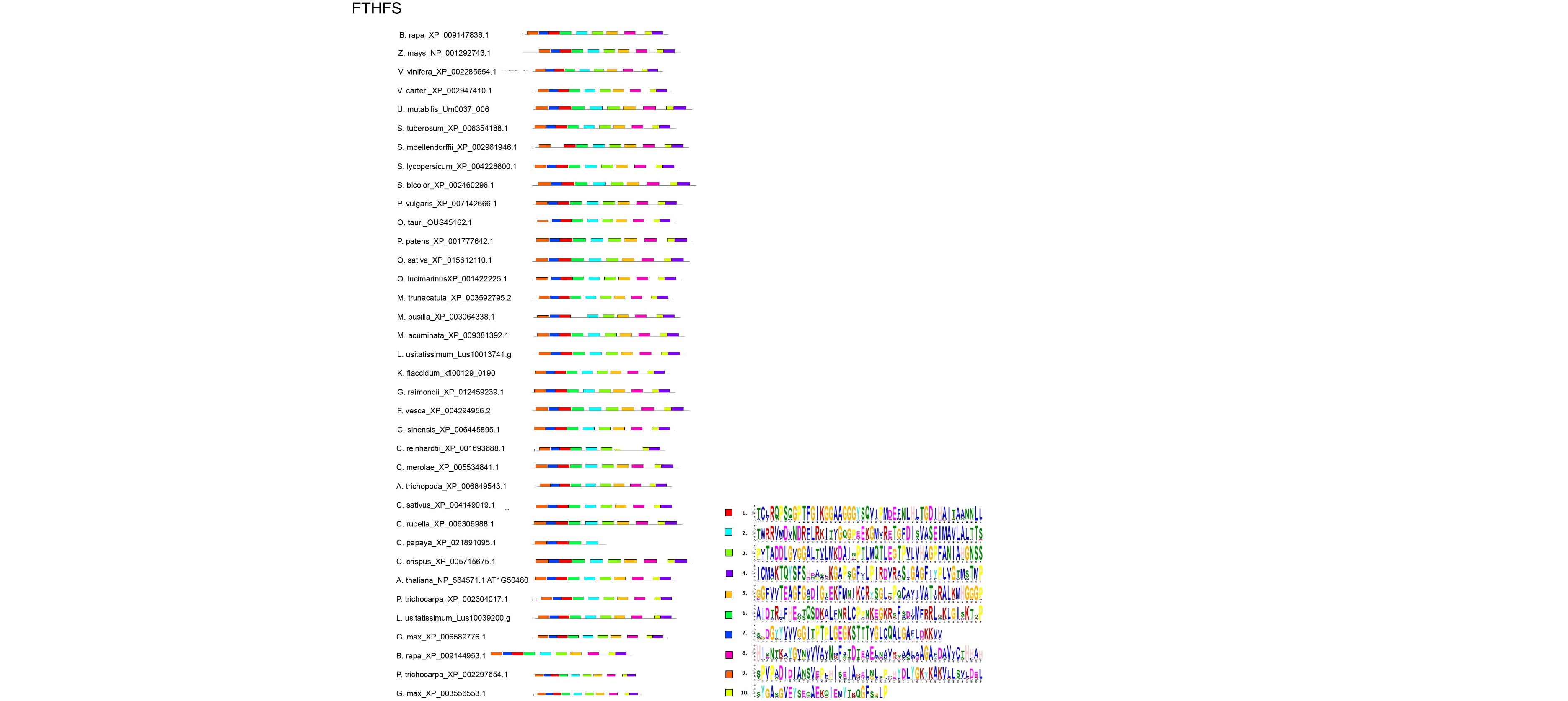


**Supplemental Figure 17.** Conserved protein motif pattern of FTHFS.

Species names are followed by protein identifiers, blocks represent conserved protein motifs. Logos visualize motifs. The height of a letter indicates its relative frequency at the given position.


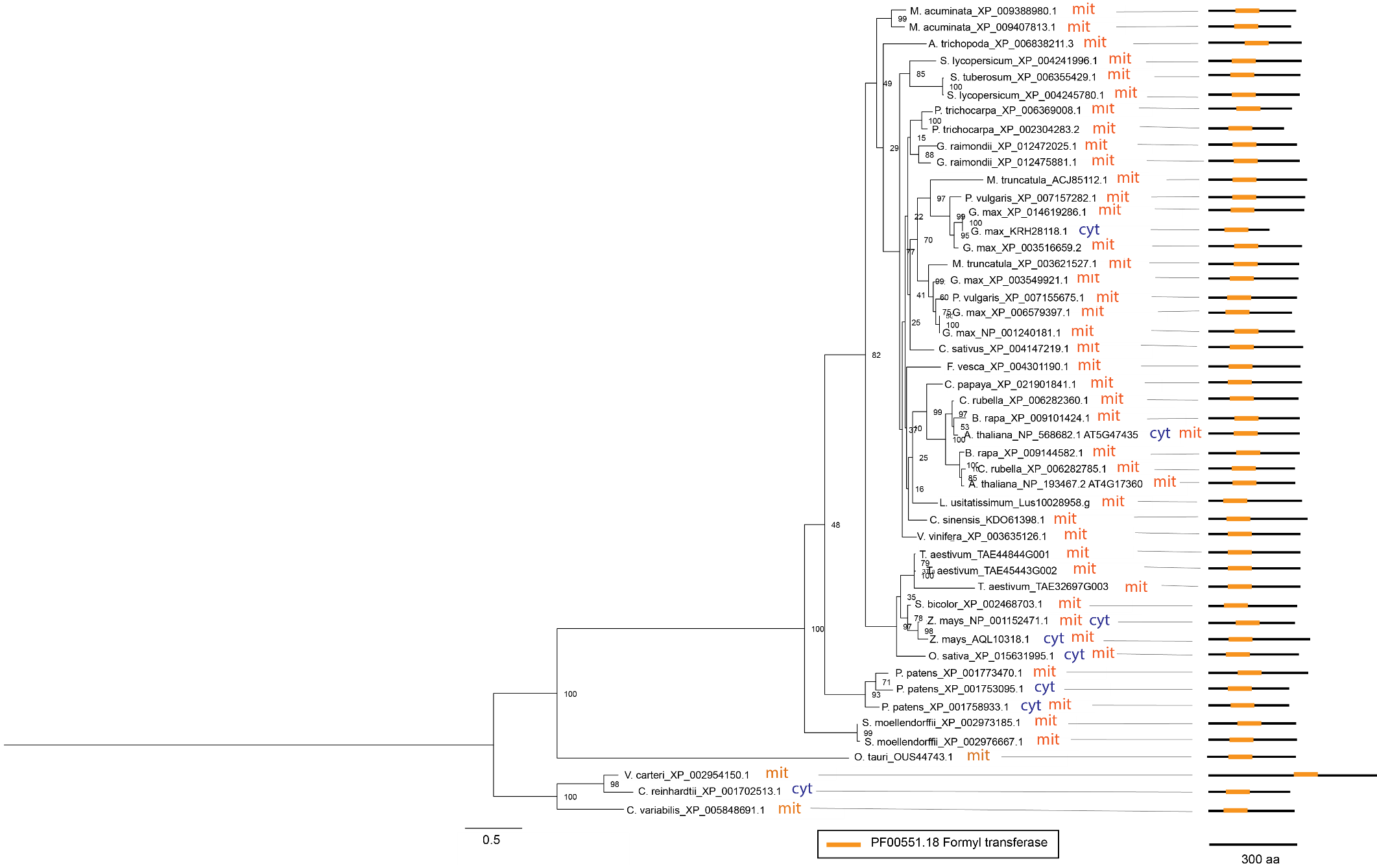


**Supplemental Figure 18.** Phylogenetic analysis, subcellular localization and domain composition of 10-FDF proteins.

Species names are followed by protein identifiers. The bar indicates the mean distance of 0.5 changes per amino acid residue. The numbers at the branching points indicate the percentage of times that each branch topology was found during bootstrap analysis (n=1000). Schemes on the right represent domain organisation of analysed proteins (color boxes represent functional domains, lengths of black lines correspond to lengths of proteins. The scale bar below shows protein containing 300 amino acids). The box contains predicted functional domains. Cyt, cytosolic localization; mit, mitochondrial localization.


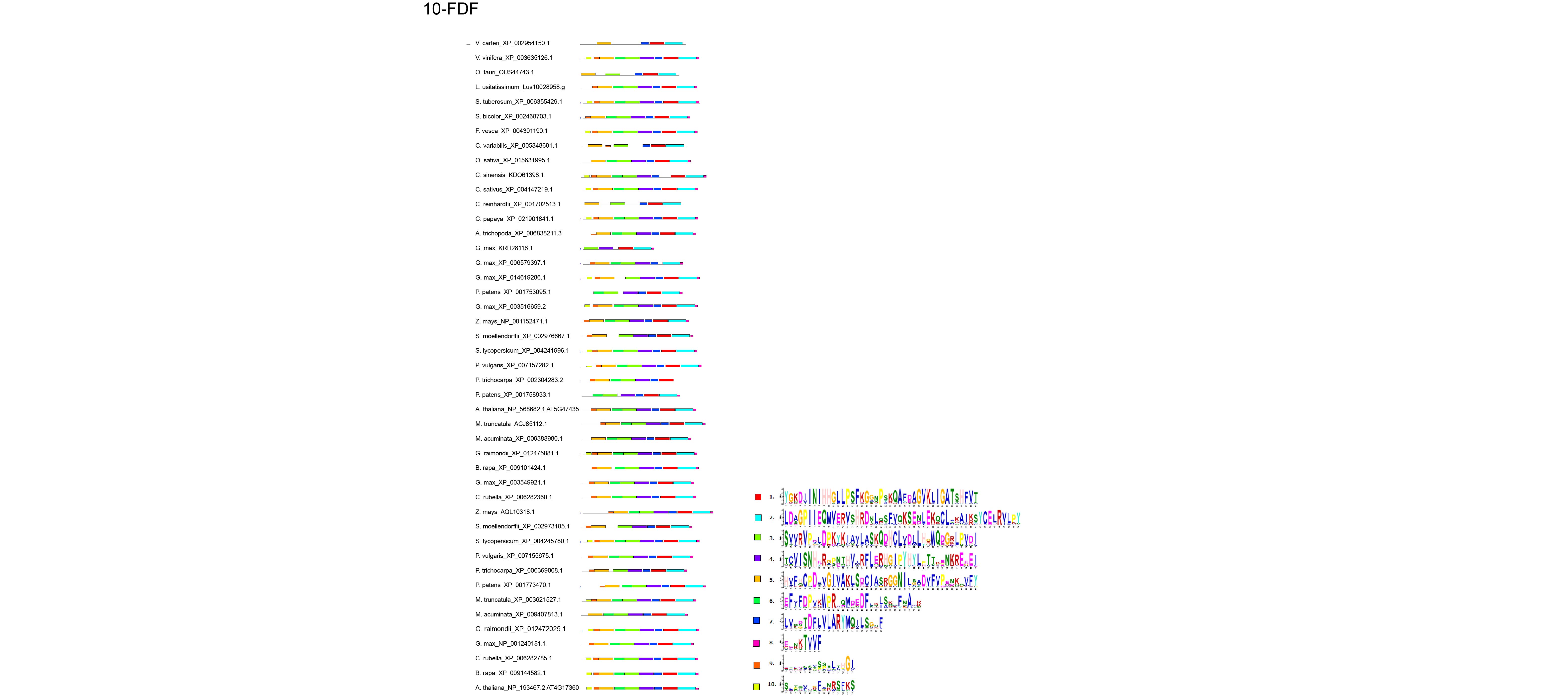


**Supplemental Figure 19.** Conserved protein motif pattern of 10-FDF.

Species names are followed by protein identifiers, blocks represent conserved protein motifs. Logos visualize motifs. The height of a letter indicates its relative frequency at the given position.


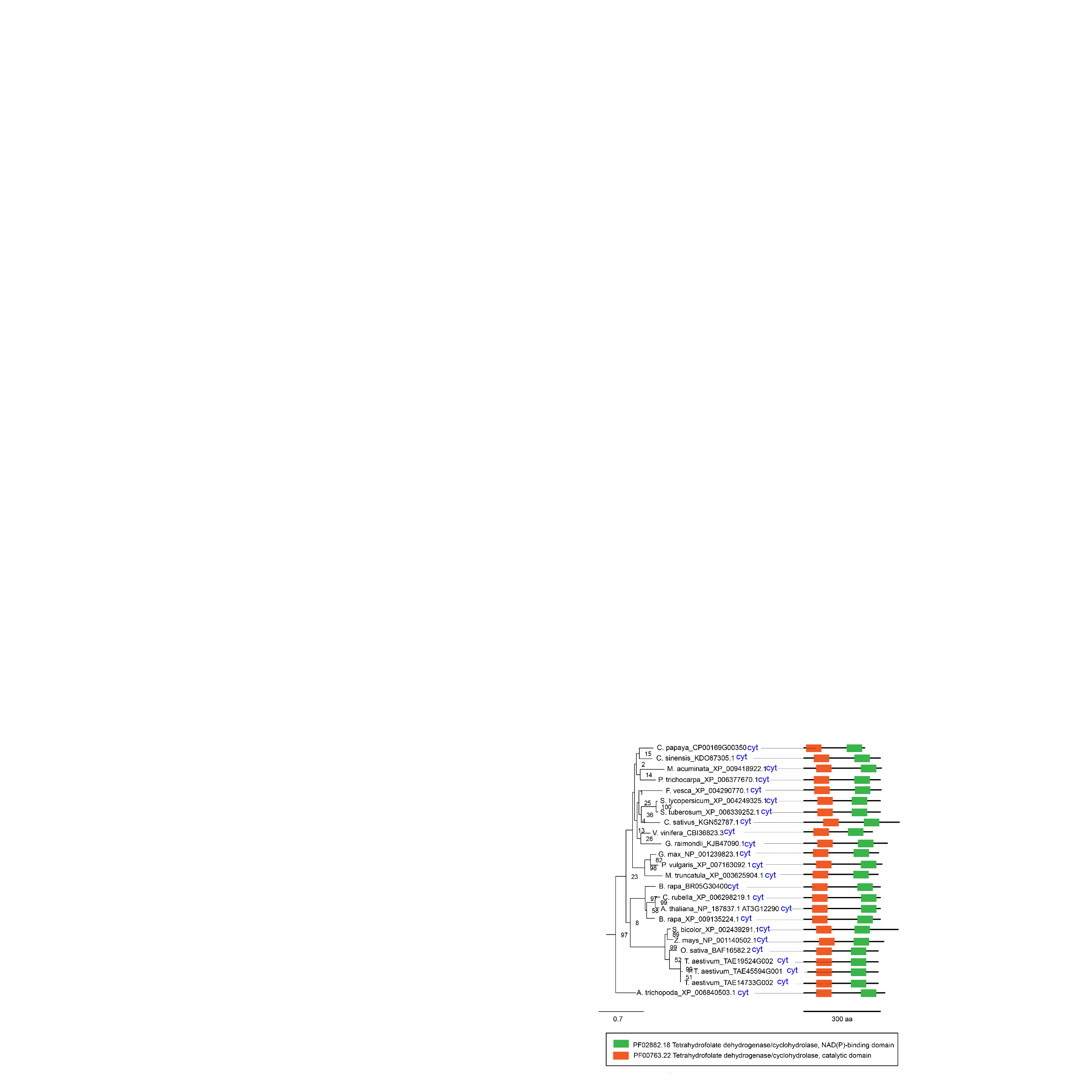


**Supplemental Figure 20.** Phylogenetic analysis, subcellular localization and domain composition of MTHFD-MTHFC1 proteins.

Species names are followed by protein identifiers. The bar indicates the mean distance of 0.7 changes per amino acid residue. The numbers at the branching points indicate the percentage of times that each branch topology was found during bootstrap analysis (n=1000). Schemes on the right represent domain organisation of analysed proteins (color boxes represent functional domains, lengths of black lines correspond to lengths of proteins. The scale bar below shows protein containing 300 amino acids). The box contains predicted functional domains. Cyt, cytosolic localization.

**
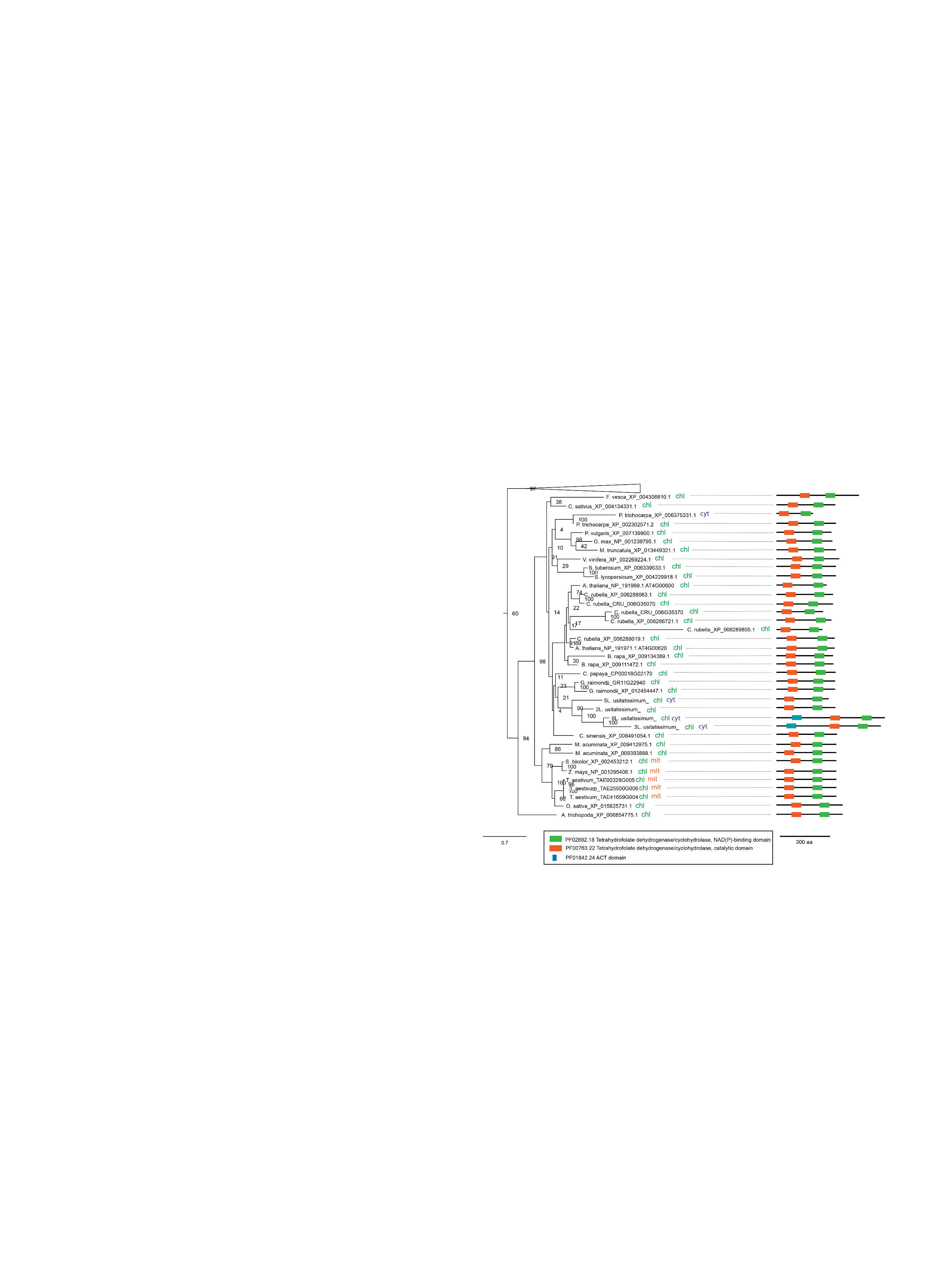
**

**Supplemental Figure 21.** Phylogenetic analysis, subcellular localization and domain composition of MTHFD-MTHFC2 proteins.

Species names are followed by protein identifiers. The bar indicates the mean distance of 0.7 changes per amino acid residue. The numbers at the branching points indicate the percentage of times that each branch topology was found during bootstrap analysis (n=1000). Schemes on the right represent domain organisation of analysed proteins (color boxes represent functional domains, lengths of black lines correspond to lengths of proteins. The scale bar below shows protein containing 300 amino acids. The box contains predicted functional domains. Cyt, cytosolic localization; chl, plastidial localization; mit, mitochondrial localization.


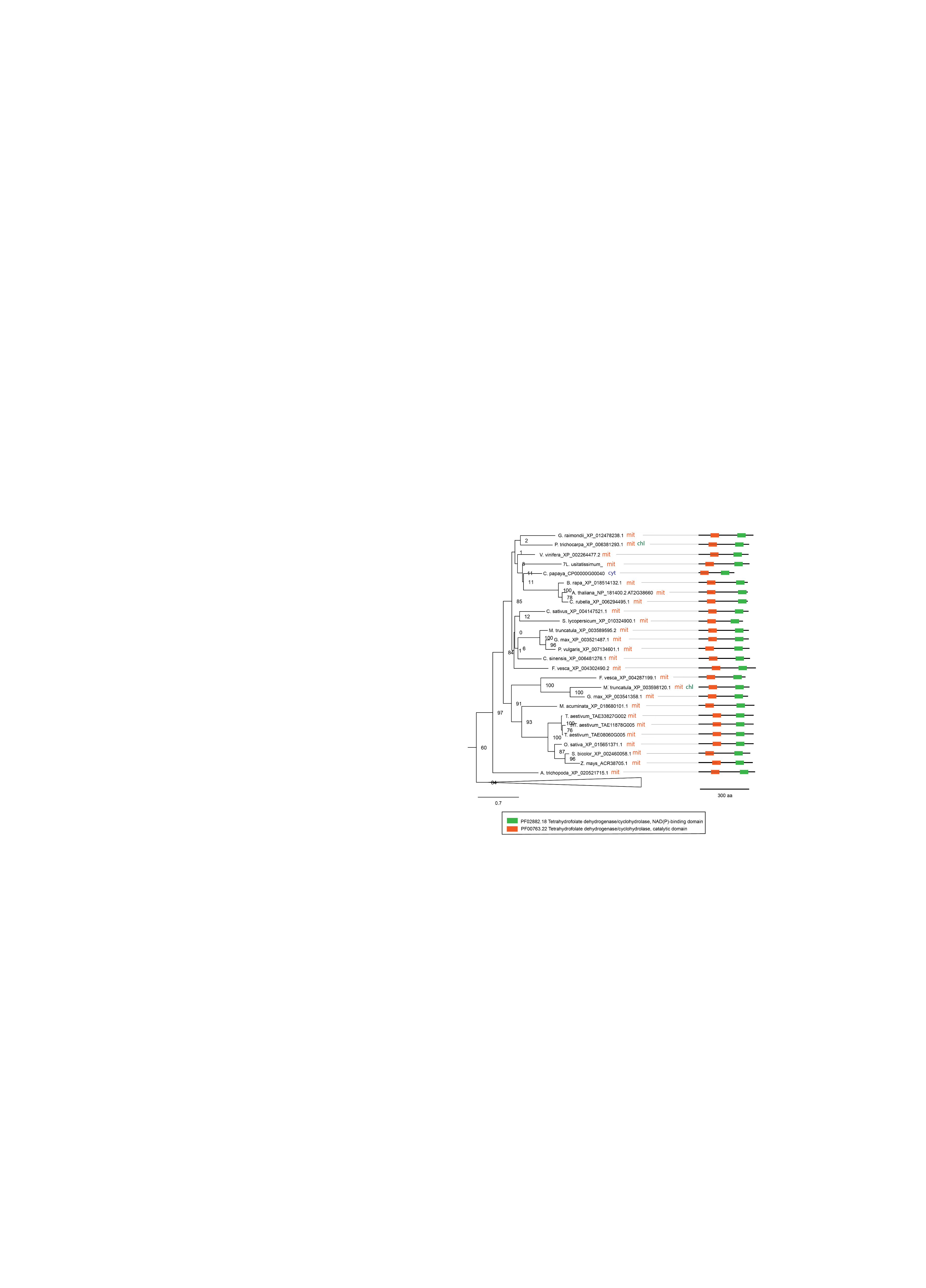


**Supplemental Figure 22.** Phylogenetic analysis, subcellular localization and domain composition of MTHFD-MTHFC3 proteins.

Species names are followed by protein identifiers. The bar indicates the mean distance of 0.7 changes per amino acid residue. The numbers at the branching points indicate the percentage of times that each branch topology was found during bootstrap analysis (n=1000). Schemes on the right represent domain organisation of analysed proteins (color boxes represent functional domains, lengths of black lines correspond to lengths of proteins. The scale bar below shows protein containing 300 amino acids). The box contains predicted functional domains. Cyt, cytosolic localization; chl, plastidial localization; mit, mitochondrial localization.


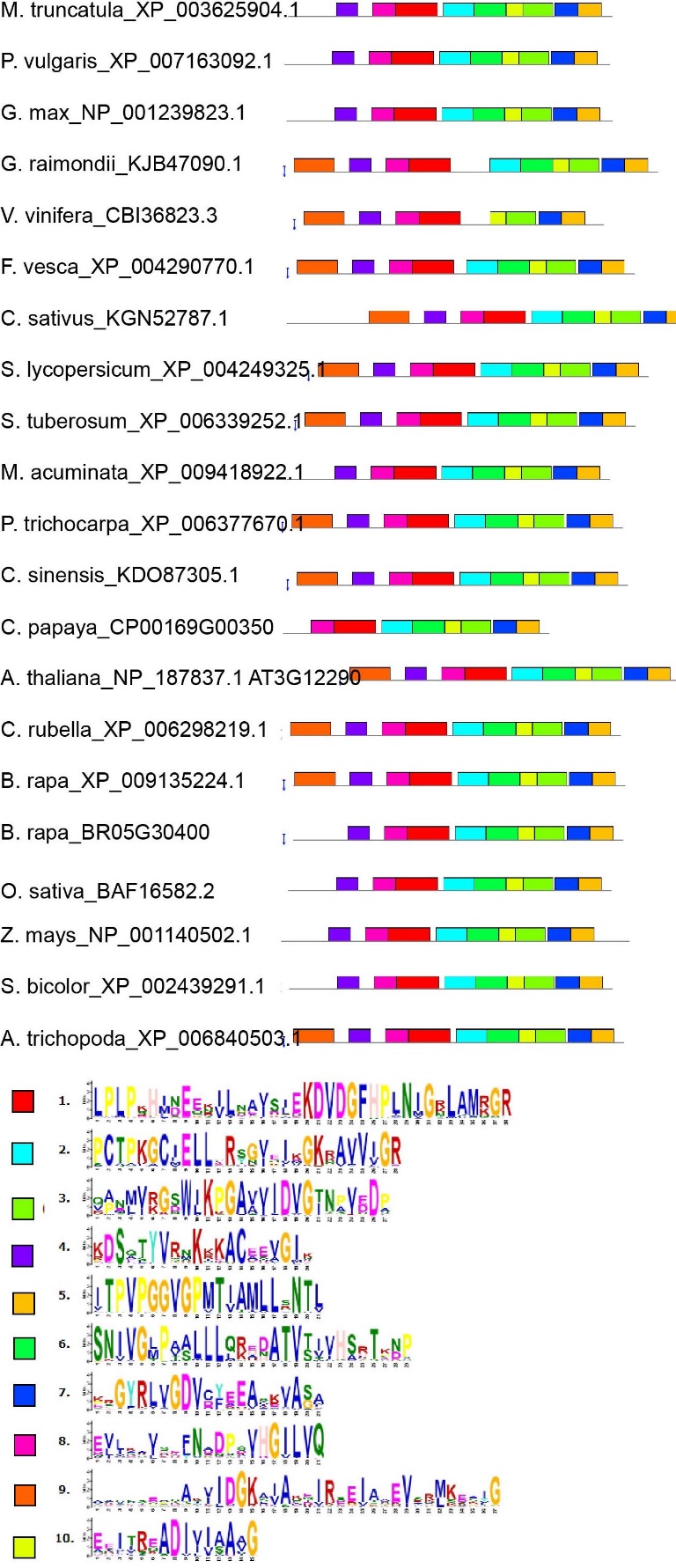


**Supplemental Figure 23.** Conserved protein motif pattern of MTHFD-MTHFC1.

Species names are followed by protein identifiers, blocks represent conserved protein motifs. Logos visualize motifs. The height of a letter indicates its relative frequency at the given position.


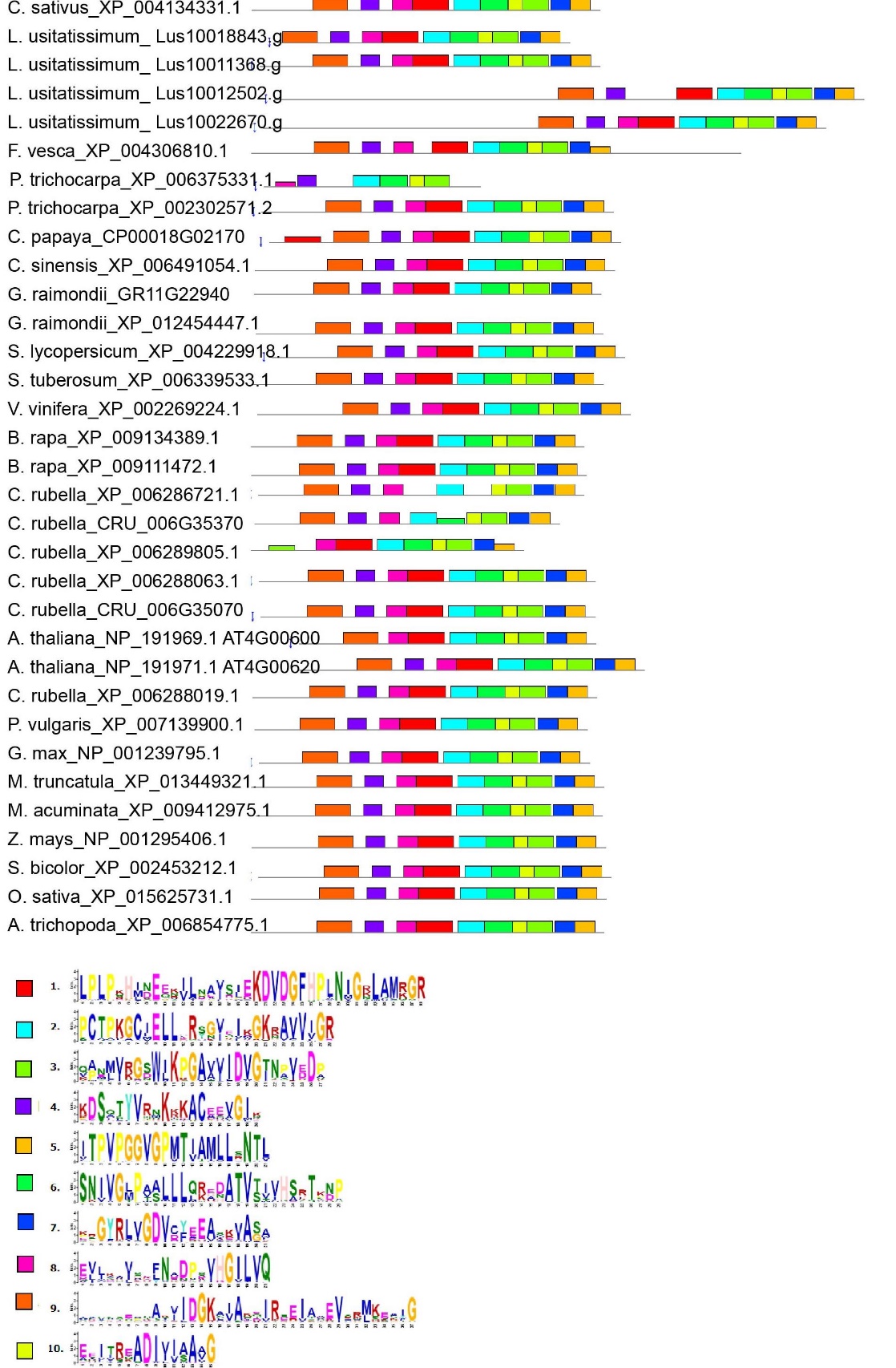


**Supplemental Figure 24.** Conserved protein motif pattern of MTHFD-MTHFC2.

Species names are followed by protein identifiers, blocks represent conserved protein motifs. Logos visualize motifs. The height of a letter indicates its relative frequency at the given position.


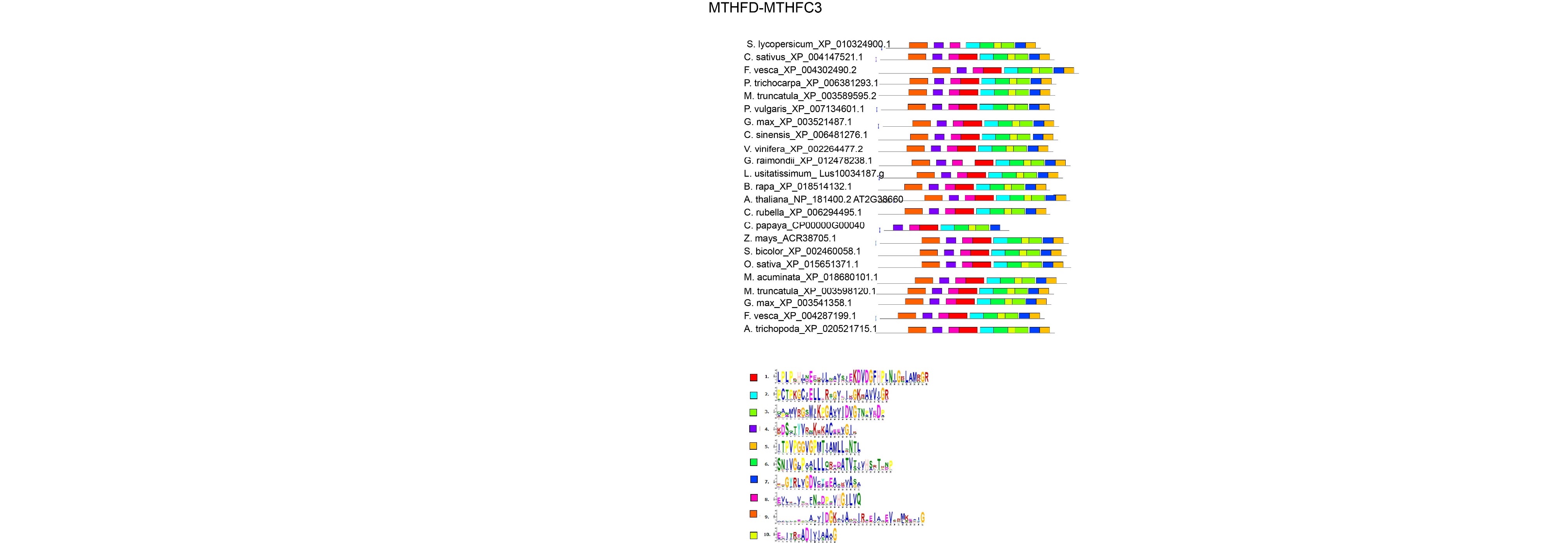


**Supplemental Figure 25.** Conserved protein motif pattern of MTHFD-MTHFC3.

Species names are followed by protein identifiers, blocks represent conserved protein motifs. Logos visualize motifs. The height of a letter indicates its relative frequency at the given position.


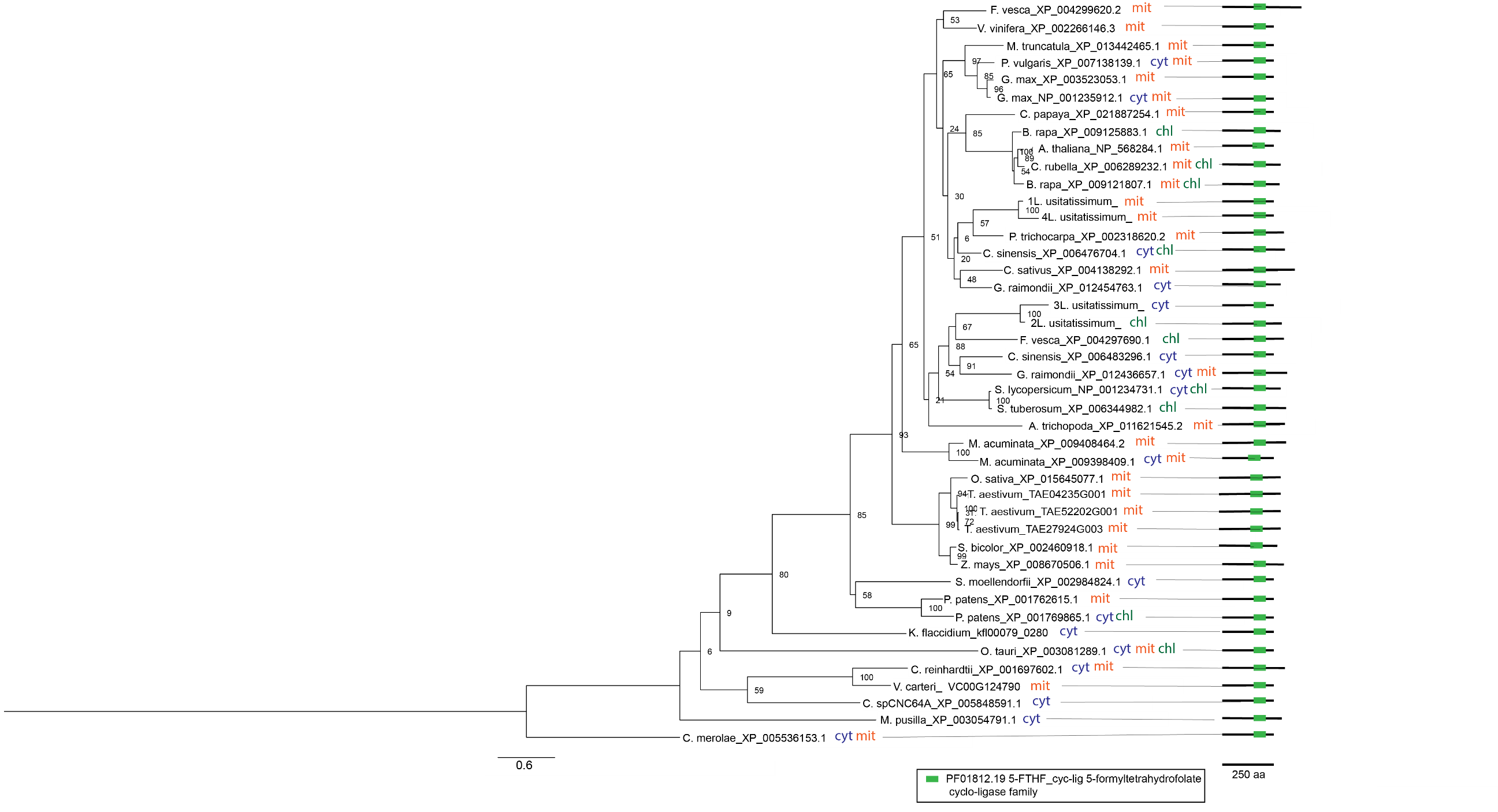


**Supplemental Figure 26.** Phylogenetic analysis, subcellular localization and domain composition of 5-FCL proteins.

Species names are followed by protein identifiers. The bar indicates the mean distance of 0.6 changes per amino acid residue. The numbers at the branching points indicate the percentage of times that each branch topology was found during bootstrap analysis (n=1000). Schemes on the right represent domain organisation of analysed proteins (color boxes represent functional domains, lengths of black lines correspond to lengths of proteins. The scale bar below shows protein containing 250 amino acids). The box contains predicted functional domains. Cyt, cytosolic localization; chl, plastidial localization; mit, mitochondrial localization.


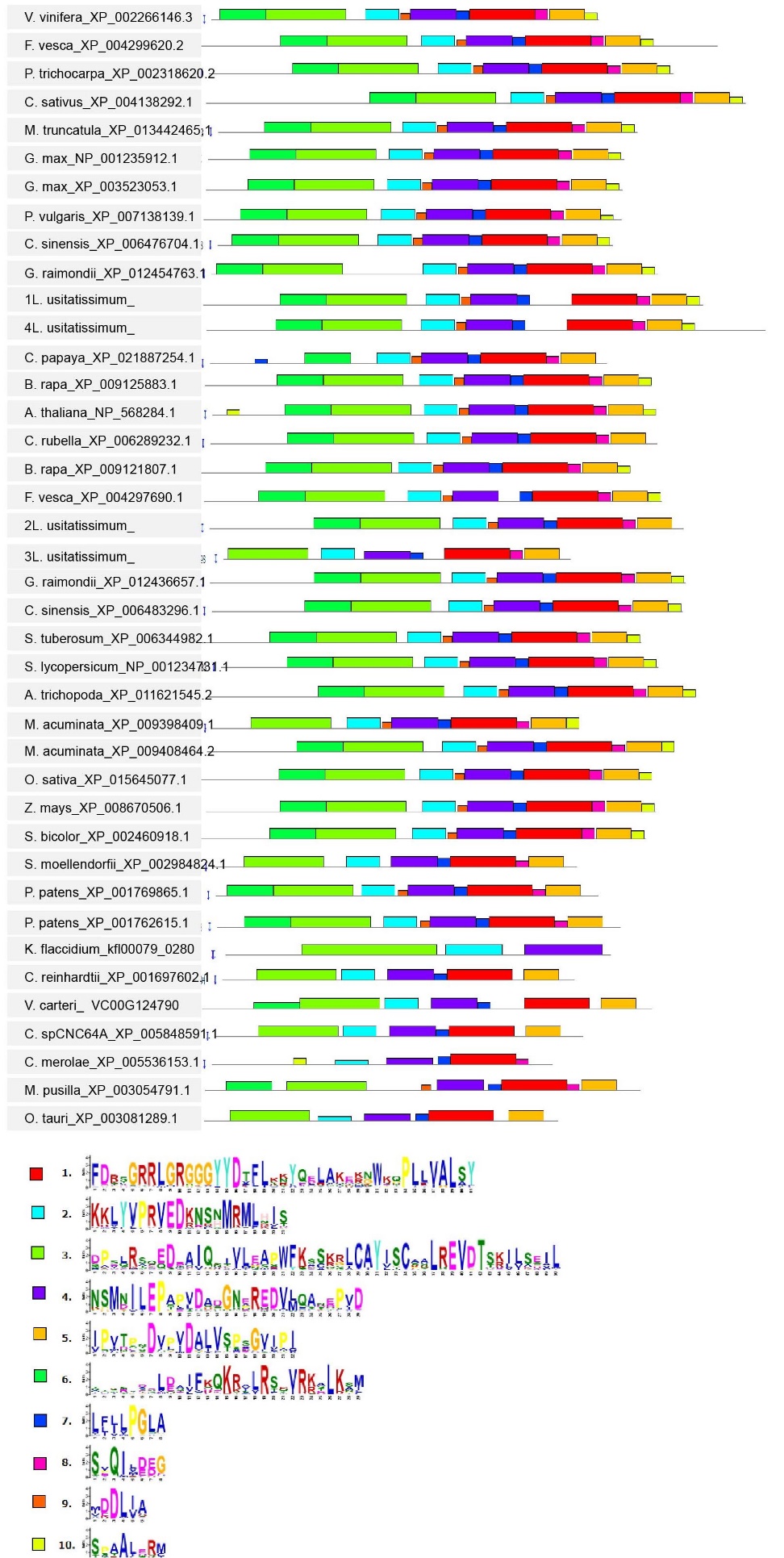


**Supplemental Figure 27.** Conserved protein motif pattern of 5-FCL.

Species names are followed by protein identifiers, blocks represent conserved protein motifs. Logos visualize motifs. The height of a letter indicates its relative frequency at the given position.


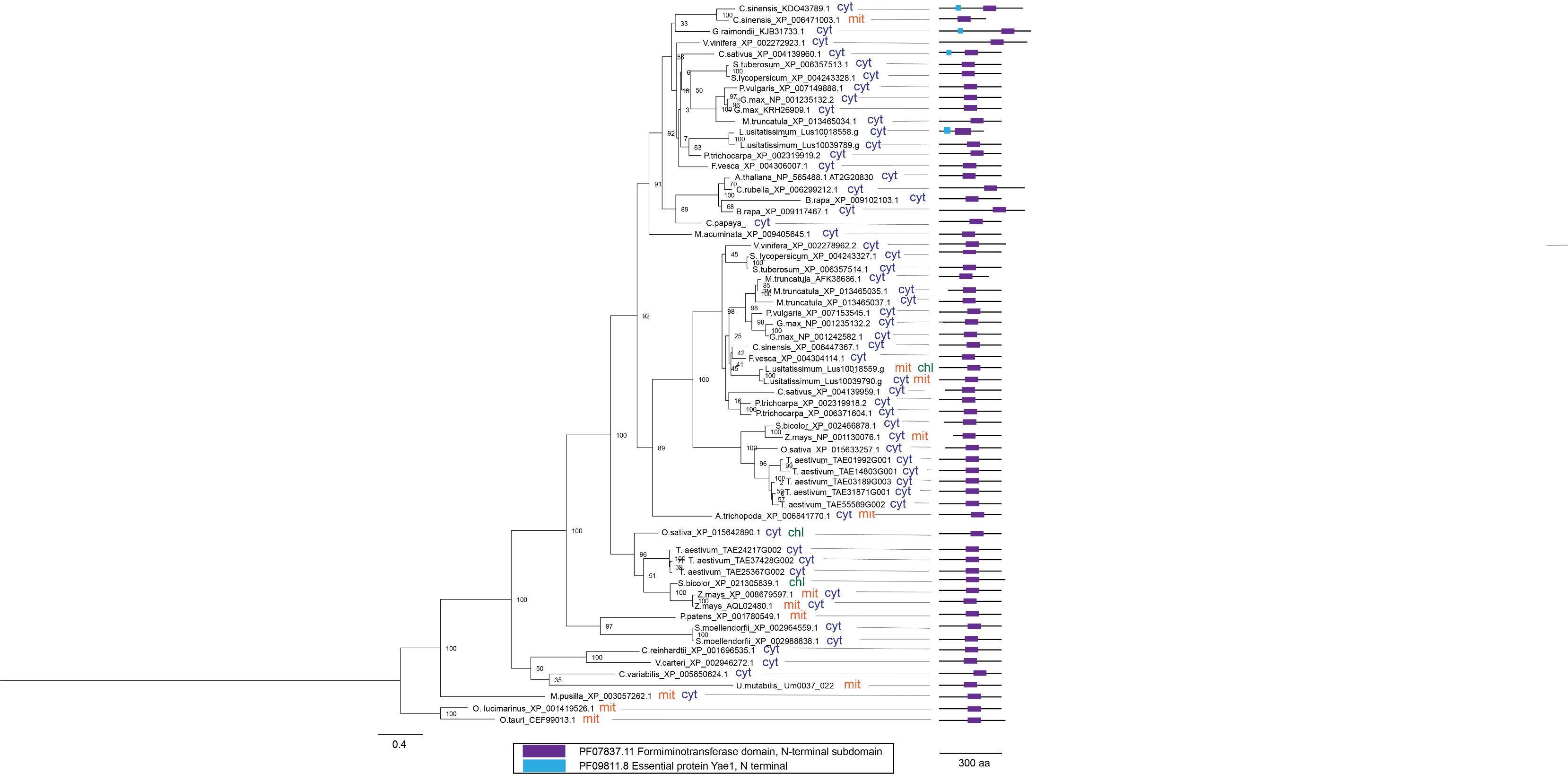


**Supplemental Figure 28.** Phylogenetic analysis, subcellular localization and domain composition of GFT proteins.

Species names are followed by protein identifiers. The bar indicates the mean distance of 0.4 changes per amino acid residue. The numbers at the branching points indicate the percentage of times that each branch topology was found during bootstrap analysis (n=1000). Schemes on the right represent domain organisation of analysed proteins (color boxes represent functional domains, lengths of black lines correspond to lengths of proteins. The scale bar below shows protein containing 300 amino acids). The box contains predicted functional domains. Cyt, cytosolic localization; chl, plastidial localization; mit, mitochondrial localization.


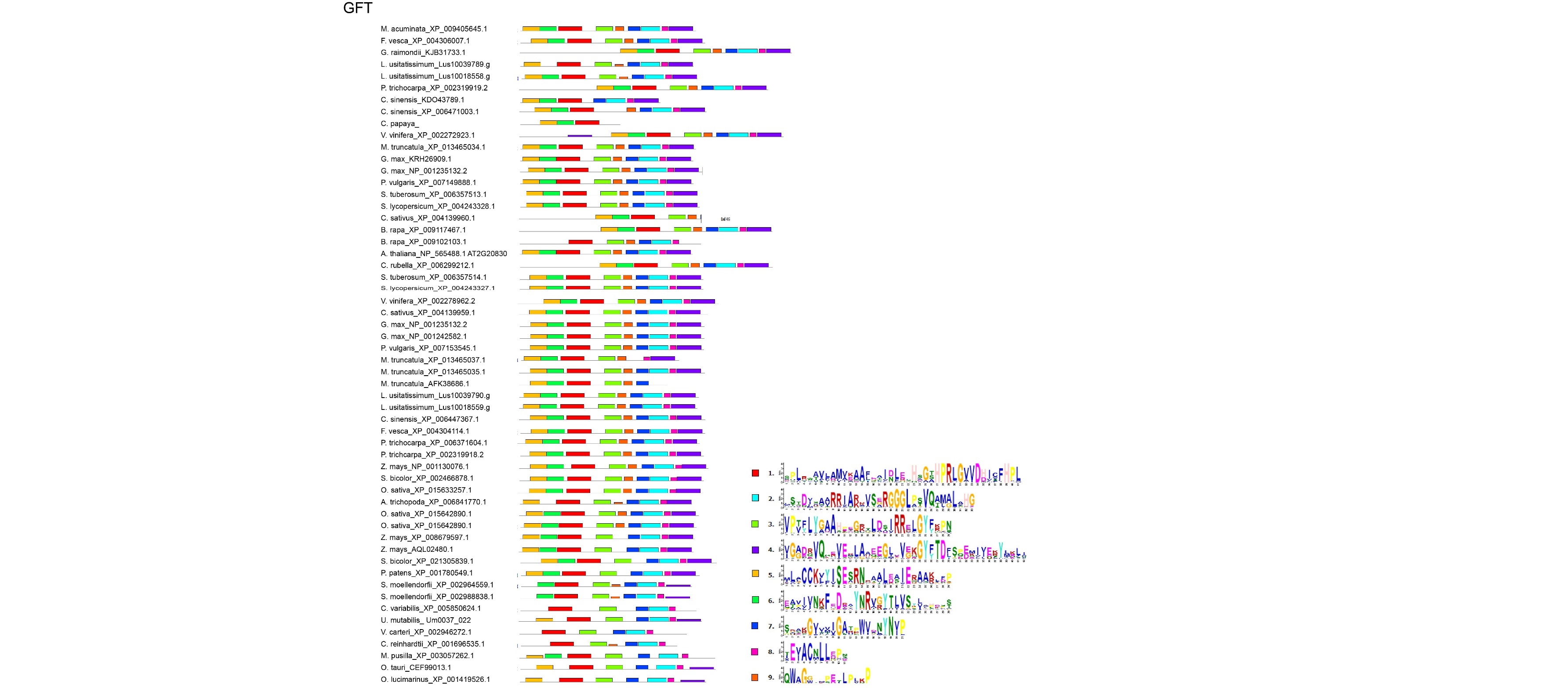


**Supplemental Figure 29.** Conserved protein motif pattern of GFT.

Species names are followed by protein identifiers, blocks represent conserved protein motifs. Logos visualize motifs. The height of a letter indicates its relative frequency at the given position.


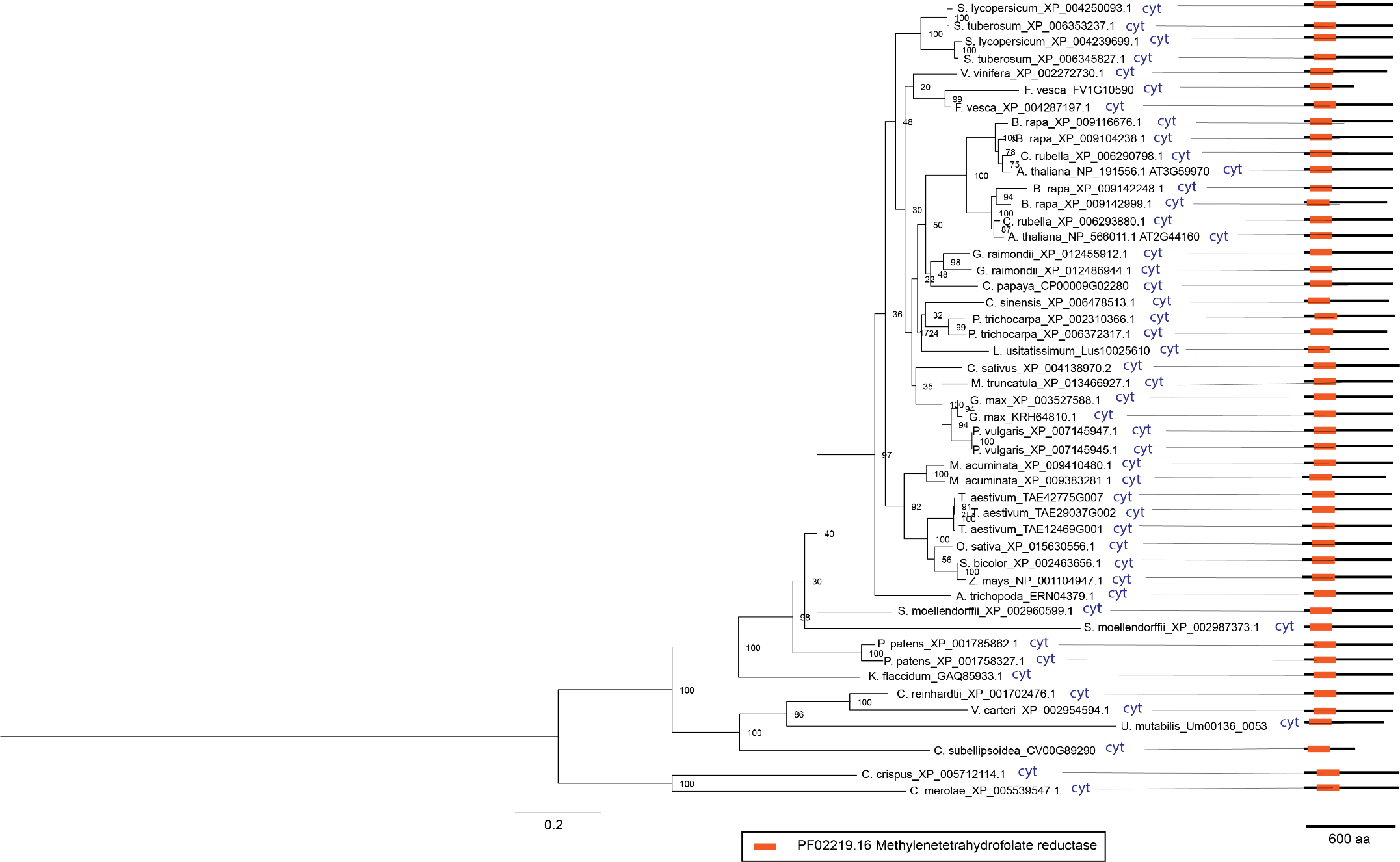


**Supplemental Figure 30.** Phylogenetic analysis, subcellular localization and domain composition of MTHFR proteins.

Species names are followed by protein identifiers. The bar indicates the mean distance of 0.2 changes per amino acid residue. The numbers at the branching points indicate the percentage of times that each branch topology was found during bootstrap analysis (n=1000). Schemes on the right represent domain organisation of analysed proteins (color boxes represent functional domains, lengths of black lines correspond to lengths of proteins. The scale bar below shows protein containing 600 amino acids). The box contains predicted functional domains. Cyt, cytosolic localization.


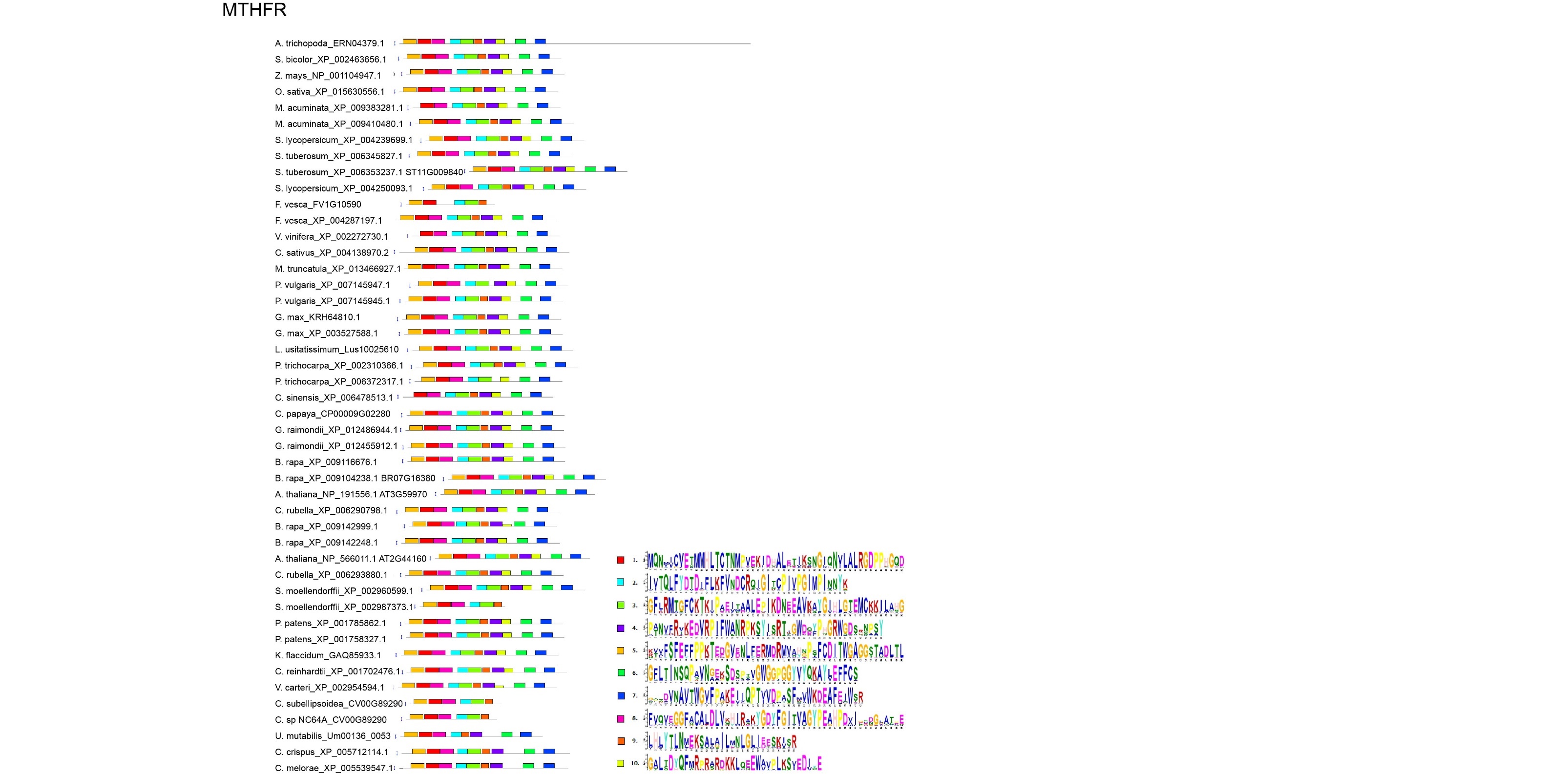


**Supplemental Figure 31.** Conserved protein motif pattern of MTHFR.

Species names are followed by protein identifiers, blocks represent conserved protein motifs. Logos visualize motifs. The height of a letter indicates its relative frequency at the given position.


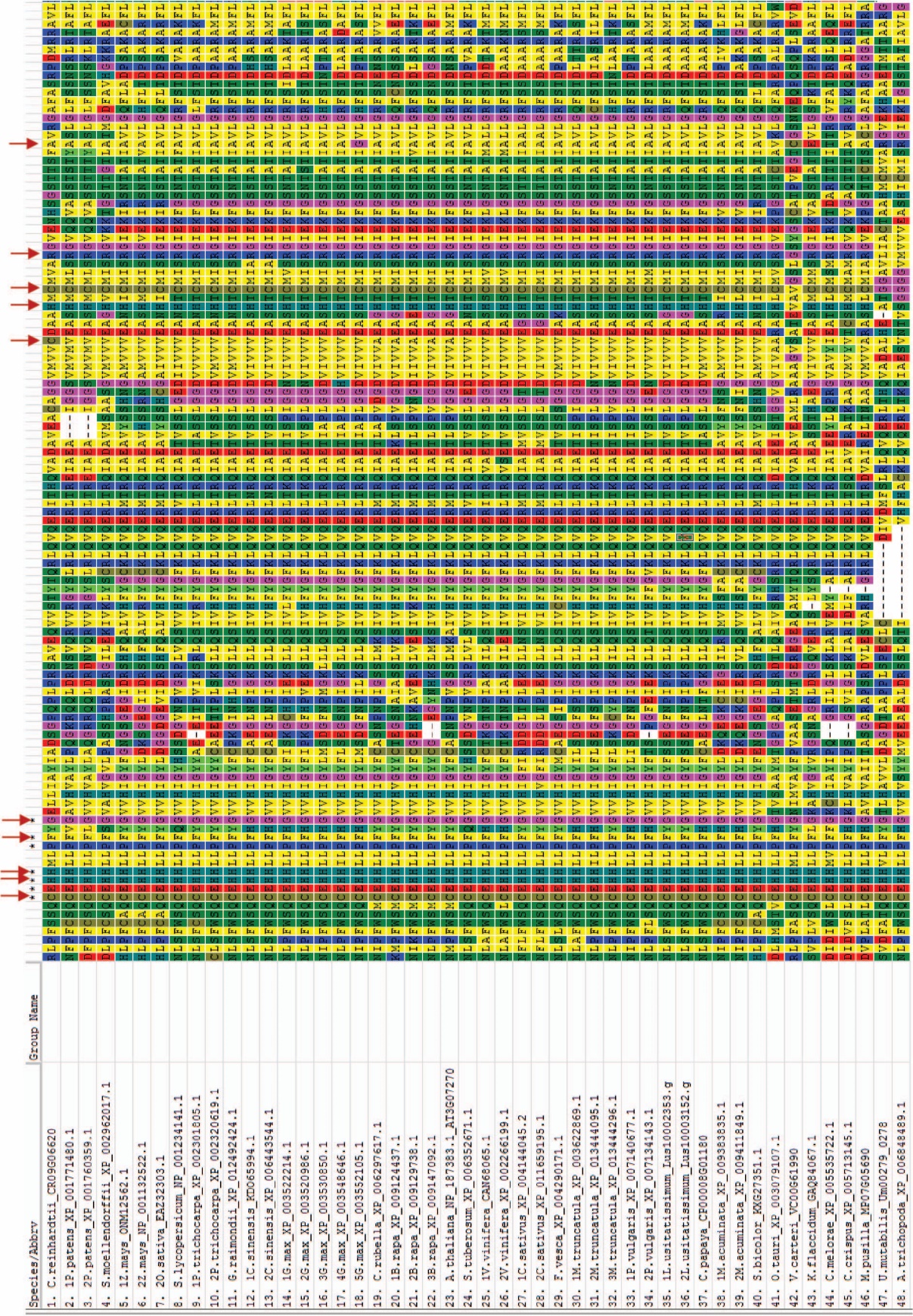

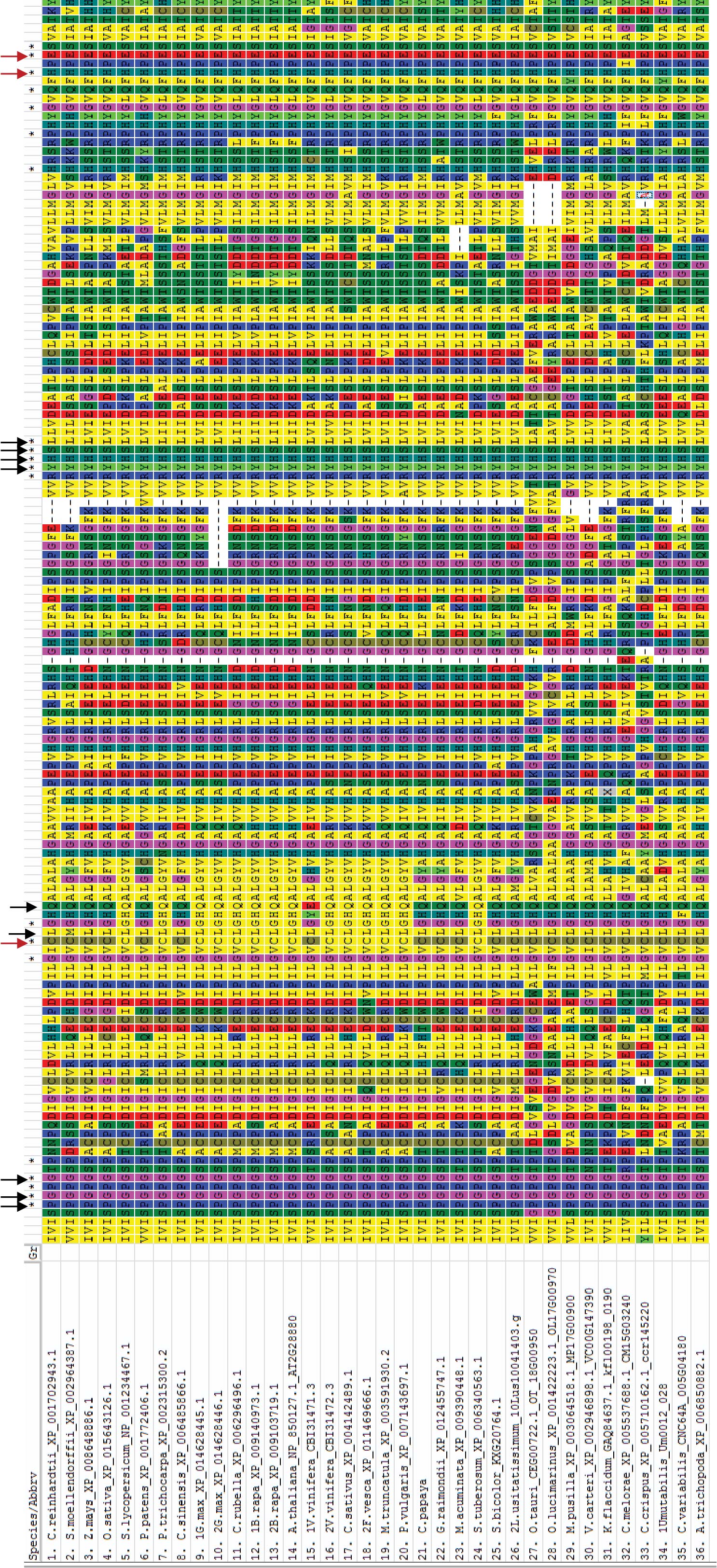


**Supplemental Figure 32.** Conserved residues in GTPCHI.

Alignment is done in MEGA 7. Arrows indicate conserves amino acids important for catalytic activity.

**Supplemental Figure 33.** Conserved residues in ADCS.

Alignment is done in MEGA 7. Arrows indicate conserves amino acids important for catalytic activity.


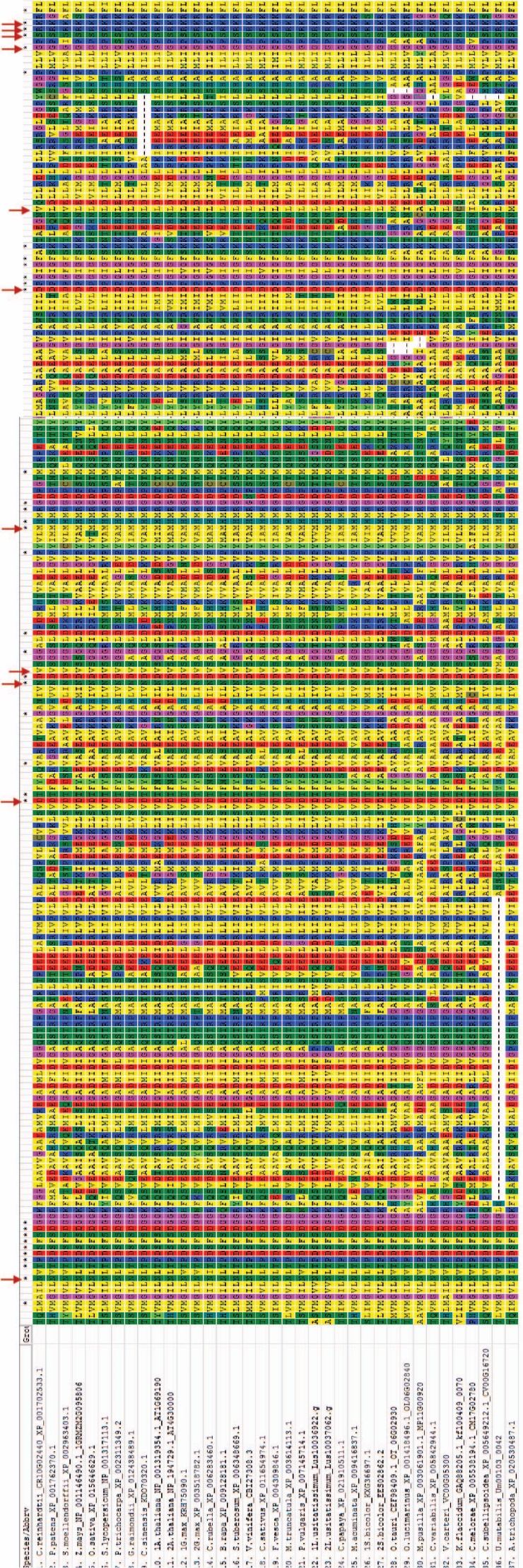


**Supplemental Figure 34.** Conserved residues in DHFS.

Alignment is done in MEGA 7. Arrows indicate conserves amino acids important for catalytic activity.


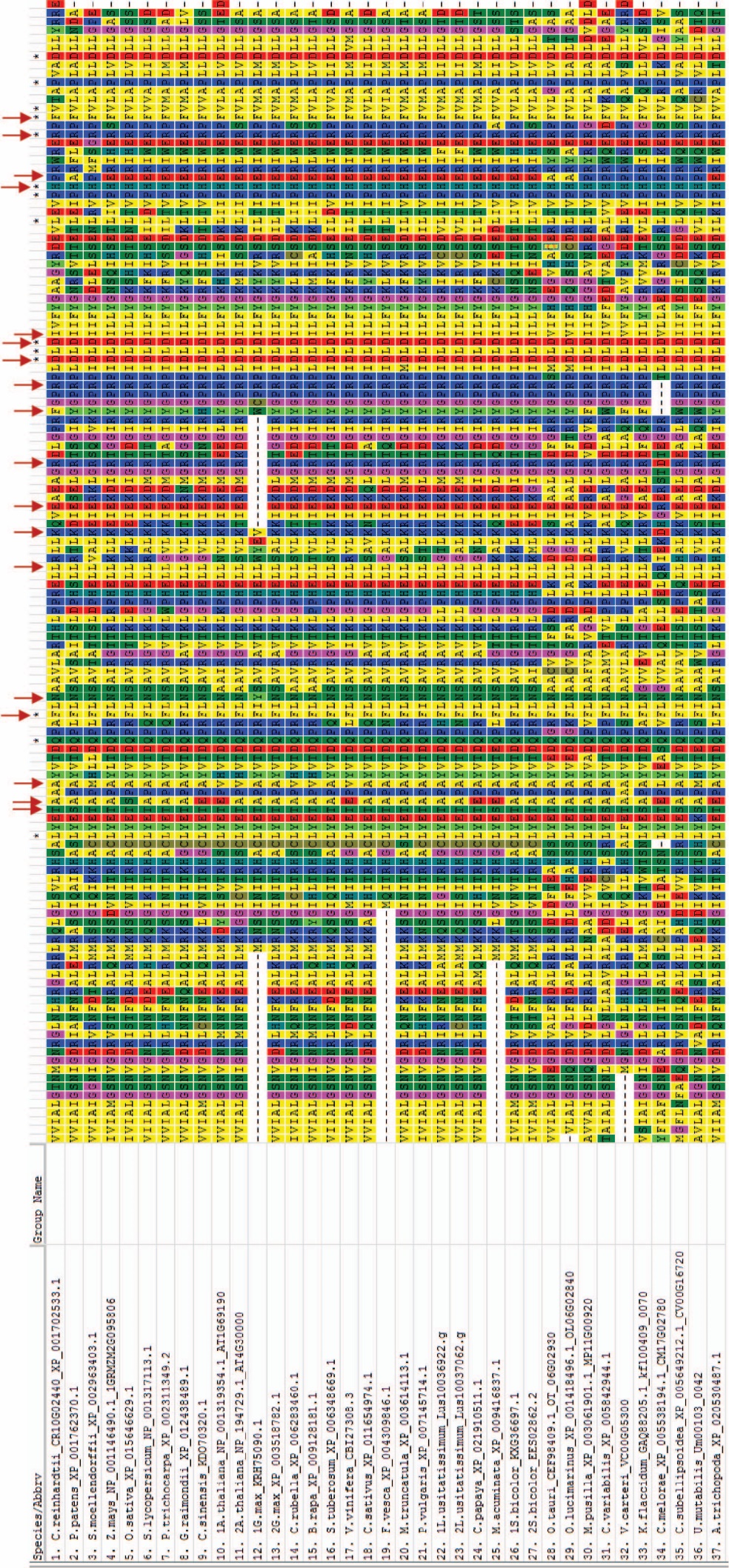


**Supplemental Figure 35.** Conserved residues in HPPK-DHPS.

Alignment is done in MEGA 7. Arrows indicate conserves amino acids important for catalytic activity.


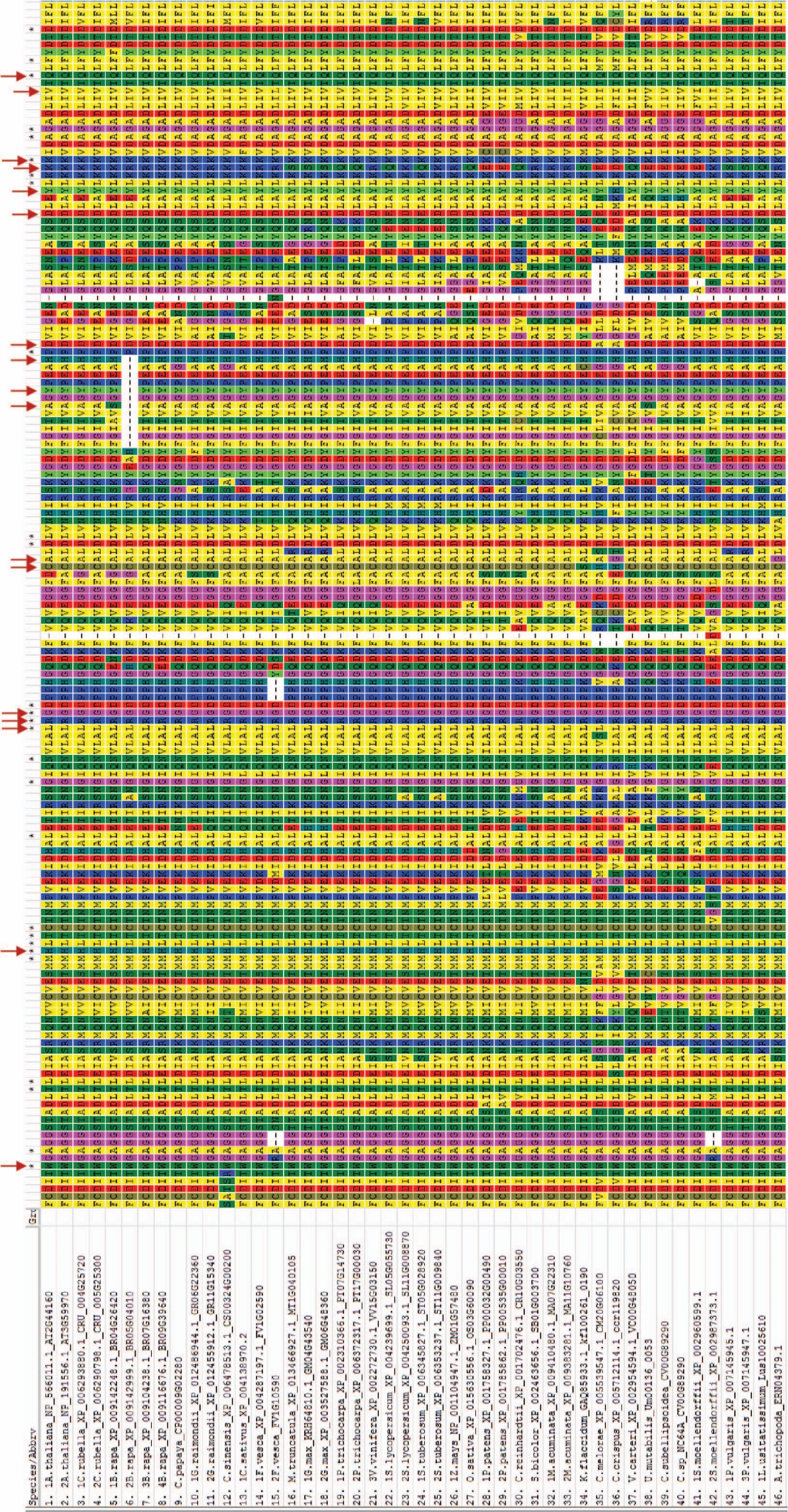


**Supplemental Figure 36.** Conserved residues in MTHFR.

Alignment is done in MEGA 7. Arrows indicate conserves amino acids important for catalytic activity.
